# Microbial adhesion promotes Piezo1 activation to initiate innate immunity

**DOI:** 10.1038/s41590-026-02643-y

**Published:** 2026-09-09

**Authors:** Giulia Stucchi, Marco Galli, Stefano Cozzi, Anna Celant, Laura Marongiu, Giuseppe Rocca, Francesca Colnaghi, Maria Rita Chelazzi, Alessandra Polissi, Alessandra Maria Martorana, Giampiero Pietrocola, Marina Vai, Ivan Orlandi, Renato Ostuni, Simona Barresi, Angelo Lombardo, Metello Innocenti, Francesca Granucci

**Affiliations:** 1 Department of Biotechnology and Biosciences, University of Milano-Bicocca, Piazza della Scienza 2, Milan, Italy; 2 Dipartimento di Scienze Farmacologiche e Biomolecolari, Università degli Studi di Milano, Milan, Italy; 3 Dipartimento di Medicina Molecolare, Università degli Studi di Pavia, Pavia, Italy; 4 San Raffaele-Telethon Institute for Gene Therapy, IRCCS San Raffaele Scientific Institute, Milan, Italy; 5 Vita-Salute San Raffaele University, Milan, Italy

**Keywords:** Innate immunity, Microbiology

## Abstract

How mammals mount an effective immune response against infectious agents remains unresolved. Here we identify microbial adhesion to myeloid cells as a critical initiating event that precedes pattern recognition receptor (PRR) engagement. Using a skin infection model with pathogenic bacteria and fungi, we demonstrate that neutrophil recruitment occurs in two sequential phases. The early phase is PRR-independent and instead driven by microbial adhesion, which engages the mechanosensitive ion channel Piezo1 to promote leukotriene (LT)B_4_ production. Together with interleukin-1α, LTB_4_ induces CXCL1 release, triggering neutrophil infiltration via the same circuit at play during sterile inflammation. By contrast, the late phase is toll-like receptor (TLR)- and CXCL2-dependent, marking a transition to the canonical, pathogen-driven response. Our findings uncover microbial adhesion as a previously unrecognized danger signal that activates innate immunity via mechanotransduction, revealing a paradigm of how immune responses to infection are initiated.

## Abstract (web summary)

Granucci and colleagues identify microbial adhesion to myeloid cells as a critical initiating event that precedes engagement of pattern recognition receptors.

## Main

In the past century, two theories have been formulated to explain the activation of adaptive immune responses, the infectious non-self and non-infectious self (INS) theory by Charlie Janeway^1,2^ and the danger model by Polly Matzinger^3^. The INS theory claims that molecular structures expressed by microbes, dubbed pathogen-associated molecular patterns (PAMPs), are recognized by pattern-recognition receptors (PRRs) expressed by innate immune cells, such as dendritic cells (DCs). Following PRR engagement, DCs are activated, upregulate costimulatory molecules (signal 2) and produce inflammatory cytokines (signal 3), thereby becoming capable of activating T cells. Conversely, the danger model posits that the immune system discriminates between dangerous and non-dangerous rather than self- and non-self antigens. Thus, the expression of costimulatory molecules required for T cell priming is induced in innate antigen-presenting cells (APCs) by distressed host cells that release altered or aberrantly large amounts of danger-associated molecular pattern (DAMP) molecules.

The INS theory^4^ was recently updated with the suggestion that, in successful infections, PRRs might not recognize PAMPs per se but rather only those mistakenly released during infection, such as endosomal or cytosolic PAMPs. Therefore, successful microbes are infectious and evade PRRs, whereas the unsuccessful ones are immunogenic and poorly infectious^5^. Likewise, the danger model has been recently enriched with an additional class of receptors—mechanotransduction receptors—that can convert mechanical signals into biochemical signals. Thus, mechanical stress adds to the repertoire of danger signals that could contribute to activating immune responses^6–8^.

These two theories, initially proposed to explain the activation of the adaptive immune system^9–14^, were then reframed to also explain the activation of innate immune responses, particularly the inflammatory process triggered upon infection.

This crystallized into the coincident recognition theory, which postulates that a combination of DAMPs, for instance, altered self-molecules released after tissue injury, usually before infection, and PAMPs provided by microorganisms during infection would ignite persistent tissue inflammation^13^. If sustained long enough, this state would lead to the activation of APCs and adaptive immunity.

During the inflammatory process, the recruitment of circulating neutrophils into tissues is a fundamental early event^15–18^ and has been linked with the rapid containment of infections^19^. Neutrophil recruitment occurs even in cases of sterile inflammation^20^, indicating that it could initially serve as a precautionary measure to preemptively combat potential infections. This suggests that the early stages of neutrophil recruitment might be regulated by signaling pathways that operate independently of a specific threat. In the event of an actual infection, this recruitment would continue over time.

This scenario calls into question the role of PRRs: is sensing of PAMPs dispensable during the early recruitment of neutrophils upon microbial infections? How does it contribute to microbial inflammation? The possibility that the presence of microorganisms as such is not immediately perceived by PRRs aligns with the revised INS theory, whereby sensing of microorganisms would occur only in the presence of altered PAMPs^5^, the generation of which would require time.

To investigate the mechanisms regulating neutrophil recruitment upon infection, we utilized a skin infection model wherein microbes are injected intradermally. This approach minimizes tissue damage while introducing large amounts of PAMPs. We used different types of microorganisms: a fungus, *Candida albicans*; a Gram-positive bacterium, *Staphylococcus aureus*; and a Gram-negative bacterium, *Pseudomonas aeruginosa*. Although these microorganisms differ considerably in the PAMPs they express, their invasiveness and the toxins they release, we found that, in the early stages of infection, they induce neutrophil recruitment using a TLR-independent mechanism. The consequent activation of the mechanoreceptor Piezo1 leads to the release of leukotriene B_4_ (LTB_4_), interleukin-1α (IL-1α) and CXCL1 to start the recruitment of neutrophils. This represents the same axis at play during sterile inflammation.

On these grounds, we propose that the first wave of neutrophil recruitment is a stereotyped response, mainly driven by DAMPs, that does not distinguish the type of threat, whether infectious or non-infectious. In cases of infection, this early response likely occurs to confine the microorganism and limit tissue damage. By contrast, sustained neutrophil recruitment depends on TLRs.

## Results

### Early and late recruitment of neutrophils during microbial infections is regulated by two mechanisms

**Fig. 1:**
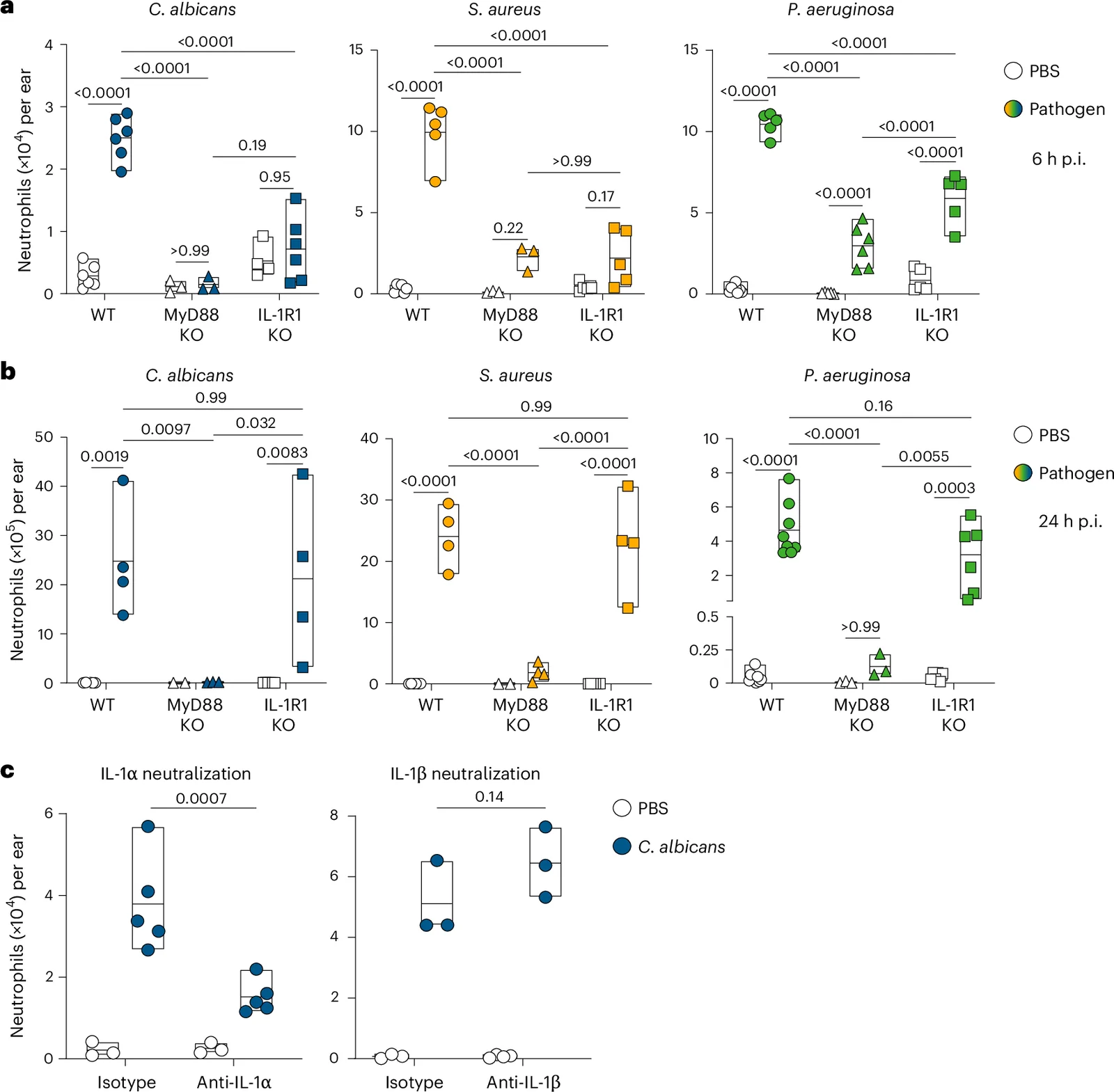
IL-1R signaling is responsible for early neutrophil recruitment during microbial infection. **a**, Neutrophil recruitment at early time points (6 h) after infection with the indicated pathogens in WT, MyD88-KO, or IL-1R1-KO mice. For *C. albicans*: WT (PBS, *n* = 6; pathogen, *n* = 6), MyD88 KO (PBS, *n* = 3; pathogen, *n* = 3) and IL-1R1 KO (PBS, *n* = 4; pathogen, *n* = 6). For *S. aureus*: WT (PBS, *n* = 6; pathogen, *n* = 5), MyD88-KO (PBS, *n* = 3; pathogen, *n* = 3) and IL-1R1 KO (PBS, *n* = 5; pathogen, *n* = 5). For *P. aeruginosa*: WT (PBS, *n* = 8; pathogen, *n* = 5), MyD88 KO (PBS, *n* = 5; pathogen, *n* = 6) and IL-1R1 KO (PBS, *n* = 5; pathogen, *n* = 5). p.i., postinfection. **b**, Neutrophil recruitment at late time points (24 h) after infection with the indicated pathogens in WT, MyD88-KO or IL-1R1 KO mice. For *C. albicans*: WT (PBS, *n* = 6; pathogen, *n* = 4), MyD88 KO (PBS, *n* = 2; pathogen, *n* = 3) and IL-1R1 KO (PBS, *n* = 7; pathogen, *n* = 4). For *S. aureus*: WT (PBS, *n* = 6; pathogen, *n* = 4), MyD88 KO (PBS, *n* = 2; pathogen, *n* = 4) and IL-1R1 KO (PBS, *n* = 6; pathogen, *n* = 4). For *P. aeruginosa*: WT (PBS, *n* = 9; pathogen, *n* = 8), MyD88 KO (PBS, *n* = 3; pathogen, *n* = 3) and IL-1R1 KO (PBS, *n* = 6; pathogen, *n* = 7). **c**, Neutrophil recruitment 6 h after *C. albicans* infection in mice treated with either anti-IL-1α and anti-IL-1β or the respective isotype controls. For IL-1α neutralization: isotype control (PBS, *n* = 3; *C. albicans*, *n* = 5) and anti-IL-1α (PBS, *n* = 3; *C. albicans*, *n* = 5). For IL-1β neutralization: isotype control (PBS, *n* = 3; *C. albicans*, *n* = 3) and anti-IL-1β (PBS, *n* = 4; *C. albicans*, *n* = 3). Data are shown as mean and range (minimum to maximum); *n*, the number of biologically independent samples; each dot represents a mouse. Statistical significance was determined using two-way analysis of variance (ANOVA) followed by (**a**,**b**) Tukey’s or Šidák’s (**c**) multiple comparisons test. *P* values are indicated. Source data

**Extended Data Fig. 1:**
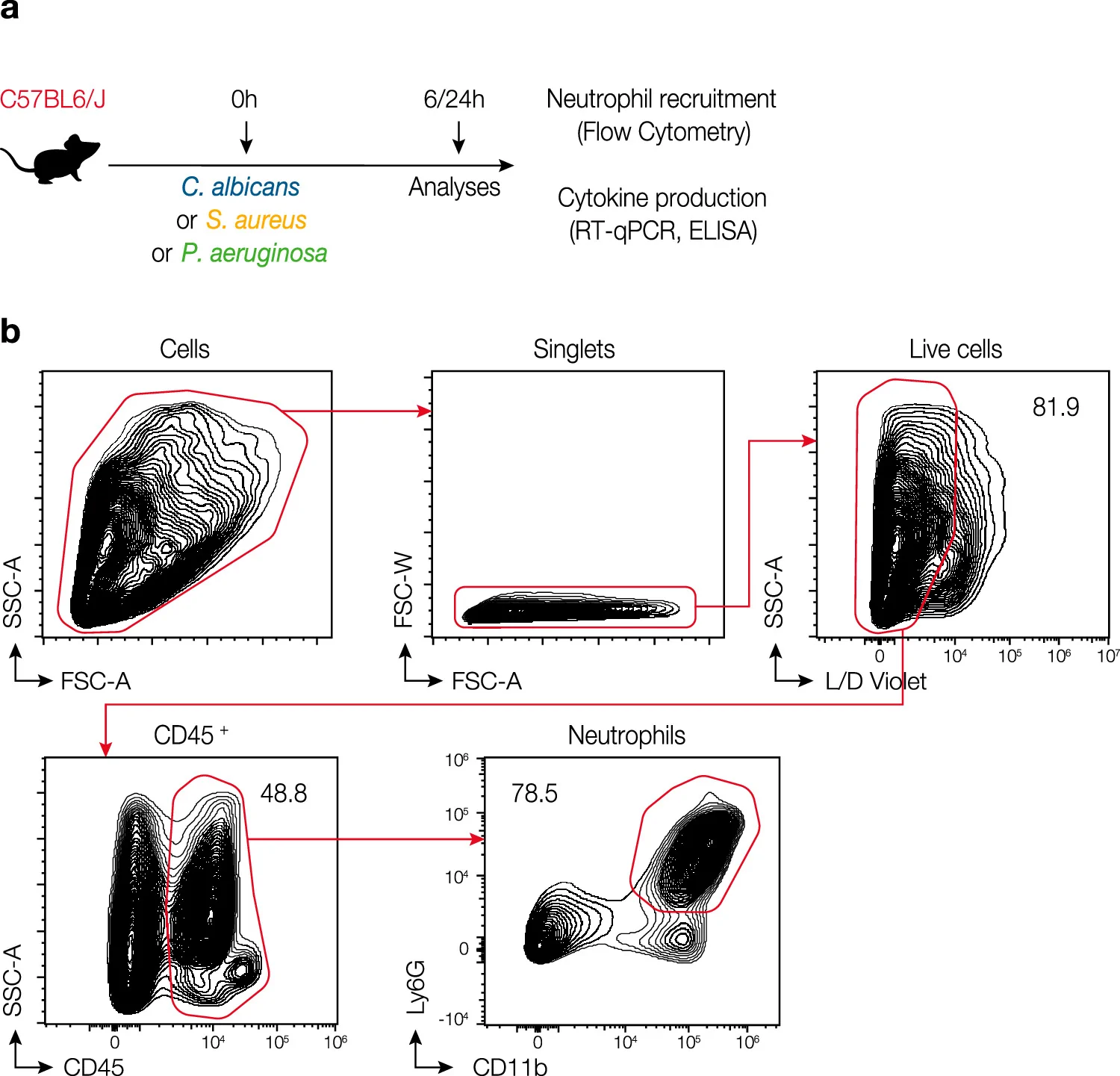
Experimental design and gating strategy for neutrophils identification in mouse skin. **a**) Mice (C57BL/6J, IL-1R1-deficient, or MyD88-deficient) were injected intradermally in the ear with *C. albicans*, *S. aureus*, *P. aeruginosa*, or PBS as control. Neutrophil recruitment and cytokine production were assessed by flow cytometry or RT-qPCR/ELISA, respectively, at two time points (6 and 24 h) post-infection (p.i.). **b**) Representative gating strategy for skin neutrophil identification. After excluding doublets and dead cells (L/D Violet-negative), neutrophils were defined as CD45^+^CD11b^high^Ly6G^high^ cells. The “Rat” icon in panel a is by Uswa KDT, from Noun Project, CC BY 3.0; used without modification.

**Extended Data Fig. 2:**
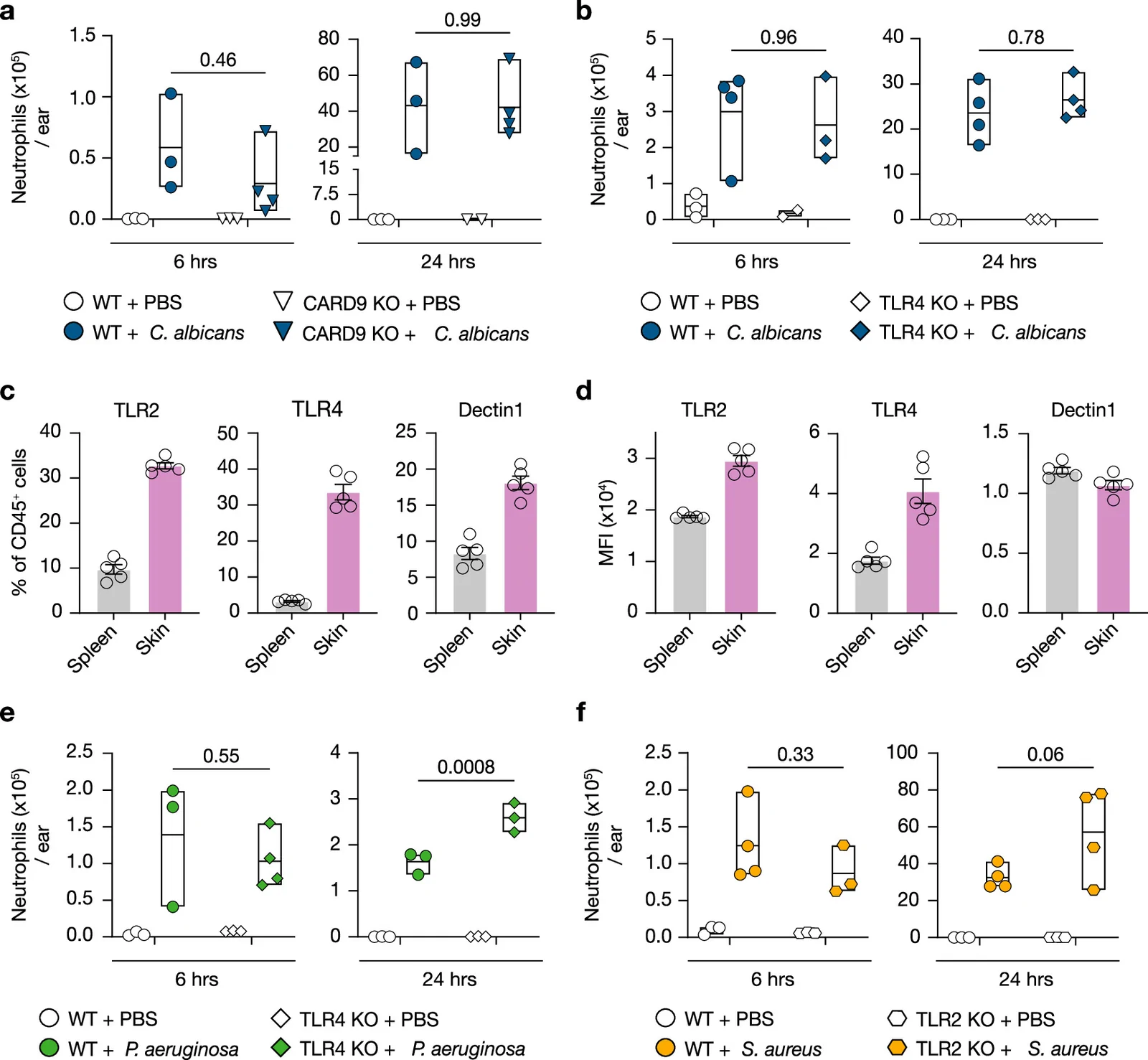
Neutrophil recruitment upon microbial infection is not affected by the absence of CARD9, TLR4 or TLR2. **a**) Neutrophil recruitment at early (6 h) and late (24 h) time points after *C. albicans* infection in C57BL/6J WT and CARD9 KO mice. At 6 h: WT, n = 3 mice per group, and CARD9 KO (PBS, n = 3; pathogen, n = 4). At 24 h: WT, n = 3 mice per group, and CARD9 KO (PBS, n = 2; pathogen, n = 4). **b**) Neutrophil recruitment at early (6 h) and late (24 h) time points after *C. albicans* infection in C57BL/6J WT and TLR4 KO. At 6 h: WT (PBS, n = 3; pathogen, n = 4) and TLR4 KO (PBS, n = 2; pathogen, n = 3). At 24 h: WT and TLR4 KO mice had n = 3 for PBS and n = 4 for pathogen. **c**) Percentage of CD45^+^ cells expressing the indicated PRRs in the skin compared to the spleen (n = 5 mice). **d**) Expression level of the indicated PRRs in CD45^+^ cells in the skin compared to the spleen (n = 5 mice). **e**) Neutrophil recruitment at early (6 h) and late (24 h) time points after *P. aeruginosa* infection in C57BL/6J WT or TLR4 KO mice. At 6 h: WT, n = 3 mice per group, TLR4 KO PBS, n = 3 and pathogen, n = 4. At 24 h: n = 3 mice per group. **f**) Neutrophil recruitment at early (6 h) and late (24 h) time points after *S. aureus* infection in C57BL/6J WT, or TLR2 KO mice. At 6 h: WT, PBS, n = 3 and pathogen, n = 4, whereas TLR2 KO, n = 3 mice per group. At 24 h:, WT and TLR2 KO mice had n = 3 for PBS and n = 4 for pathogen. In a, b, e, f data are shown as mean and range (minimum to maximum), in c and d data are shown as mean ± SD. Each symbol represents one mouse, “n” indicates the number of biologically independent samples. Statistical significance was determined using two-way ANOVA followed by (**a, b**) Tukey’s or Šidák’s (**e, f**) multiple-comparisons. Exact *p* values are indicated. Source data

To investigate the mechanism of neutrophil recruitment following exposure to microorganisms, we utilized a model of skin infection and three distinct types of pathogens, namely a fungus (*C. albicans*), a Gram-positive bacterium (*S. aureus*) and a Gram-negative bacterium (*P. aeruginosa*). These pathogens were injected into the dermis of the ear of wild-type (WT) and MyD88-knockout (KO) mice, the latter lacking IL-1 and TLR signaling pathways, both of which are involved in neutrophil recruitment^21^. We then analyzed neutrophil recruitment at early (6 h) and late (24 h) time points of infection using flow cytometry (Extended Data Fig. 1). We found that the arrival of neutrophils at the site of infection was MyD88-dependent under all tested conditions, including infection with *C. albicans* (Fig. 1a,b). The requirement of MyD88 for neutrophil recruitment upon *C. albicans* challenge was somewhat unexpected. The PAMPs present in the *C. albicans* cell wall are recognized by Dectin-1 and Dectin-2, which signal predominantly through CARD9. Thus, we evaluated neutrophil recruitment in WT and CARD9-KO mice, and found that CARD9 was entirely dispensable (Extended Data Fig. 2a). This aligns with previous work^22^ showing that CARD9 is dispensable for the recruitment of neutrophils in response to *C. albicans* infection in most organs, but not the central nervous system (CNS). The requirement for MyD88 compared with the dispensability of CARD9 for neutrophils to be recruited to the site of infection led us to investigate TLR4, which recognizes O-mannans on the *Candida* cell surface. Similar to the above observations, TLR4-KO mice showed no reduction in the number of neutrophils found in the infected tissue at either early or late time points (Extended Data Fig. 2b).

Considering that MyD88 regulates the signaling of IL-1 and TLRs, we assessed the role of the former by analyzing neutrophil recruitment in mice deficient for IL-1R1. We observed that recruitment was impaired at early, but not late, time points in both bacterial and fungal infections (Fig. 1a,b).

Together, these findings raised the possibility that neutrophils are recruited early after infection through a MyD88-dependent but TLR-independent mechanism, despite the presence of PAMPs and their receptors in the skin (Extended Data Fig. 2c,d). The dispensability of TLR4 for late neutrophil recruitment in mice infected with *C. albicans* and Gram-negative bacteria (Extended Data Fig. 2b,e), as well as the dispensability of TLR2 in *S. aureus* infections (Extended Data Fig. 2f), is consistent with the functional redundancy of the TLR system.

IL-1α and IL-1β are vasoactive cytokines that can stimulate the production of neutrophil chemoattractants, such as CXCL1 and CXCL2, by innate immune cells, stromal cells and keratinocytes^23–25^. Unlike IL-1β, the synthesis of which is inducible, IL-1α is produced constitutively (although it can be upregulated) and is released actively or upon events damaging the integrity of the plasma membrane, such as cell death^26,27^. To determine which of these cytokines initiates the inflammatory process, we locally administered anti-IL-1α and anti-IL-1β to block the activities of these cytokines at the site of infection. Although both cytokines bind to IL-1R1, only the blockade of IL-1α had a significant negative impact on early neutrophil recruitment (Fig. 1c). These findings indicate that IL-1α is the IL-1R1 ligand that mediates the early arrival of neutrophils at the site of infection. This is supported by previous work. Neutrophil recruitment is regulated by IL-1α in Gram-negative bacterial infections caused by *Legionella pneumophila*^28^ and *Yersinia enterocolitica*^29^. Similar results have also been reported in mice infected with *S. aureus*^30^ and *P. aeruginosa*^31^. Overall, these data suggest that one generalized IL-1α-dependent mechanism induces the rapid recruitment of neutrophils upon infection.

### CXCL1 and CXCL2 mediate neutrophil recruitment to the infected tissue with different kinetics

**Fig. 2:**
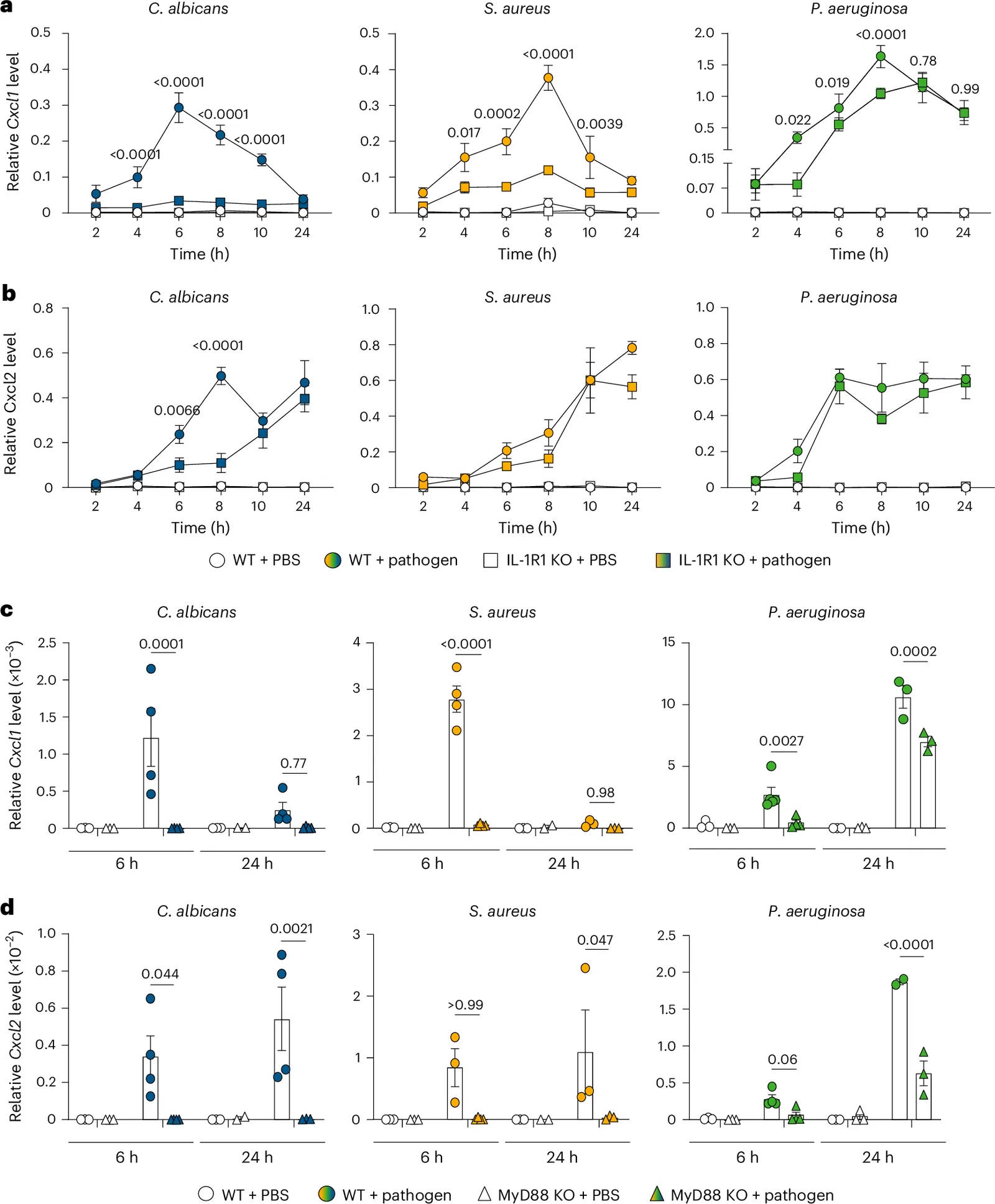
The induction of *Cxcl1* and *Cxcl2* in infected skin follows different kinetics and is regulated by different pathways. **a**, Relative *Cxcl1* expression was quantified in WT and IL-1R1-KO mice infected with the indicated pathogens by quantitative reverse-transcription PCR (RT–qPCR) at different time points. For *C. albicans*, *n* = 6 mice per group at all time points, except for WT infected mice at 4, 6, 8 and 10 h, for which *n* = 5. For *S. aureus*, *n* = 3 mice per group at all time points, except for IL-1R1 KO PBS controls at 8 h, for which *n* = 2. For *P. aeruginosa*, *n* = 3 mice per group at all time points, except for IL-1R1 KO PBS controls at 2, 4 and 6 h and WT PBS controls at 8 and 24 h, for which *n* = 2. **b**, Relative *Cxcl2* expression in WT and IL-1R1 KO mice infected with the indicated pathogens was quantified by RT–qPCR at different time points. For *C. albicans*, *n* = 6 mice per group at all time points, except for WT infected mice at 4, 8, 10 and 24 h and IL-1R1-KO infected mice at 6, 8, 10 and 24 h, for which *n* = 5. For *S. aureus*, *n* = 3 mice per group at all time points, except for IL-1R1-KO PBS controls at 8 h and both infected groups at 24 h, for which *n* = 2. For *P. aeruginosa*, *n* = 3 mice per group at all time points, except for IL-1R1-KO PBS controls at 2, 4, 6 and 10 h, WT PBS controls at 8 and 24 h, and IL-1R1-KO infected mice at 6 h, for which *n* = 2. **c**, Relative *Cxcl1* expression in WT and MyD88-KO mice infected with the indicated pathogens was quantified by RT–qPCR at 6 and 24 h postinfection. For *C. albicans*, each PBS group included 3 mice and each infected group included 4 mice, except for the MyD88-KO PBS control group at 24 h, which included 2 mice. For *S. aureus*, each group included 3 mice, except for the WT and MyD88-KO infected groups at 6 h, which each included 4 mice, and the MyD88-KO PBS control group at 24 h, which included 2 mice. For *P. aeruginosa*, each group included 3 mice, except for the WT and MyD88-KO infected groups at 6 h, which included 5 and 4 mice, respectively. **d**, Relative *Cxcl2* expression in WT and MyD88-KO mice infected with the indicated pathogens was quantified by RT–qPCR at 6 and 24 h postinfection. For *C. albicans*, each group included 3 mice, except for the WT infected groups at 6 and 24 h and the MyD88-KO infected group at 6 h, which each included 4 mice, and the MyD88-KO PBS control group at 24 h, which included 2 mice. For *S. aureus*, each group included 3 mice, except for the MyD88-KO infected group at 6 h, which included 4 mice, and the MyD88-KO PBS control group at 24 h, which included 2 mice. For *P. aeruginosa*, each group included 3 mice, except for the WT and MyD88-KO infected groups at 6 h, which each included 4 mice, and the WT infected group at 24 h, which included 2 mice. Data are presented as mean ± s.d. In **c** and **d**, each symbol represents one biologically independent mouse. *n* indicates the number of biologically independent samples. *Gapdh* (**a**,**b**) and *Rn18s* (**c**,**d**) were used as reference genes. Statistical significance was determined using two-way ANOVA followed by (**a**,**b**) Tukey’s or Šidák’s (**c**,**d**) multiple comparisons test. *P* values are indicated. Source data

To further explore how neutrophils are recruited to the site of infection, we focused on CXCL1 and CXCL2, the two key cytokines in this process. We assessed the expression of *Cxcl1* and *Cxcl2* mRNA in the infected tissue. Both cytokines underwent upregulation in all three infection models, although the kinetics were different. In *C. albicans* and *S. aureus* infections, the mRNA levels of *Cxcl1* progressively increased, peaking at 6–8 h after infection, and then rapidly declined. *Cxcl1* transcription was dependent on IL-1 in both *C. albicans*- and *S. aureus*-infected tissues (Fig. 2a). Although *P. aeruginosa* infections exhibited an early upregulation of *Cxcl1*, mirroring that of the other two microorganisms, this response not only was more marked, but also became IL-1-independent at later time points (Fig. 2a). This is possibly owing to *P. aeruginosa* damaging the skin tissue more than *C. albicans* and *S. aureus*, to an extent that triggers the release of additional inflammatory factors, leading to *Cxcl1* upregulation.

**Extended Data Fig. 3:**
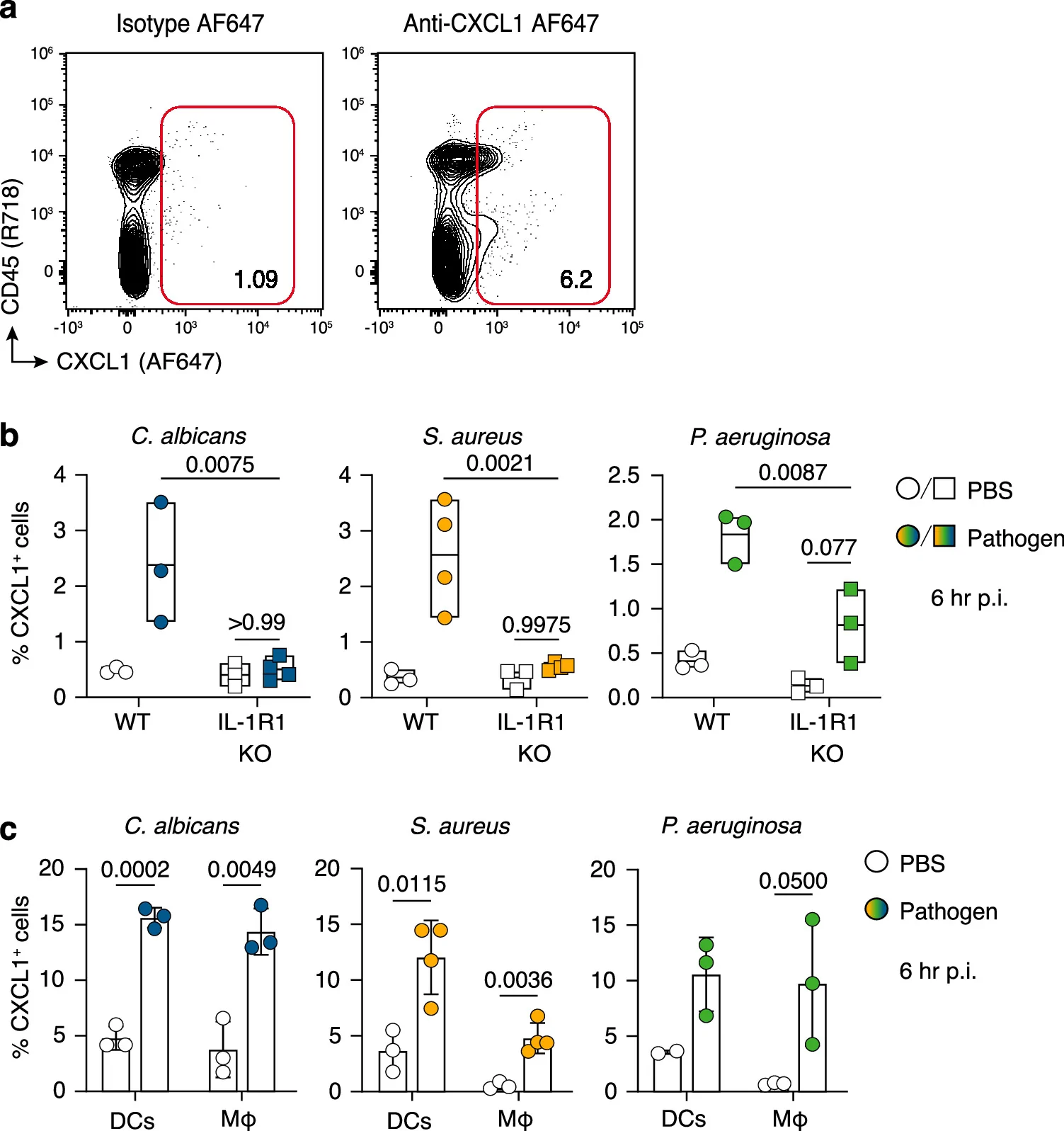
CXCL1 production by DCs and macrophages during microbial infection. **a**) Representative dot plot of CXCL1^+^ cells. **b**) Percentage of CXCL1^+^ cells among CD45^+^ cells at the infection site in WT and IL-1R1 KO mice. For *C. albicans*: WT, n = 3 mice per group; IL-1R1 KO, PBS, n = 3 and pathogen, n = 4 mice. For *S. aureus*: WT and IL-1R1 KO mice had n = 3 for PBS and n = 4 for pathogens. For *P. aeruginosa*: n = 3 mice per group. Data are shown as mean and range (minimum to maximum). **c**) Percentage of CXCL1^+^ DCs and macrophages (Mφ) among total DCs and Mφ, respectively, at 6 h after infection of WT mice with the indicated pathogens. For *C. albicans*: n = 3 mice per group for both cell populations. For *S. aureus*: PBS n = 3 and pathogen n = 4 mice for both cell populations. For *P. aeruginosa*: n = 3 mice per group, except for CXCL1^+^ DCs in the PBS group, for which n = 2. In b, data are shown as mean and range (minimum to maximum); in c data are shown as mean ± SD. Each symbol represents one mouse. “n” indicates the number of biologically independent samples. Statistical significance was determined using two-way ANOVA followed by (**b**) Šidák’s multiple-comparisons or two-tailed unpaired *t*-test (**c**). *p* values are indicated. Source data

Flow cytometry analysis of cells isolated from infected tissue showed that the protein levels of CXCL1 were also increased and were IL-1-dependent (Extended Data Fig. 3a,b).

The critical role of CXCL1 in early neutrophil recruitment during microbial infections prompted us to investigate the source of this chemokine in vivo. Early after infection, flow cytometry analysis showed that CXCL1 production was increased with a notable proportion of CXCL1^+^ cells among DCs and macrophages across all tested infection conditions (Extended Data Fig. 3c).

**Extended Data Fig. 4:**
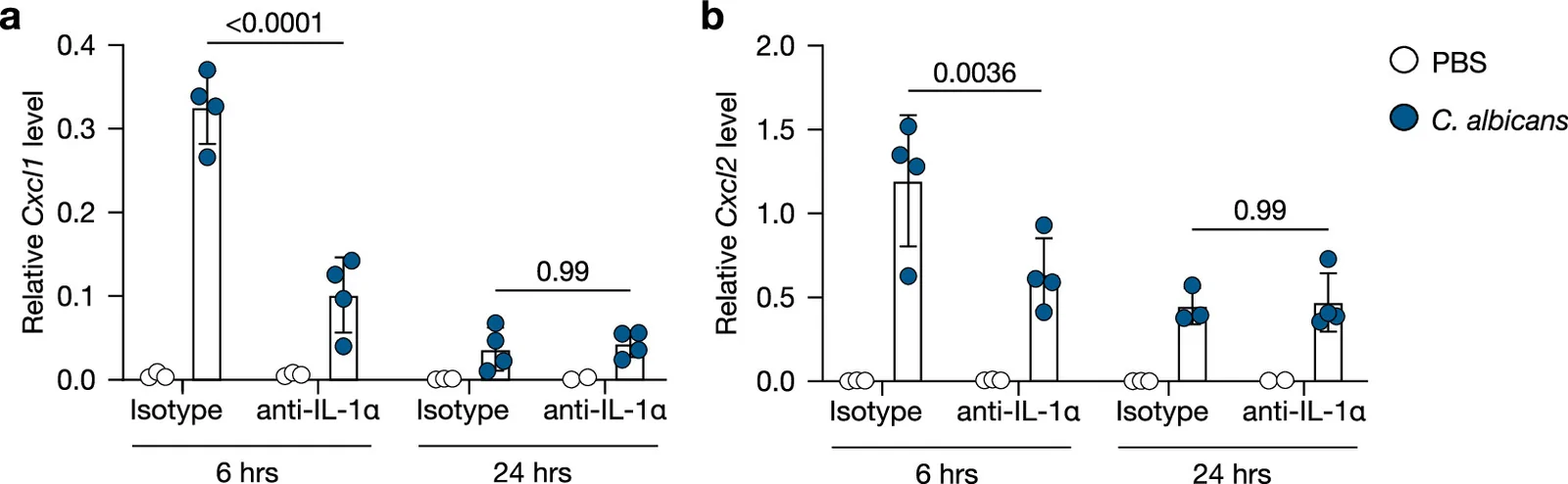
CXCL1 and CXCL2 production with or without IL-1α. **a,b**) *Cxcl1* (**a**) and *Cxcl2*(**b**) expression after IL-1α neutralization during *C. albicans* infection. Gene expression was quantified by RT-qPCR and normalized to *Rn18s*. At both time points, n = 3 mice per PBS group and n = 4 mice per infected group, except for the anti-IL-1α-treated PBS groups at 24 h, for which n = 2, and the isotype-treated infected group in b at 24 h, for which n = 3. Data are shown as mean ± SD. Each symbol represents one mouse. “n” indicates the number of biologically independent samples. Statistical significance was determined using two-way ANOVA followed by Šidák’s multiple-comparisons. *p* values are indicated. Source data

In contrast to CXCL1, CXCL2 exhibited distinct regulation by the three tested microorganisms. *C. albicans* infections showed an early and a late wave of *Cxcl2* upregulation (Fig. 2b), with only the former being IL-1-dependent. The latter wave was not only independent of IL-1, but also coincident with the downregulation of *Cxcl1* (Fig. 2c). Furthermore, although the expression of *Cxcl1* was under the control of IL-1α (Extended Data Fig. 4a), that of *Cxcl2* was regulated by IL-1α only at early time points and was MyD88-dependent at both early and late time points (Fig. 2b,d and Extended Data Fig. 4b). During bacterial infections, the increase in *Cxcl2* levels was delayed compared with the upregulation of *Cxcl1*, and the regulatory mechanism shared both the dependence on MyD88 (Fig. 2d) and the independence from IL-1, similar to what occurs during *C. albicans* infections (Fig. 2b).

**Fig. 3:**
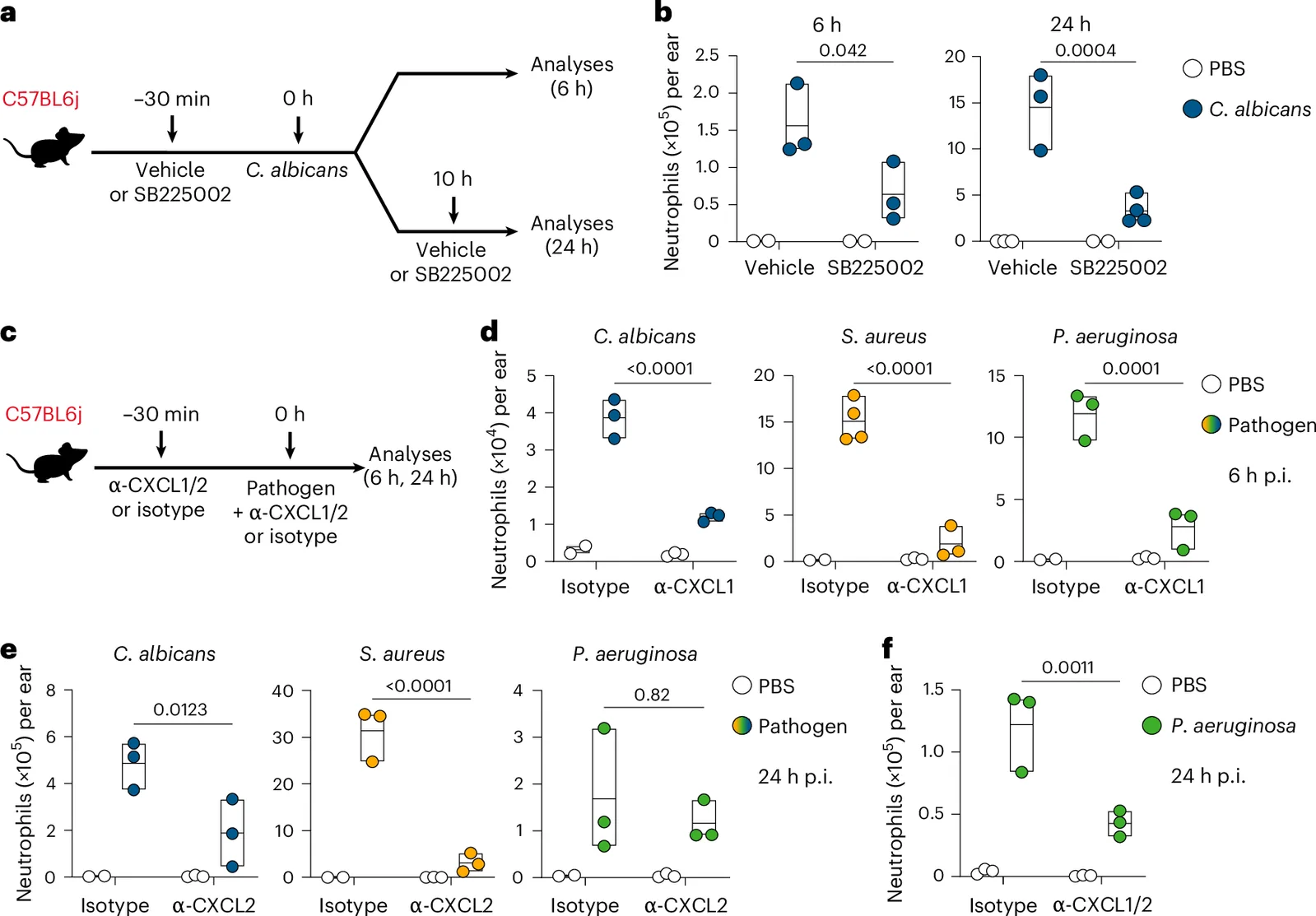
CXCL1 and CXCL2 control neutrophil recruitment. **a**, Schematic of the experimental design. Mice were treated with the CXCR2 inhibitor, SB225002, or vehicle 30 min prior to and 10 h after *C. albicans* infection. Neutrophil recruitment was detected at 6 and 24 h postinfection. **b**, Neutrophil recruitment at 6 and 24 h postinfection. At 6 h: vehicle (PBS, *n* = 2; *C. albicans*, *n* = 3) and SB225002 (PBS, *n* = 2; *C. albicans*, *n* = 3). At 24 h: vehicle (PBS, *n* = 3; *C. albicans*, *n* = 3) and SB225002 (PBS, *n* = 2; *C. albicans*, *n* = 4). **c**, Schematic of the experimental design. Mice were treated with blocking antibodies targeting CXCL1 and CXCL2 (CXCL1/2) or an isotype control 30 min before microbial infection. Neutrophil recruitment was detected at 6 and 24 h postinfection. **d,e**, Neutrophil recruitment at the indicated time points after infection with pathogens in mice treated with either anti-CXCL1 and anti-CXCL2 or the respective isotype controls. **d**, For *C. albicans* and *P. aeruginosa*: isotype control (PBS, *n* = 2; pathogen, *n* = 3) and anti-CXCL1 (PBS, *n* = 3; pathogen, *n* = 3). For *S. aureus*: isotype control (PBS, *n* = 2; pathogen, *n* = 4) and anti-CXCL1 (PBS, *n* = 3; pathogen, *n* = 3). **e**, For all three pathogens: isotype control (PBS, *n* = 2; pathogen, *n* = 3) and anti-CXCL2 (PBS, *n* = 3; pathogen, *n* = 3). **f**, Neutrophil recruitment in mice infected with *P. aeruginosa* after neutralization of CXCL1 and CXCL2. Isotype control (PBS, *n* = 3; *P. aeruginosa*, *n* = 3) and anti-CXCL1/2 (PBS, *n* = 3; *P. aeruginosa*, *n* = 3). Data are shown as mean and range (minimum to maximum). *n* indicates the number of biologically independent samples. Statistical significance was determined using two-way ANOVA followed by Šidák’s multiple comparisons test. Exact *P* values are indicated. Credit: rat icon in **a** and **c**, Noun Project. Source data

Given the distinct kinetics and mechanisms of CXCL1 and CXCL2 production, we examined their roles in neutrophil recruitment (Fig. 3a). We first blocked their cognate receptor, CXCR2, using a pharmacological inhibitor (Fig. 3a) and thereby corroborated that this signaling pathway was indeed the main player driving the arrival of neutrophils at the site of infection in our system (Fig. 3b).

Next, we utilized blocking antibodies to individually inhibit these chemokines in vivo (Fig. 3c). Blocking CXCL1 at early time points and CXCL2 at late time points after *C. albicans* or *S. aureus* infection had a major impact on neutrophil recruitment (Fig. 3d,e). CXCL1 was equally important for neutrophil recruitment at early time points during *P. aeruginosa* infection and during infection with the other micro-organisms (Fig. 3d). At later stages of *P. aeruginosa* infection, however, both CXCL1 and CXCL2 were necessary for neutrophil recruitment to the infection site (Fig. 3e,f). It is noteworthy that CXCL1 production at very early time points after *P. aeruginosa* infection is dependent on IL-1α but becomes IL-1-independent and MyD88-dependent at later stages (Fig. 2a,c), similar to CXCL2 production (Fig. 2b,d).

Taken together, these results indicate that (1) CXCL1 and CXCL2 play a major role in the recruitment of neutrophils at the early and late stages of infection, respectively, and (2) the upregulation of CXCL1 and CXCL2 requires MyD88, although (3) CXCL1 production is regulated by an IL-1-dependent mechanism early after infection, whereas (4) CXCL2 production is IL-1-independent during bacterial infections, as well as at late stages of *C. albicans* infection. Therefore, with MyD88 acting downstream of both IL-1 and TLRs, the recruitment of neutrophils is MyD88- and IL-1α-dependent at early stages after infection, and is MyD88-dependent but IL-1α-independent at late stages after infection. This suggests that TLRs come into play only long after infection has begun.

### The LTB_4_–IL-1–CXCL1 axis promotes neutrophil recruitment early postinfection

On the basis of the above results, it appears that the initial surge of neutrophil recruitment at infection sites is mainly danger-driven and controlled by the constitutively expressed alarmin IL-1α. However, we also found that the process becomes predominantly TLR-dependent at later stages. Because the late wave of neutrophil recruitment follows the well-established PRR-dependent mechanism, previous studies presumably lacked the temporal resolution to investigate the early phase.

Therefore, we sought to characterize the first wave of neutrophil recruitment to infected tissue in greater detail. We hypothesized that, if it were independent of the insult, it could utilize mechanisms resembling those of sterile inflammation, including the LTB_4_–IL-1–CXCL1 axis^17^, despite the presence of large amounts of PAMPs and high PRR expression in the skin (Extended Data Fig. 2c,d).

**Fig. 4:**
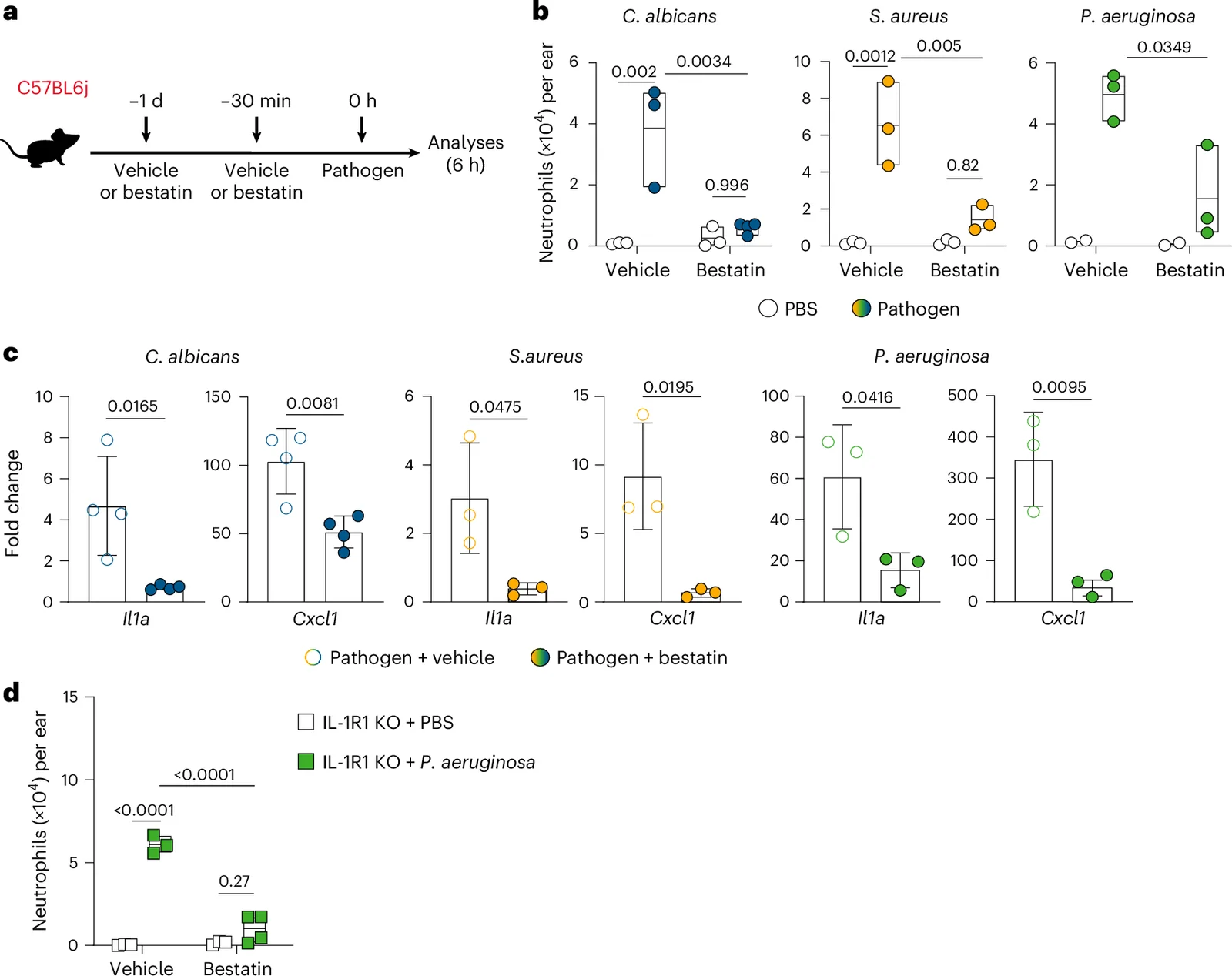
LTB_4_ regulates neutrophil recruitment and *Il1a* and *Cxcl1* expression. **a**, Schematic of the experimental design. Mice were treated with bestatin 1 day and 30 min before microbial infection and analyzed 6 h later. **b**, Neutrophil recruitment 6 h postinfection with the indicated pathogens in mice treated with LTB4 inhibition (bestatin) or vehicle. For *C. albicans* and *S. aureus*, *n* = 3 mice per group except for *C. albicans* + bestatin, for which *n* = 4. For *P. aeruginosa*, vehicle (PBS, *n* = 2; pathogen, *n* = 3) and bestatin (PBS, *n* = 2; pathogen, *n* = 3). **c**, Relative *Il1a* and *Cxcl1* expression in mice treated with bestatin or vehicle and infected with the indicated pathogens quantified by RT–qPCR at 6 h postinfection. Fold change values were calculated by normalizing the expression level of each sample to the mean expression of the reference group. The expression level of each cytokine was calculated using *Rn18s* as a reference gene. For *C. albicans*, *n* = 4 mice per group; for *S. aureus* and *P. aeruginosa*, *n* = 3 mice per group. **d**, Neutrophil recruitment in IL-1R1-KO mice treated with the LTB_4_ inhibitor, bestatin, or vehicle at 6 h after *P. aeruginosa* infection. *n* = 3 mice per group, except for the bestatin-treated, *P. aeruginosa*-infected group, for which *n* = 4 mice. *n* indicates the number of biologically independent samples. In **b** and **d**, data are shown as mean and range (minimum to maximum). In **c**, bars show the mean ± s.d. Statistical significance was determined using two-way ANOVA followed by Šidák’s multiple comparisons test (**b**,**d**) and a two-tailed unpaired *t*-test (**c**). *P* values are indicated in the graphs. Credit: rat icon in **a**, Noun Project. Source data

**Extended Data Fig. 5:**
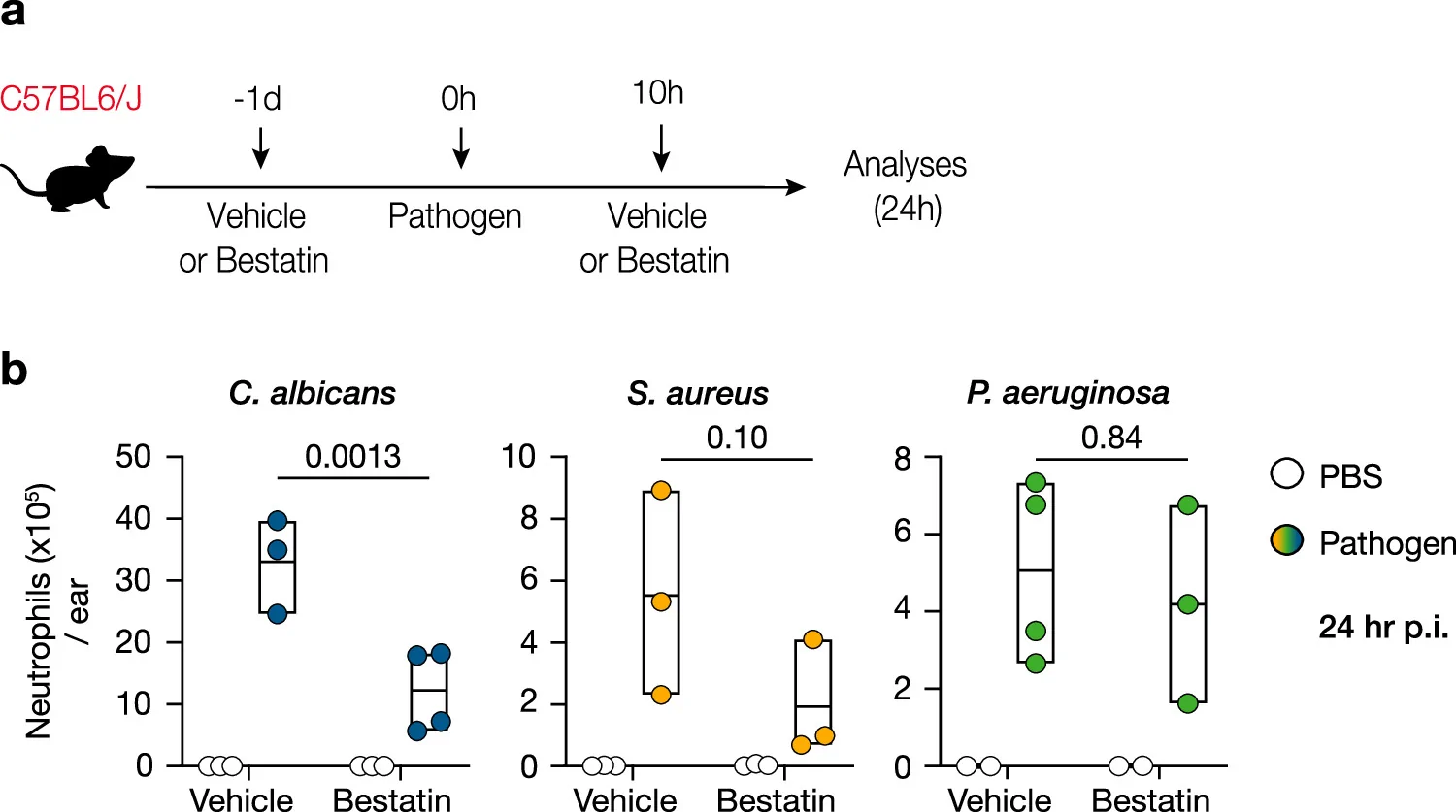
Neutrophil recruitment with or without LTB_4_ during microbial infection. **a**) Experimental design. WT mice were injected i.p. with Bestatin or vehicle at day −1, at the time of infection (day 0), and 10 h post-infection. Neutrophils at the infection site were quantified 24 h later. **b**) Number of neutrophils at the infection site 24 h p.i. with the indicated pathogens. For *S. aureus*, n = 3 mice per group. For *C. albicans*: vehicle, n = 3; pathogen n = 3, Bestatin+vehicle, n = 3; Bestatin+pathogen, n = 4. For *P. aeruginosa*: vehicle, vehicle+Bestatin, n = 2; pathogen, n = 4, and Bestatin+pathogen n = 3. Data are shown as mean and range (minimum to maximum). Each symbol represents one mouse; “n” indicates the number of biologically independent samples. Statistical significance was determined using two-way ANOVA followed by Šidák’s multiple-comparisons. *p* values are indicated. The “Rat” icon in panel a is by Uswa KDT, from Noun Project, CC BY 3.0; used without modification. Source data

To gain insight into the possible involvement of this signaling axis, we used bestatin to inhibit LTA4 hydrolase (Fig. 4a), the enzyme that converts LTA4 into LTB_4_. We inhibited LTB_4_ synthesis and measured the effects on neutrophil recruitment and the upregulation of IL-1α and CXCL1. We found that LTB_4_ inhibition strongly reduced neutrophil recruitment at early time points after infection (Fig. 4a,b) and had a much smaller impact at later stages (Extended Data Fig. 5). These effects were consistently observed with all three types of microorganisms (Fig. 4b and Extended Data Fig. 5b).

The finding that LTB_4_ is critical for the migration of neutrophils into the skin at early time points after infection prompted us to investigate whether IL-1α could indeed be a downstream target of LTB_4_. To test this, we examined the impact of LTB_4_ inhibition on the upregulation of IL-1α and CXCL1 in infected tissues. Treating mice with bestatin prior to infection with each of the three microorganisms effectively prevented the early upregulation of IL-1α and CXCL1 (Fig. 4c).

This and the fact that *Il1a* is necessary for the upregulation of *Cxcl1* (Fig. 2) jointly suggest that the LTB_4_–IL-1α–CXCL1 axis is crucial for neutrophil recruitment during the early stages of microbial infections.

Although the inhibition of LTB_4_ completely blocked the recruitment of neutrophils during infections with *C. albicans* and *S. aureus*, residual recruitment was observed during infection with *P. aeruginosa* (Fig. 4b). Therefore, we examined whether LTB_4_ and IL-1α were solely responsible for the early recruitment of neutrophils. Even for *P. aeruginosa* infection, during which the release of other danger signals could occur^32^, the simultaneous blockade of LTB_4_ and IL-1α was sufficient to fully prevent neutrophil infiltration at the infection site (Fig. 4d).

These data indicate not only that tissue danger signals are required to initiate inflammation during infections, but also that these signals are IL-1α and LTB_4_, as in sterile inflammation.

### The mechanoreceptor Piezo1 regulates LTB_4_ production for the early recruitment of neutrophils to infected tissue

**Fig. 5:**
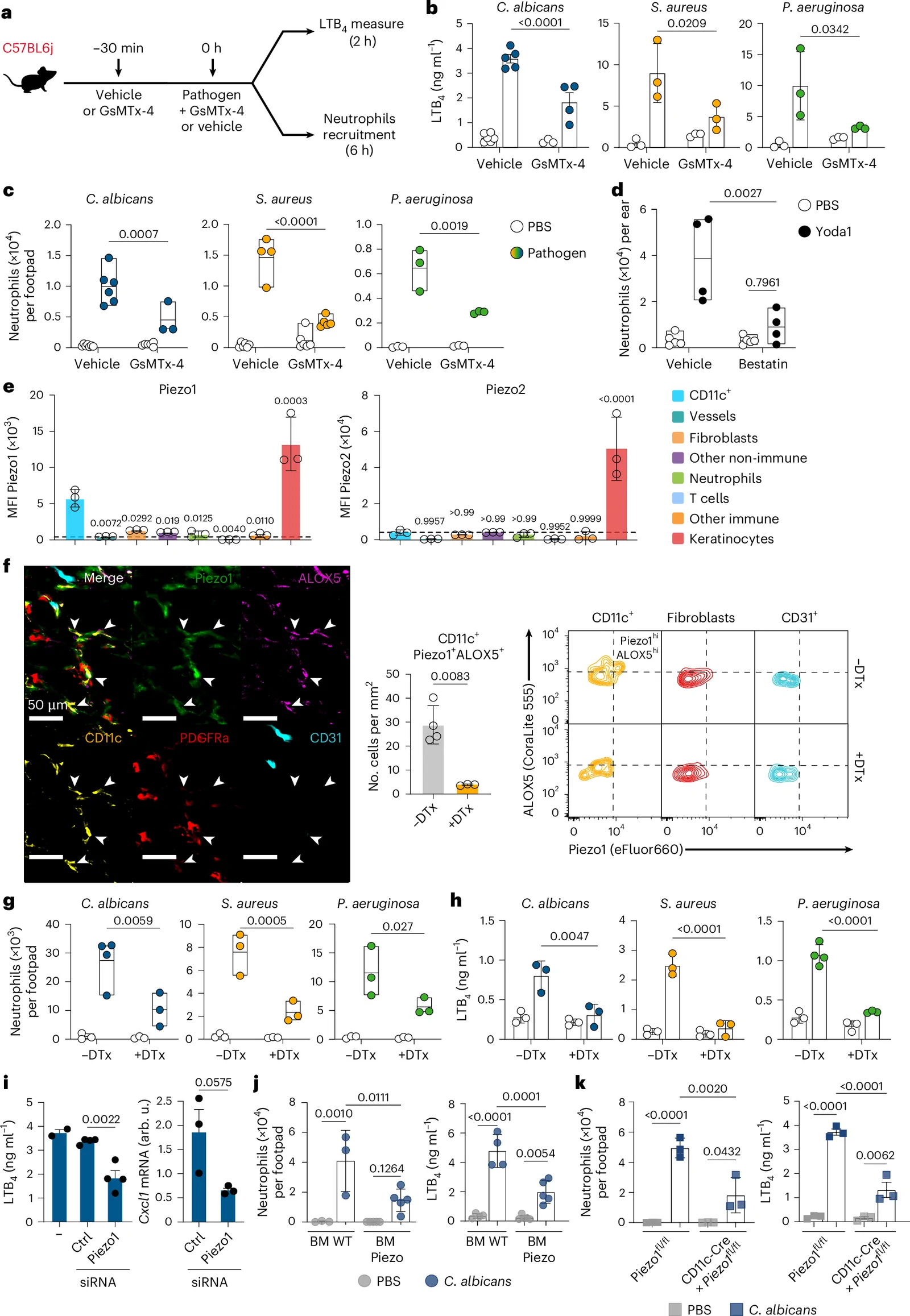
Piezo1 mediates early neutrophil recruitment through the release of LTB_4_ acting with IL-1. **a**, Schematic of the experimental design. Mice were treated with the Piezo inhibitor GsMTx-4 or vehicle 30 min before pathogen infection, followed by a second treatment with the inhibitor at the time of infection. LTB_4_ levels and neutrophil recruitment were assessed 2 and 6 h later, respectively. **b**, LTB_4_ levels in infected mice treated with the Piezo inhibitor, GsMTx-4 or vehicle. LTB_4_ was measured 2 h after infection. For *C. albicans*: vehicle (PBS, *n* = 6; pathogen, *n* = 5) and GsMTx-4 (PBS, *n* = 3; pathogen, *n* = 4). For *S. aureus* and *P. aeruginosa*, *n* = 3 mice per group. **c**, Neutrophil recruitment at 6 h after infection with the indicated pathogens in mice treated with the Piezo inhibitor, GsMTx-4 or vehicle. For *C. albicans*: vehicle (PBS, *n* = 7; pathogen, *n* = 6) and GsMTx-4 (PBS, *n* = 6; pathogen, *n* = 3). For *S. aureus*: vehicle (PBS, *n* = 6; pathogen, *n* = 4) and GsMTx-4 (PBS, *n* = 6; pathogen, *n* = 5). For *P. aeruginosa*, *n* = 3 mice per group. **d**, Neutrophil recruitment measured 6 h after treatment with the Piezo activator Yoda1, in the presence or absence of bestatin administered 1 day and 20 min before Yoda1 was administered. For both vehicle- and bestatin-treated mice, PBS, *n* = 5, and Yoda1, *n* = 4. **e**, FACS analysis of Piezo1 and Piezo2 expression in the indicated skin cells; data represent median fluorescence intensity (MFI). *n* = 3 mice per cell population. **f**, Left, representative multiplexed immunofluorescence images of dermal tissue showing Piezo1 (green) and Alox5 (pink) expression together with CD11c (yellow), PDGFRα (red) and CD31 (blue). White arrows indicate regions of the field containing Piezo1^+^Alox5^+^ double-positive cells. Scale bars, 50 μm. Middle, quantification of the number of Piezo1^+^Alox5^+^CD11c^+^ cells per mm^2^ before and after DTx administration (−DTx, *n* = 4; +DTx, *n* = 3). Right, contour plots of immunofluorescence analysis gated on Piezo1^+^Alox5^+^ double-positive dermal cells, showing their distribution within CD11c^+^ cells, fibroblasts (PDGFRα^+^) and endothelial cells (CD31^+^). Note that only CD11c^+^ cells contain a distinct Piezo1^hi^Alox5^hi^ population, which completely disappeared after DTx administration. Distributions are calculated on the same number of cells; when DTx is administered, plots refer to the remaining CD11c^+^ cells. Data are representative of 3 independent experiments. **g**, Neutrophil recruitment 6 h postinfection in DOG mice that were or were not treated with DTx to eliminate CD11c^+^ cells. For *C. albicans*: −DTx (PBS, *n* = 3; pathogen, *n* = 4) and +DTx (PBS, *n* = 3; pathogen, *n* = 3). For *S. aureus* and *P. aeruginosa*, *n* = 3 mice per group. **h**, LTB_4_ levels in DOG mice that were or were not treated with DTx 2 h postinfection. For *C. albicans* and *S. aureus*, *n* = 3 mice per group. For *P. aeruginosa*: −DTx (PBS, *n* = 3; pathogen, *n* = 4) and +DTx (PBS, *n* = 3; pathogen, *n* = 3). **i**, Left, LTB_4_ levels measured in Piezo1-knockdown or control mice 2 h after *C. albicans* infection (uninfected, *n* = 2; control siRNA, *n* = 4; si*Piezo1*, *n* = 4). Right, relative *Cxcl1* expression in Piezo1-knockdown or control mice 6 h after *C. albicans* infection, quantified by RT–qPCR. *n* = 3 per group. *Gapdh* was used as a reference housekeeping gene. A.U., arbitrary units. **j**, Neutrophil recruitment (6 h) and LTB_4_ production (2 h) following *C. albicans* infection of bone marrow chimeric mice with local deletion of *Piezo1* or of control chimeric mice. For neutrophil recruitment: bone marrow (BM) WT, *n* = 3 mice per group, and BM Piezo, *n* = 5 mice per group. For LTB4 levels measured 2 h after infection: BM WT, *n* = 4 mice per group, and BM Piezo, *n* = 5 mice per group. **k**, Neutrophil recruitment (6 h) and LTB_4_ production (2 h) following *C. albicans* infection of CD11c-CRE × *Piezo1*^fl/fl^ mice and control (*Piezo1*^fl/fl^) littermates. *n* = 3 per group for both analyses. In **b**, **e** and f (middle) and **h**–**k**, bars show the mean ± s.d. In **c**, **d** and **g**, data are shown as mean and range (minimum to maximum). Each symbol represents one biologically independent mouse. *n* indicates the number of biologically independent samples. Statistical significance was determined using two-tailed unpaired *t*-test (**f**, **i** right), one-way ANOVA followed by Tukey’s multiple comparisons (**e**, **i** left) and two-way ANOVA followed by Tukey’s (**b**, **d**, **j**, **k**) or Šidák’s (**c**, **g**, **h**) multiple comparisons test. Exact *P* values are indicated. Credit: rat icon in **a**, Noun Project. Source data

**Extended Data Fig. 6:**
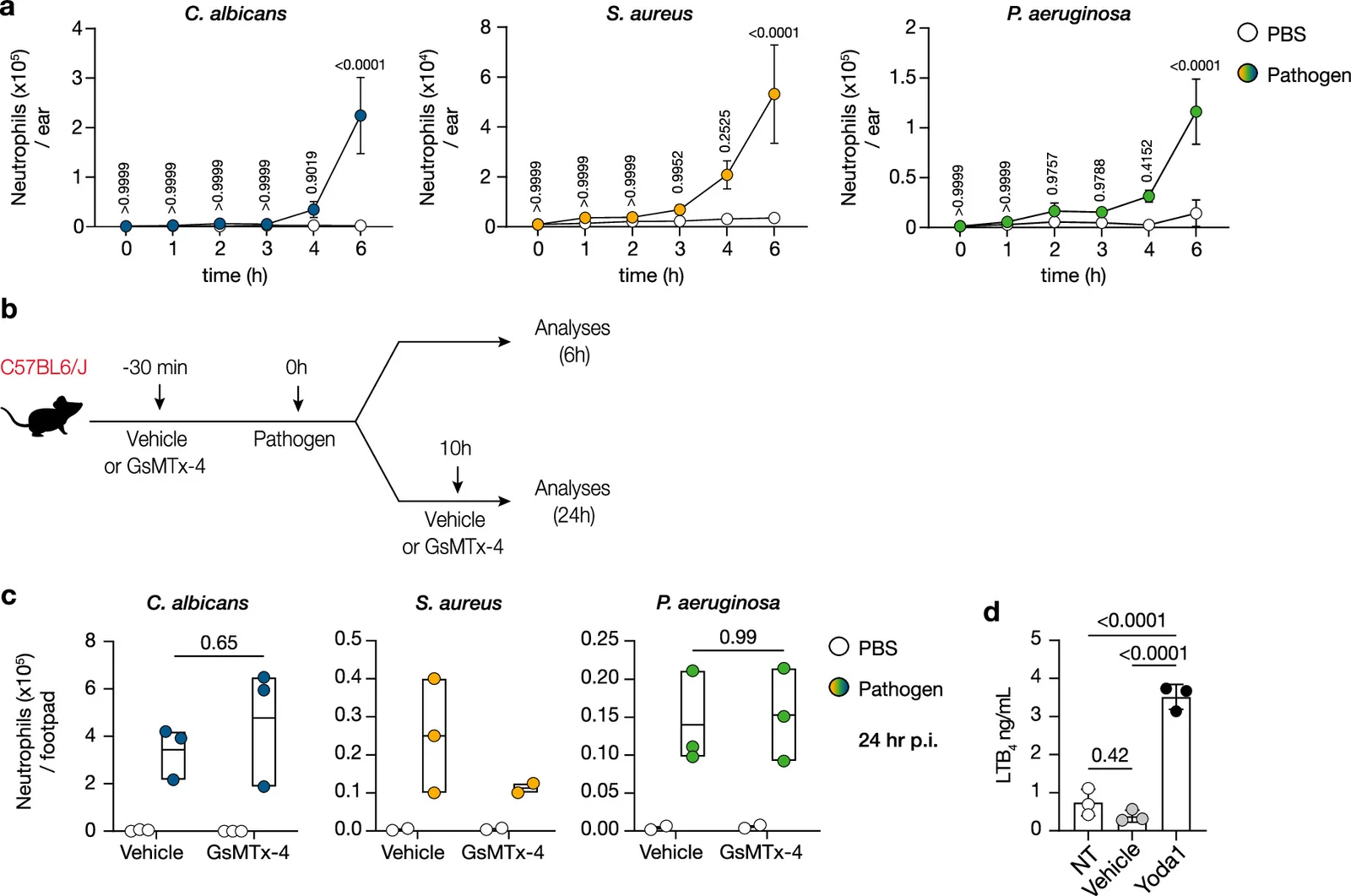
Neutrophil recruitment with or without mechanosensing during microbial infection. **a**) Kinetics of neutrophil accumulation in the skin upon infection with the indicated pathogens. For *C. albicans*, n = 3 mice per group at each time point. For *S. aureus*, n = 3 mice per group at each time point. For *P. aeruginosa*, n = 3 mice per group at each time point, except at 0 h, for which n = 4 mice per group, and for the PBS group at 4 h, for which n = 2. Data are presented as mean ± SD. **b**) Experimental design. Mice were treated with the Piezo1 channel inhibitor GsMTx-4 or vehicle 30 min before pathogen injection and again 10 h post-infection. Neutrophils at the infection site were quantified 24 h after infection. **c**) Number of neutrophils at the infection site 24 h p.i. with the indicated pathogens. For *C. albicans*, n = 3 mice per group. For *S. aureus*: vehicle (PBS, n = 2; pathogen, n = 3) and GsMTx-4 (PBS, n = 2; pathogen, n = 2). For *P. aeruginosa*, both vehicle- and GsMTx-4-treated mice had n = 2 for PBS and n = 3 for pathogen. Each dot represents a mouse. Data are shown as as mean and range (minimum to maximum). **d**) LTB_4_ production by D1 cells stimulated with the Piezo agonist Yoda1, 40 μM, or DMSO as control (NT, not treated) (n = 3, each dot is the mean of a technical replicate). Data are shown as mean ± SD. “n” indicates the number of biologically independent samples. Statistical significance was determined using two-way ANOVA followed by Šidák’s multiple-comparisons (**a**) or Tukey’s multiple-comparisons (**c**) and one-way ANOVA followed by Šidák’s multiple-comparisons (**d**). *p* values are indicated. The “Rat” icon in panel b is by Uswa KDT, from Noun Project, CC BY 3.0; used without modification. Source data

It is known that Ca^2+^ activates the pathway mediating LTB_4_ production^33^ and that mechanoreceptors, such as Piezo1, mobilize Ca^2+^ upon activation^6^. We thus assessed the role of Piezo channels in the production of LTB_4_ as an early response to microorganisms, using the toxin GsMTx-4 (ref. ^34^) to block them, and measuring the number of neutrophils in mice infected with the three pathogens (Fig. 5a). As neutrophils can contribute to the overall LTB_4_ production^16^, we determined when neutrophil accumulation at the site of infection can first be detected. Time-course experiments showed that the number of tissue neutrophils started to significantly increase after 4 h postinfection, independently of the pathogen employed (Extended Data Fig. 6a). On the basis of these findings, LTB_4_ production was assessed at 2 h postinfection to avoid the contribution of neutrophils. LTB_4_ levels were significantly reduced when the Piezo mechanoreceptor could not be activated (Fig. 5b). Piezo inhibition also significantly reduced the number of recruited neutrophils at early (Fig. 5c) but not late time points (Extended Data Fig. 6b,c). Conversely, dermal injection of the Piezo1 activator Yoda1 (ref. ^35^) was sufficient to induce neutrophil recruitment, and this response required LTB_4_ production (Fig. 5d). In accordance, LTB_4_ production was induced by Yoda1 in the mouse DC line D1 (ref. ^36^) (Extended Data Fig. 6d).

**Extended Data Fig. 7:**
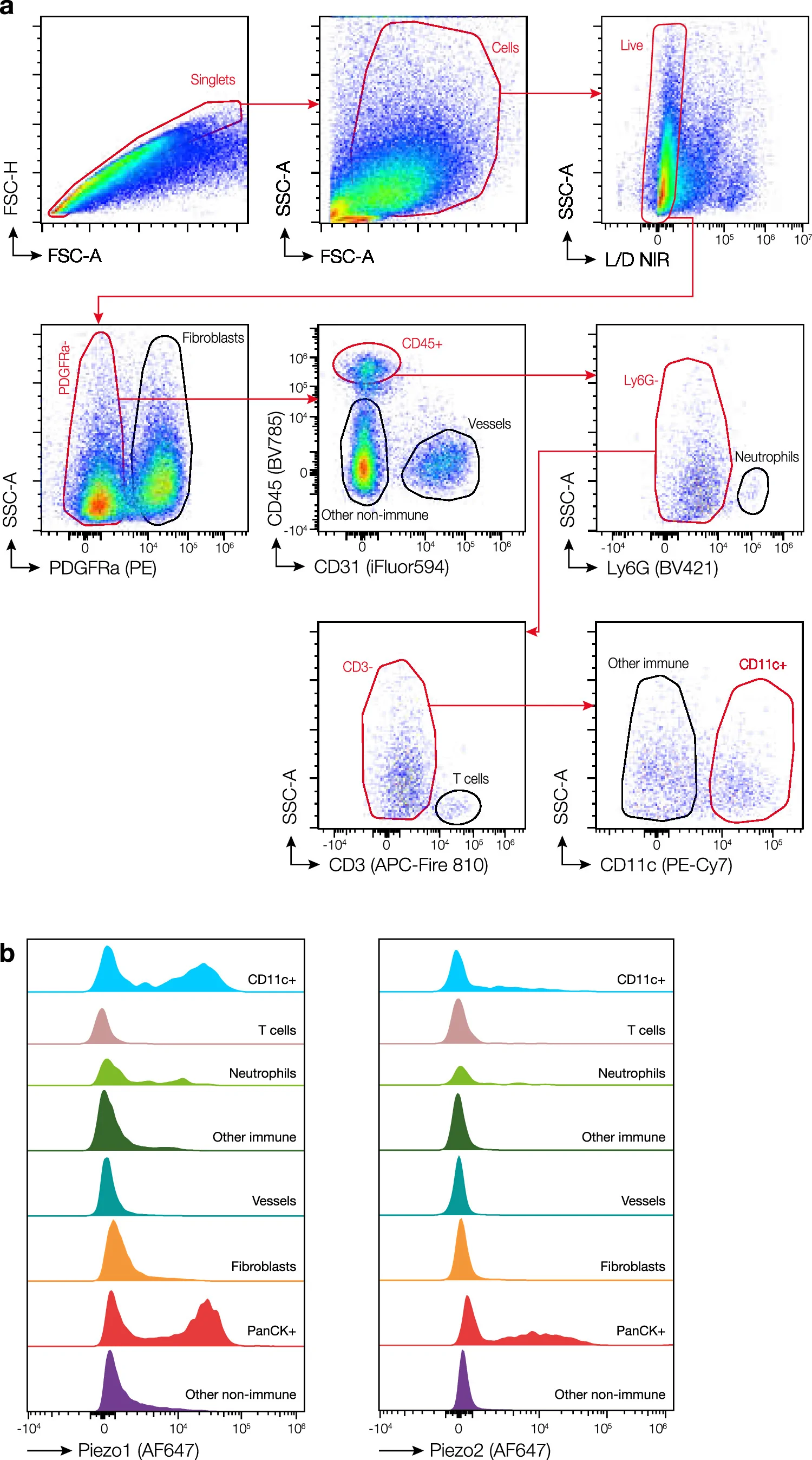
Flow cytometry gating strategy and Piezo1/Piezo2 expression in skin cell populations. **a**) Representative flow cytometry gating strategy used to identify major immune and stromal cell populations in skin samples. Singlets were first selected based on FSC**-**A and FSC**-**H, followed by exclusion of dead cells using live/dead staining. CD45^+^ immune cells, CD45^**−**^ stromal cells, CD31^+^ vessels and PDGFRα^+^ fibroblasts were identified. Within the CD45^+^ compartment, neutrophils were gated as Ly6G^+^ cells, T cells as CD3^+^ cells, and CD11c^+^ cells were distinguished from other immune cells. **b**) Representative histograms showing Piezo1 and Piezo2 staining in the indicated skin cell populations, including CD11c^+^ cells, T cells, neutrophils, other CD45^+^ immune cells, vessels, PDGFRα^+^ fibroblasts, epithelial cells and other CD45^−^ non-immune cells. Histograms show relative fluorescence intensity for Piezo1 or Piezo2 in each gated population.

**Extended Data Fig. 8:**
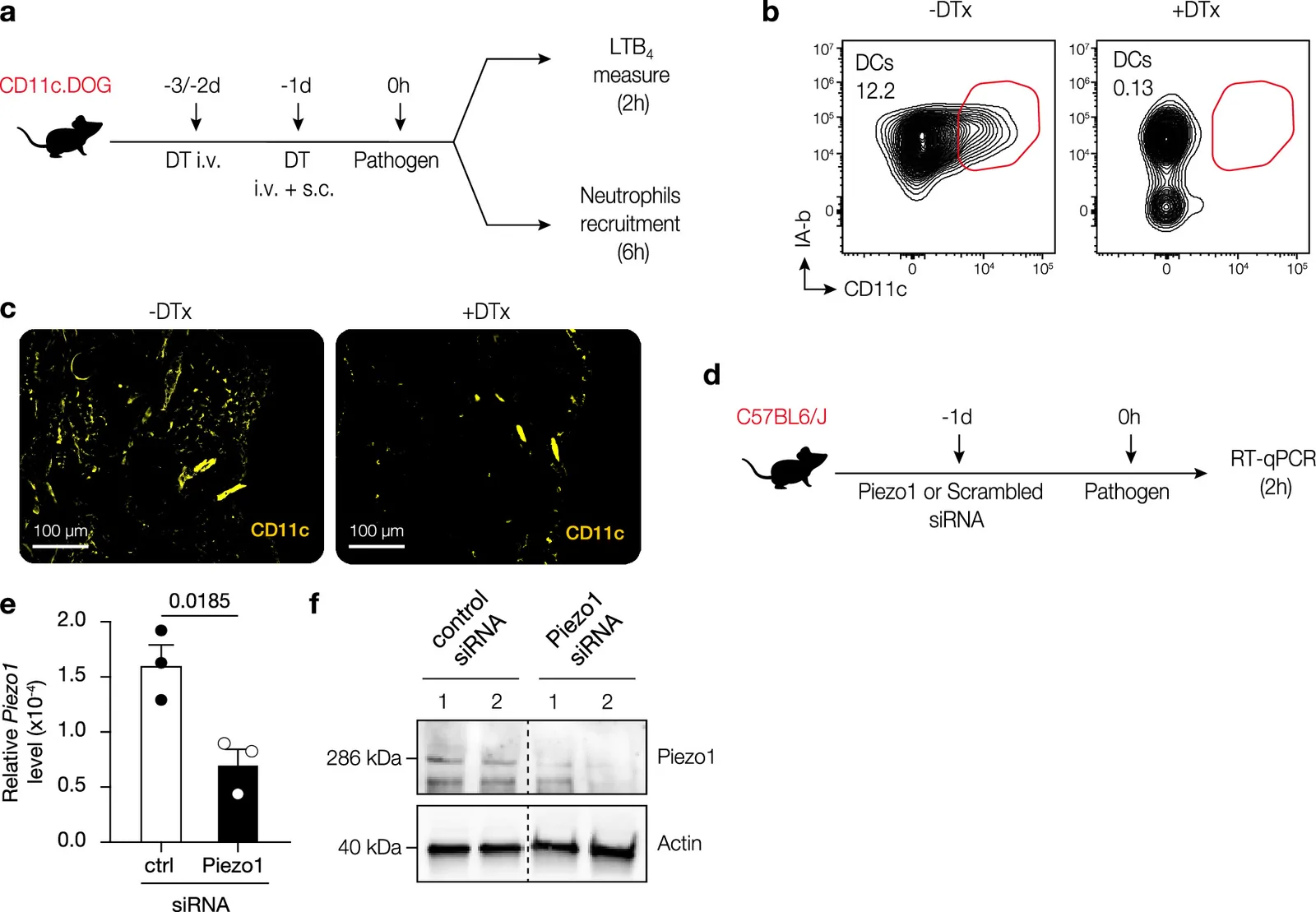
Controls for DC ablation and Piezo1 downregulation. **a**) Experimental design. CD11c.DOG mice were injected with diphtheria toxin (DTx) i.v. at day −3/ − 2 and i.v. and s.c. at day −1 prior to infection. At day 0, mice were infected with pathogens and neutrophils counted at the infection site 6 h later. DC depletion was analysed by flow cytometry. **b**) Representative flow cytometry plots showing the percentage of DCs before and after DTx treatment. **c**) Multiplexed immunofluorescence images showing CD11c^+^ cells before and after DTx administration. **d**) Experimental design for *Piezo1* silencing *in vivo*. Mice were injected intradermally in the footpad with *Piezo1* or scrambled siRNA one day prior to infection. **e,f**) *Piezo1* expression in the footpad measured one day post siRNA injection. **e**) RT-qPCR (normalized to *Rn18s*) **f**) Western Blot of footpad lysates (n = 2 mice). Data are shown as mean ± SD (n = 3 mice), *p* values are indicated. “n” indicates the number of biologically independent samples. Statistical significance was determined using two-tailed unpaired *t*-test. *p* values are indicated. The “Rat” icon in panels a and d is by Uswa KDT, from Noun Project, CC BY 3.0; used without modification. Source data

We then investigated the expression pattern of Piezo1 and Piezo2 in the skin. Cytofluorimetric analysis showed that Piezo1 is expressed at high levels in CD11c^+^ cells and keratinocytes and moderately in the endothelium. Piezo2 was restricted to keratinocytes (Fig. 5e and Extended Data Fig. 7). Therefore, in the dermis, the tissue layer that constitutes the site of infection, only Piezo1 was expressed and was primarily expressed by CD11c^+^ cells (Fig. 5e). Next, we examined which dermal cell types express Piezo1 and Alox5, a key enzyme required for LTB_4_ biosynthesis. This analysis revealed that CD11c^+^ cells frequently express Piezo1 and Alox5 (Fig. 5f left) and that CD11c^+^ cells contain a distinctive Piezo1^hi^Alox5^hi^ population (Fig. 5f right). The deletion of CD11c^+^ cells in CD11c.DOG mice^37^ (DOG mice) upon diphtheria toxin (DTx) administration (Extended Data Fig. 8a–c) strongly decreased both neutrophil recruitment (Fig. 5g) and the production of LTB_4_ (Fig. 5h) upon infection with the three pathogens. DTx eliminated most Piezo1^+^Alox5^+^CD11c^+^ cells (Fig. 5f middle), and the remaining CD11c^+^ population did not contain Piezo1^hi^Alox5^hi^ cells (Fig. 5f). Overall, these data suggest that CD11c^+^ cells, through Piezo1-dependent sensing of microbial signals, are key initiators of LTB_4_-mediated neutrophil recruitment during early immune responses in the skin. Hence, we used small interfering RNAs (siRNAs) to reduce the expression of the *Piezo1* channel in the dermis (Extended Data Fig. 8d–f). *Piezo1* silencing phenocopied the results obtained with GsMTx-4 and also reduced *Cxcl1* levels in mice infected with *C. albicans* (Fig. 5i).

**Extended Data Fig. 9:**
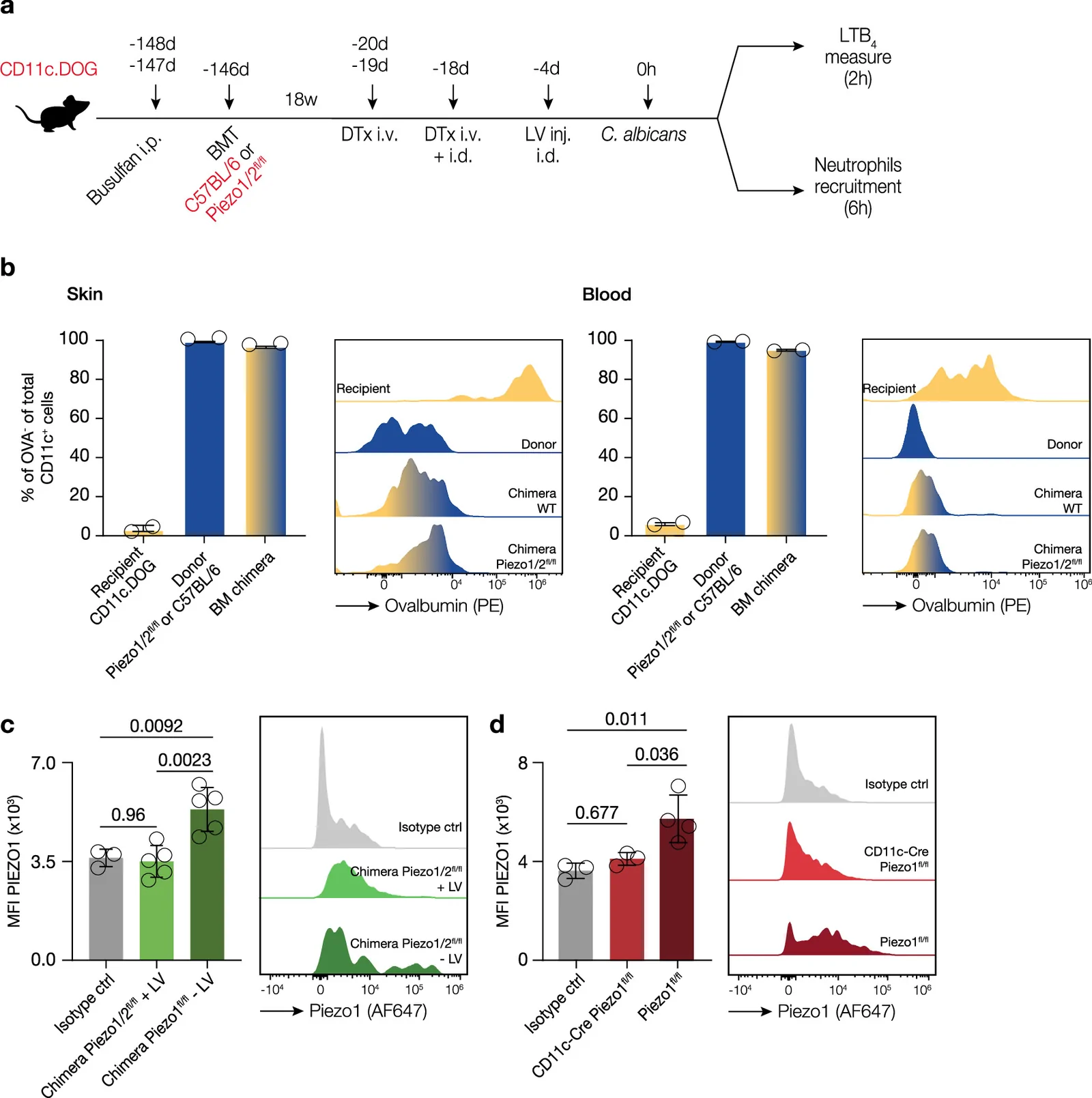
Generation and validation of CD11c.DOG bone marrow chimeras. **a**) Experimental timeline for the generation of CD11c.DOG chimeric mice. Recipient CD11c.DOG mice were treated with busulfan i.p. on days −148 and −147, followed by bone marrow transplantation on day −146 using C57BL/6 or *Piezo1/2*^*fl/fl*^ donor bone marrow. Eighteen weeks later, mice received DTx i.v. and/or intradermally to deplete the remaining host CD11c^+^ cells, followed by lentiviral vector injection and pathogen challenge. LTB_4_ levels were measured 2 h after infection, and neutrophil recruitment was assessed after 6 h. **b**) Assessment of donor chimerism in skin and blood CD11c^+^ cells. Bar graphs show the percentage of ovalbumin (OVA)-negative cells among total CD11c^+^ cells, and representative flow cytometry histograms are shown. The labels below the x-axis indicate the chimeric status of each group, distinguishing CD11c.DOG recipients and the corresponding donor bone marrow genotype (n = 2 for each group). Data are shown as mean ± SD. **c**) Representative histograms and quantifications showing *Piezo1* expression in CD11c^+^ cells in the indicated chimeric mice after treatment with lentiviral particles (LV) expressing CRE recombinase. Bar graphs represent median fluorescence intensity (MFI) shown as mean ± SD. Isotype control, n = 3 mice; +LV and −LV, n = 5 mice per group. **d**) Representative histograms and quantifications showing *Piezo1* expression in CD11c^+^ cells of CD11c-CRE x *Piezo1*^*fl/fl*^ mice (n = 3) and their control littermates (n = 4). Isotype is shown (n = 3). “n” indicates the number of biologically independent samples. Statistical significance was determined using one-way ANOVA followed by Tukey’s multiple-comparisons (**c,d**). *p* values are indicated. The “Rat” icon in panel a is by Uswa KDT, from Noun Project, CC BY 3.0; used without modification. Source data

To exclude the possible involvement of non-immune cells expressing Piezo1 in the recruitment process, we generated bone marrow chimeras in which recipient DOG mice were transplanted with bone marrow cells from *Piezo1*^fl/fl^*Piezo2*^fl/fl^ (*Piezo1/2*^fl/fl^) mice^38^. Eighteen weeks after transplantation, mice were treated with DTx, both intravenously (i.v.) and subcutaneously (s.c.), to eliminate any possible residual DOG DCs (Extended Data Fig. 9a,b). The mice were then treated s.c. with lentiviral particles expressing the CRE recombinase, and Piezo1 ablation was observed in CD11c^+^ cells (Extended Data Fig. 9c). This was sufficient to reduce LTB_4_ production and neutrophil recruitment (Fig. 5j). Finally, we backcrossed *Piezo1/2*^fl/fl^ mice with CD11c-CRE mice and selected *Piezo1*^fl/fl^ × CD11c-CRE F2 mice, which we then infected with *C. albicans*. In these settings as well, deletion of Piezo1 in CD11c^+^ cells significantly reduced neutrophil recruitment and LTB_4_ levels (Fig. 5k and Extended Data Fig. 9d).

These findings collectively indicate that Piezo1 activation, by inducing LTB_4_ synthesis, is crucial for early neutrophil migration to the infection site and that CD11c^+^ cells are the main initial source of this lipid mediator.

### Microbial adhesion mediates LTB_4_ production by CD11c^+^ cells

**Fig. 6:**
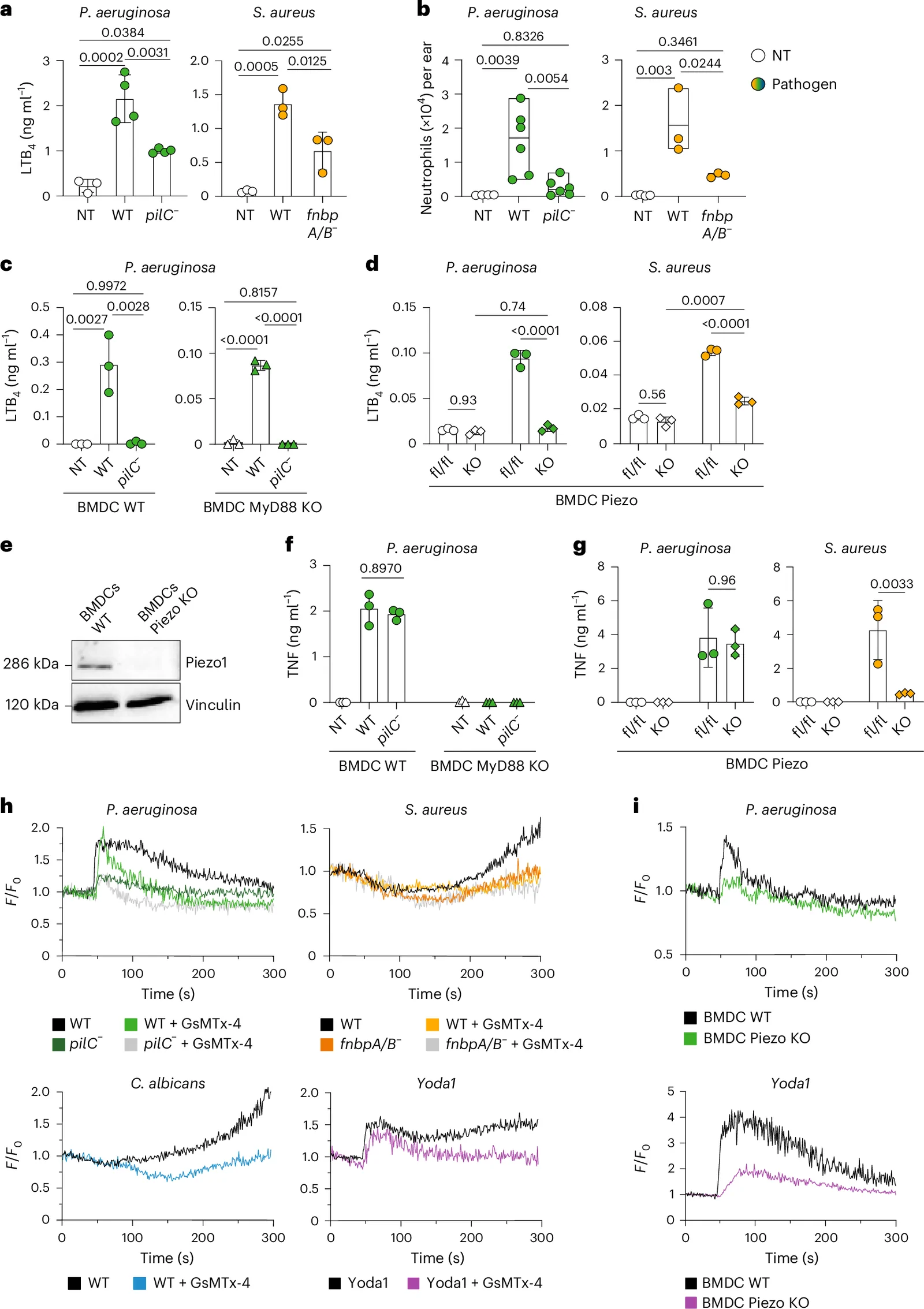
Pathogen adhesion triggers Piezo1-dependent LTB_4_ production. **a**, LTB_4_ production in mice 2 h after infection with WT or *pilC*^−^ *P. aeruginosa* and WT or *fnbA*^*−*^*fnbB*^−^ (*fnbA/B*^−^) *S. aureus*. For *P. aeruginosa*: NT, *n* = 3; WT, *n* = 4; and *pilC*^−^, *n* = 4. For *S. aureus*, *n* = 3 mice per group. NT, not treated. **b**, Neutrophil recruitment in mice 6 h after infection with WT or *pilC*^−^ *P. aeruginosa* and WT or *fnbA/B*^−^ *S. aureus*. For *P. aeruginosa*: NT, *n* = 4; WT and *pilC*^−^, *n* = 6. For *S. aureus*: NT, *n* = 4; WT and *fnbA/B*^−^, *n* = 3. **c**, LTB_4_ production by WT and *MyD88*^−/−^ BMDCs infected with WT or *pilC*^−^ *P. aeruginosa*. For each group *n* = 3. **d**, LTB_4_ production by *Piezo1/2*^fl/fl^ BMDCs that were or were not treated with the lentiviral vector carrying the CRE recombinase to induce excision of floxed exons. For each group, *n* = 3. **e**, Western blot showing Piezo1 depletion in *Piezo1/2*^fl/fl^ BMDCs treated with CRE, as specified in **d**. **f**, TNF production by WT and *MyD88*^*−/−*^ BMDCs treated with WT and *pilC*^−^ *P. aeruginosa*. For each group, *n* = 3. **g**, TNF production by WT and Piezo1-deficient BMDCs treated with WT *P. aeruginosa* or *S. aureus*. For each group, *n* = 3. **h**, Representative Ca^2+^ mobilization kinetics in D1 cells infected with the indicated WT and mutant pathogens, or Yoda1 with or without the Piezo1 inhibitor GsMTx-4. **i**, Representative Ca^2+^ mobilization kinetics in *Piezo1/2*^fl/fl^ BMDCs that were or were not treated with the lentiviral vector carrying the CRE recombinase upon exposure to *P. aeruginosa* or Yoda1 as a positive control. Fluorescence intensity is expressed as *F*/*F*_0_, where *F*_0_ is the mean baseline fluorescence before stimulation. Each condition (**h–i**) was analyzed in a separate tube containing 5 × 10^5^ cells and acquired in real time through flow cytometry. Traces are representative of at least three independent experiments. In **b**, data are shown as mean and range (minimum to maximum). In a, **c**, **d**, **f** and **g**, data are shown as mean ± s.d. *n* indicates the number of biologically independent samples; each dot represents a mouse. Statistical significance was determined using one-way ANOVA followed by Tukey’s multiple comparisons (**a–c**) or two-way ANOVA followed by Tukey’s (**d**,**f**,**g**) test. *P* values are indicated. Source data

Adhesion to host cells and tissues is an early event during infections that requires specific molecular machinery such as type IV pili^39,40^ in *P. aeruginosa*^41^, fibronectin-binding proteins in *S. aureus*^42^ and several adhesins in *C. albicans*. Upon adhesion, these microbes stretch the host cell membrane, exerting nanonewton mechanical forces of the same magnitude as those that activate Piezo channels^43^. We hypothesized that adhesion to the plasma membrane could lead to LTB_4_ production. Infection with *P. aeruginosa* lacking pili (*pilC*^*−*^) or *S. aureus* lacking fibronectin-binding proteins (*fnbA*^*−*^*fnbB*^*−*^)^44^ resulted in strongly reduced release of LTB_4_ and neutrophil recruitment compared with that in the WT strains (Fig. 6a,b).

**Extended Data Fig. 10:**
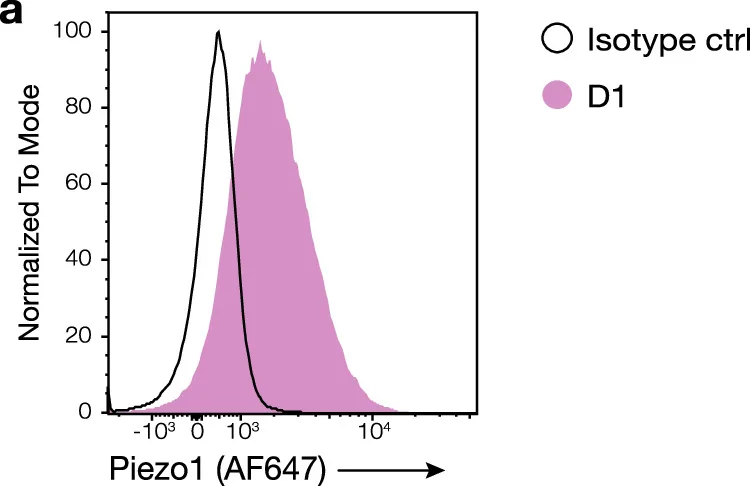
Characterization of Piezo1 expression in D1 cells. a) Representative histogram of Piezo1 expression levels in D1 cells. Isotype control is also shown.

In agreement with these observations, *Piezo1*-sufficient bone-marrow-derived DCs (BMDCs) challenged with WT or mutant pathogens produced LTB_4_ in an adhesion-dependent, MyD88-independent manner (Fig. 6c), whereas *Piezo1*-deficient BMDCs failed to mount an analogous response (Fig. 6d,e). Both WT and mutant *P. aeruginosa* stimulated tumor necrosis factor (TNF) secretion in Piezo1-deficient but not in MyD88-deficient BMDCs, whereas *S. aureus* required both MyD88 and Piezo1 to induce the production of TNF (Fig. 6f,g). We finally used Ca^2+^ mobilization as a more direct readout of Piezo1 activation. We performed this experiment in D1 cells, which express Piezo1 homogeneously (Extended Data Fig. 10) and display a sufficiently synchronous response upon microbial stimulation to allow measurement of Ca^2+^ fluxes through fluorescence-activated cell sorting (FACS) analysis. As shown in Figure 6h, Ca^2+^ mobilization was significantly reduced in the presence of mutant compared with WT bacteria. Similarly, the Piezo inhibitor GsMTx-4 reduced Ca^2+^ mobilization in cells exposed to bacteria or *C. albicans*, mainly affecting the sustained phase of the response. This pattern was consistent with that observed upon Yoda1 stimulation, where Piezo inhibition selectively impaired the sustained phase of Ca^2+^ mobilization (Fig. 6h). Given the robust response induced by *P. aeruginosa*, we could measure Ca^2+^ mobilization in BMDCs, which are more asynchronous and heterogeneous than D1 cells. In this case, we found an almost complete inhibition in *Piezo1*-deficient cells (Fig. 6i).

Together, these data show that LTB_4_ can be produced in a Piezo1-dependent but TLR-independent manner, induced by pathogen adhesion to myeloid cells.

### IL-1α inhibits TLR signaling

**Fig. 7:**
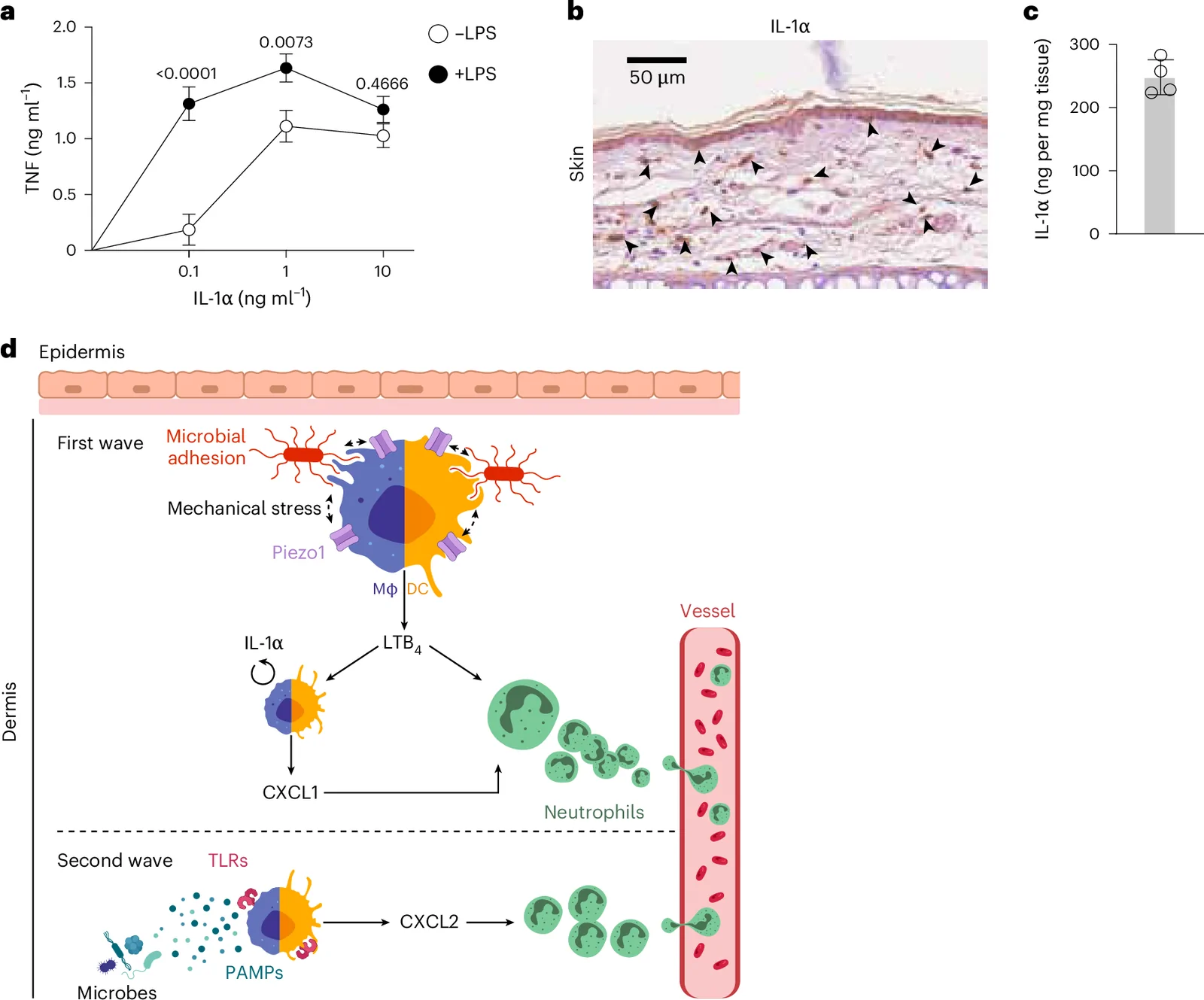
IL-1α inhibits LPS signaling in DCs. **a**, TNF released by DCs prestimulated with increasing doses of IL-1α (0.1, 1 and 10 ng ml^−1^) or that were not treated, followed by the addition of a saturating dose of LPS (5 μg ml^−1^). Levels of released TNF were measured 3 h after LPS administration. Data are shown as mean ± s.e.m., and the data are representative of three independent experiments, each performed in technical triplicates. *P* values are indicated. **b**, Representative immunohistochemistry image of an ear skin section showing IL-1α-positive cells (indicated by arrows). **c**, IL-1α quantification in mouse skin ears. IL-1α was quantified by ELISA in tissue homogenates from ears of naive mice. Data are presented as the mean of total IL-1α (normalized to the weight of the ear) ± s.d. (*n* = 4). **d**, Schematic of the two-step mechanism driving neutrophil recruitment during skin infection. Microbial adhesion to myeloid cells applies membrane tension that promotes the activation of the mechanosensitive channel Piezo1, leading to LTB_4_ production and IL-1α release. IL-1α triggers CXCL1 expression and an early wave of neutrophil infiltration. In a second phase, pathogen-derived PAMPs activate TLRs, inducing CXCL2 release and sustaining neutrophil recruitment. *n* indicates the number of biologically independent samples. Statistical significance in **a** was determined using two-way ANOVA followed by Šidák’s multiple comparisons test. Schematic in **d** created in BioRender and subsequently edited in Adobe Illustrator; Cozzi, S. https://biorender.com/k3n53j6 (2026). Source data

The paramount role of DAMPs as triggers of inflammation in response to microbes capable of activating TLRs raises the question as to why these receptors play a role only late after infection. Although a possible explanation could be that only modified PAMPs are recognized^5^, the observations that the first responders to infections are CD11c^+^ cells (Fig. 5f) and that both early (IL-1α-dependent) and late (IL-1α-independent) neutrophil recruitment require MyD88 (Fig. 2) suggested that IL-1α and TLR signaling could be competitive. To test this alternative hypothesis, we first determined the saturating dose of IL-1α in the D1 DC line: dose–response experiments revealed that the secretion of TNF, a bona fide readout of MyD88-dependent signaling^45^, plateaued at a concentration between 1 and 10 ng ml^−1^ (Fig. 7a). We then tested whether a pre-exposure of DCs to IL-1α could inhibit a subsequent response to PAMPs and found that a saturating IL-1α concentration rendered the D1 cells insensitive to lipopolysaccharide (LPS) (Fig. 7a). Hence, IL-1α can effectively outcompete LPS in DCs. Strikingly, mouse ears contained approximately 250 ng mg^−1^ of IL-1α (Fig. 7b,c). As such, IL-1α may desensitize TLRs to their agonists early after infection, even if it is released in very low quantities in the dermis.

## Discussion

Here, we investigated how neutrophils are recruited at microbial infection sites. We used a skin infection model to study the response to different pathogens, including fungi, Gram-positive bacteria and Gram-negative bacteria. It is widely accepted that immune responses are primarily initiated by the biochemical sensing of PAMPs. However, our findings show that mechanosensing of microbial adhesion plays a pivotal role. This promotes the activation of Piezo1 and the ensuing production of LTB_4_, which, together with IL-1α, induces the release of CXCL1, initiating neutrophil migration towards the site of infection. In line with this, our in vitro experiments showed that LTB_4_ can be produced in an adhesion-dependent, Piezo1-dependent manner, independently of MyD88 even in the presence of PAMPs. This result highlights that microbial adhesion provides a mechanosensory input sufficient for the production of key molecules necessary to activate innate immunity, uncovering an early PRR-independent pathway that in vivo is sufficient to induce neutrophil recruitment. We identified CD11c^+^ myeloid cells as the key mechanoresponsive, LTB_4_ and CXCL1-producing cells in vivo (Fig. 7d). This mechanosensitive pathway highlights a conserved mechanism whereby the immune system rapidly responds to infections, even before the identification of the type of insult. This would enable the innate immune response to occur promptly, particularly if PRRs do not recognize infectious microorganisms per se, but rather altered PAMPs^5^, which need time to be generated and to accumulate at the infection site. Effective coordination of this two-tier response is achievable because IL-1α and TLRs share some adapter molecules for signaling. Activation of tissue-resident myeloid cells with IL-1α makes them unable to respond to PAMPs, likely because robust IL-1α stimulation promotes the formation of long-lived multi-Myddosome clusters^46^, thereby sequestering MyD88 (ref. ^47^). This would favor a rapid, non-specific response aimed at confining the pathogen and limiting its spread before engaging more specific PRR-mediated mechanisms of pathogen clearance. It is intriguing that LTB_4_ bridges the mechano- and chemo-responsive signaling cascades regulating inflammation, because it also functions as a potent chemoattractant for, and as an activator of, neutrophils^16,48^. The dependence of LTB_4_ production on Piezo1 activation reveals that the detection of mechanical changes on the surface of myeloid cells is pivotal to achieve immune readiness. Such a mechanism ensures that the body can swiftly react to potentially harmful challenges by deploying neutrophils that, in cases of infection, can contain the threat to minimize tissue damage^49^. Moreover, the transition from a generalized mechanosensor-driven mechanism to a more targeted PRR-dependent mechanism of neutrophil recruitment and activation allows the host to mount an immune response finely tuned to counter the pathogen and eliminate the infection. As such, this two-tier response is well suited to achieve an initial rapid containment of possible microbial threats that enables a subsequent local attack against the specific invading pathogen. The distinct roles and temporal regulation of (IL-1α-dependent) CXCL1 and (PRR-dependent) CXCL2 production in the early and late phases of the innate immune response coordinate this process.

The pivotal role of IL-1α in the recruitment of neutrophils is well documented across various types of infections, encompassing both bacterial and fungal pathogens. In the context of viral infections, the role of IL-1α has been examined in respiratory viral infections, during which it contributes to the recruitment of neutrophils to the lungs^50^. This is particularly important for controlling viral replication and initiating the adaptive immune response, although it must be carefully tweaked to prevent excessive tissue damage. The activity of IL-1α against a very broad spectrum of pathogens defines it as a cornerstone of the innate immune response.

Nevertheless, the mechanisms underpinning the release of IL-1α at the infection sites, and how this induces the recruitment of neutrophils, had not yet been clarified. In this regard, we show that IL-1α is induced in a LTB_4_-dependent manner upon activation of Piezo1, highlighting that mechanical signals and mechanoreceptors are fundamental to initiating the immune response by activating the biochemical pathways that promote neutrophil recruitment. The observation that only the concomitant blockade of IL-1 signaling and LTB_4_ synthesis can fully prevent the recruitment of neutrophils during infections suggests that mechanosensing and passive IL-1α release following cell death represent the two major danger signals.

In summary, the findings presented herein enrich our understanding of innate immunity by showing that danger signals are the trigger for innate immune responses, even in the presence of infections. In addition, they pave the way for therapeutic approaches to modulate the immune response. Targeting these pathways could lead to treatments for inflammatory diseases, during which excessive or misdirected neutrophil recruitment contributes to pathology. Similarly, enhancing the body’s mechanosensitive response to infection could offer alternative strategies for boosting the immune defense against pathogens.

## Methods

### Mice

Mice were maintained and bred in a pathogen-free environment at the animal facilities of the University of Milano Bicocca. Mice were fed a normal chow diet and housed at 20 ± 2 °C, with 55 ± 10% relative humidity under a 12 h light/dark cycle, with lights on at 7:00 and off at 19:00. Males and females aged 6–9 weeks and matched for age and sex were used during the procedures. Littermates of the same genotype and age were randomly assigned to experimental groups. The animal studies were conducted after approval by the Italian Ministry of Health (approval no. 592/2020-PR) and in accordance with institutional guidelines. WT C57BL/6 mice were supplied by Envigo*. Card9*^−/−^,*Tlr2*^−/−^ and CD11c-CRE mice were purchased from the Jackson Laboratory. *Il1r1*^−/−^ mice^51^ were provided by C. Garlanda (Humanitas Research Institute), *Myd88*^−/−^ and *Tlr4*^−/−^ mice by S. Akira (IFReC), CD11c.DOG mice^37^ (in which a specific CD11c^+^ cell ablation can be induced by DT injection) by N. Garbi (Institute of Molecular Medicine and Experimental Immunology) and *Piezo1*^fl/fl^*Piezo2*^*f*l/fl^ mice were provided by L. Catherine (INSERM UMR_S1255). All mutant mouse lines were maintained on the C57BL/6 background.

### Bacterial/fungal culture and mouse infections

The *C. albicans* strain CAF3-1 (ura3Δ::imm434/ura3 Δ::imm434), provided by W. A. Fonzi (Georgetown University), was grown at 25°C in rich medium (YEPD (yeast extract, peptone, dextrose), 1% (wt/vol) yeast extract, 2% (wt/vol) Bacto Peptone and 2% (wt/vol) glucose supplemented with uridine (50 mg l^−1^), as described^52^. In this medium, cells showed a typical yeast morphology, and growth was monitored by counting the cell number using a Coulter Counter Particle Count and Size Analyzer. Once cells reached a concentration of about 8 × 10^6^ cells ml^−1^, the total culture was collected by centrifugation and resuspended in an equivalent volume of YEPD uridine medium buffered with Hepes (50 mM, pH 7.5). Cells were incubated at 37 °C for hyphal induction. Formation of hyphae was evaluated under a microscope at different time points following induction, until 95% of cells had formed hyphae. The *P. aeruginosa* strain PAO1, the isogenic *pilC* mutant, the *S. aureus* strain ATCC6538P and the isogenic *fnbAB*^−/^^*−*^ mutant were cultured in LB medium (Difco) at 37 °C. For s.c. infections, stationary phase cultures were diluted to an optical density at 600 nm (OD_600_) of 0.05 and then grown until they reached an OD_600_ of 0.25, corresponding approximately to 1 × 10^6^ colony-forming units (CFU) ml^−1^. Cells were washed in PBS and appropriately diluted before injection.

Pathogens were intradermally injected into the left or the right mouse ear or footpad in 30 μl of PBS with a 30-G syringe. *C. albicans* was injected at 1 × 10^6^ hyphae per site*, P. aeruginosa* at 5 × 10^7^ CFU per site and *S. aureus* at 1 × 10^8^ CFU per site.

For the depletion of CD11c^+^ cells, CD11c.DOG mice were injected at day –3, –2 and –1 with DTx, i.v. (16 ng g^−1^ body weight) and at day –1, also in the footpad (16 ng per footpad) to achieve a complete depletion. The efficacy of the treatment was verified through flow cytometry by staining DCs and macrophages taken from the ears and from the spleen at 6 and 24 h postinfection (that is, 24 and 48 h after the last treatment).

For in vivo neutralization of IL-1α and IL-1β, IL-1α- and IL-1β-specific neutralizing antibodies or control isotypes (10 μg ml^−1^) were co-injected along with the pathogen in the ears.

For in vivo neutralization of CXCL1 and CXCL2, CXCL1- and CXCL2-specific neutralizing antibodies or control isotypes were injected i.v. (30 μg per mouse) 30 min before the co-injection of the antibodies (3 μg per ear) and the pathogens.

To inhibit LTB_4_ synthesis, mice were injected with bestatin (LTA_4_ hydrolase inhibitor) i.p. (15 mg kg^−1^ body weight) at day –1, day 0 and 10 h postinfection. These animals were euthanized at 24 h of infection. For CXCR2 inhibition, mice were treated with the specific inhibitor SB225002 i.p. (15 mg kg^−1^ body weight) 30 min before pathogens were injected. To ensure CXCR2 blockade, SB225002 (15 mg kg^−1^ body weight) was also injected i.p. 10 h after infection. For Piezo1 inhibition, mice were treated i.p. (1 mg kg^−1^ body weight) and locally in the footpad with GsMTx-4 (6 μM) 30 min before injecting the pathogens. To ensure Piezo1 blockade, GsMTx-4 was also co-injected with the pathogen in the footpad (6 μM). All the inhibitors were obtained from Selleck Chemicals.

### Production of lentiviral particles and infections

Lentiviral vectors (LVs) were obtained by transfecting 293T cells with a solution containing a mix of the selected LV genome transfer plasmid (pCCLsin.PPT.hPGK.NLS-Cre(L46Q).Wpre), the packaging plasmids pMDLg/pRRE and pCMV.REV, pMD2.G and pAdvantage, as previously described^53^. Medium was changed 14 to 16 h after transfection, and supernatant was collected 30 h after the medium was changed. Vector-containing supernatants were passed through a 0.22-μm filter, transferred into sterile polyallomer tubes and centrifuged at 20,000*g* for 120 min at 20 °C (Beckman Optima XL-100 K Ultracentrifuge). The LV pellet was resuspended in PBS to obtain a 500× or 1,000× concentration.

For the LV titer, 100,000 HEK293T cells were transduced with serial dilutions in the presence of polybrene (8 μg ml^−1^). Genomic DNA was extracted 10–14 days after transduction, using the Maxwell 16 Cell DNA Purification Kit (Promega), following the manufacturer’s instructions. Viral copy number (VCN) was determined by droplet digital PCR, starting from 15 ng of template DNA using primers (HIV forward (Fw): 5′-TACTGACGCTCTCGCACC-3′; HIV reverse (Rv): 5′-TCTCGACGCAGGACTCG-3′) and a probe (FAM 5′-ATCTCTCTCCTTCTAGCCTC-3′) against the primer binding-site region of LV. The amount of endogenous human DNA was quantified by a primer–probe set against the telomerase gene *(TELO* Fw: 5′-GGCACACGTGGCTTTTCG-3′; *TELO* Rv: 5′-GGTGAACCTCGTAAGTTTATGCAA-3′) and a probe *(VIC* 5′-TCAGGACGTCGAGTGGACACGGTG-3′ TAMRA). The PCR was performed with each primer (900 nM) and the probe (250 nM), following the manufacturer’s instructions (Bio-Rad), read with a QX200 reader and analyzed with QuantaSoft software (Bio-Rad). The infectious titer was expressed as transducing units (TU) ml^−1^ and calculated as VCN × number of cells (100,000) × (1 / dilution factor). Specifically, VCN = (copies of genome-integrated LV / copies of genomic DNA, *TELO*) × 2 (HEK293T considered as diploid cells). As a positive control, a CEM cell line stably carrying four vector integrants (VCN = 4) was used.

Mice were infected by injecting 5 ×10^7^ particles in 20 µl. BMDCs were transduced at MOI 5, and the medium was replaced 18 h postinfection.

### In vitro DC stimulation

D1 cells were cultured as previously described^36^. To evaluate LTB_4_ production following the exposure to the Piezo agonist, 200,000 D1 cells were plated in a 96-well plate 2 h before stimulation with Yoda1 (40 µM; Merck Life Science).

To determine whether IL-1α could outcompete LPS signaling, D1 cells were treated with increasing doses of recombinant mouse IL-1α (Biolegend) for 10 min before the addition of a high dose of LPS (5 μg ml^−1^).

BMDCs were generated as follows: bone marrow was flushed from the femurs and DCs were differentiated in complete IMDM supplemented with 10% supernatant from B16 tumor cells expressing GM-CSF. After 8 days, DC differentiation was evaluated on the basis of the expression of CD11c, I-Ab, CD86 and CD80. On day 9, 200,000 BMDCs were plated in a 96-well plate 2 h before stimulation. For the generation of Piezo1/2-deficient BMDCs, 5 days after plating bone marrow cells from *Piezo1/2*^fl/fl^ mice, cultures were transduced overnight with lentiviral particles encoding Cre recombinase at an MOI of 5. The following day, the medium was replaced with fresh medium, and cells were allowed to differentiate until day 8.

When indicated, BMDCs were pretreated with GsMTx-4 (6 μM; Selleckchem) or vehicle (PBS) as a control, 30 min before microbial exposure.

In all experiments, we collected the supernatant 3 h after stimulation to measure LTB_4_ (Leukotriene B4 Express ELISA Kit 500003, Cayman Chemical) or TNF release (88-7324-88, Thermo Scientific) by ELISA, according to the manufacturer’s instructions.

### Isolation of skin cells

Mouse skin cells were isolated as previously described^39^. In brief, mouse ears were amputated, split into dorsal and ventral halves and cut into small pieces in digestive medium (RPMI 1640, 5% FBS, L-glutamine, penicillin and streptomycin, liberase 300 μg ml^−1^, DNase I 50 U ml^−1^). After 90 min at 37 °C, digestion was stopped with prechilled complete RPMI medium (RPMI 1640, 10% FBS, L-glutamine, penicillin and streptomycin). The cells were then passed through a 5-ml syringe and filtered through a 70-µm cell strainer (Greiner) to obtain a homogeneous cell suspension. The skin of the footpad was digested with the same protocol, but it was detached from the amputated footpad before digestion.

### Flow cytometry

Multi-parametric flow cytometry analyses were performed on a Cytek Aurora (Cytek), and data were analyzed using FlowJo (BD Biosciences). Fluorochrome-conjugated antibodies (BioLegend, BD Biosciences or Thermo Fisher Scientific) are listed in Supplementary Table 1.

Single-cell skin suspensions were first incubated with LIVE/DEAD Fixable Scarlet 723 viability dye (Thermo Fisher Scientific) for 15 min at room temperature to exclude dead cells. After this, the cells were washed with FACS buffer (PBS, 0.5% BSA and 2 mM EDTA), blocked for 20 min at 4 °C with Fc receptor blocking solution containing anti-CD16/32 and stained for 20 min at 4 °C with fluorochrome-conjugated antibodies directly added to the blocking solution. The cells were subsequently fixed with BD Cytofix (BD Biosciences) for 15 min at 4 °C, washed three times with PBS and transferred into flow cytometry tubes for acquisition.

For intracellular CXCL1 staining, skin-tissue processing and all subsequent staining procedures were performed in the presence of Brefeldin A (5 μg ml^−1^) to inhibit cytokine secretion. Following surface staining as described above, the cells were fixed and permeabilized overnight at 4 °C using the Foxp3/Transcription Factor Fixation/Permeabilization kit (Thermo Fisher Scientific), according to the manufacturer’s instructions. The following day, the cells were stained with anti-CXCL1 AF647 or the corresponding isotype control, washed with FACS buffer and acquired on the same day.

For Piezo1 and Piezo2 detection, single-cell skin suspensions were first incubated with LIVE/DEAD Fixable Near-IR 780 viability dye (Thermo Fisher Scientific) for 15 min at room temperature. After being washed with FACS buffer, the cells were blocked for 20 min at 4 °C with Fc receptor blocking solution containing anti-CD16/32. Unconjugated rabbit anti-mouse primary antibodies to Piezo1 or Piezo2 were directly added to the blocking solution, and the cells were incubated for 30 min at 37 °C. The cells were subsequently washed with FACS buffer, centrifuged (500*g*, 5 min, 4 °C) and stained with Alexa Fluor 647-conjugated goat anti-rabbit secondary antibody for 20 min at 4 °C. After washing with FACS buffer, the cells were centrifuged again (500*g*, 5 min, 4 °C) and stained with the following fluorochrome-conjugated surface antibodies: Ly6G, CD45, F4/80, PDGFRα, CD31, CD11c and CD3. Following surface staining, the cells were fixed and permeabilized using the Foxp3/Transcription Factor Fixation/Permeabilization kit (Thermo Fisher Scientific), according to the manufacturer’s instructions, and subsequently stained with anti-pan-cytokeratin antibody to identify keratinocytes.

### RNA extraction and RT–qPCR

Ears and footpads were put in TriReagent (Sigma-Aldrich) immediately after explant and stored at –80 °C. Before RNA extraction with silica columns, tissue was mechanically digested for 20 min at 4 °C, using a TissueLyser (Qiagen) at 20 Hz. Next, nucleic acids were isolated through the phenol-chloroform extraction method.

Afterwards, total RNA was extracted from the nucleic-acid solution using the Zymo Miniprep kit, per the manufacturer’s instructions. Complementary DNA (cDNA) was generated with LunaScript RT SuperMix Kit (New England Biolabs). Quantitative real-time PCR was done using the TaqMan Gene Expression Assay, a universal PCR master mix, and the Applied Biosystems 7500 Real Time PCR system with commercial primers and probes from Thermo Fisher Scientific (Supplementary Table 1). Gene expression was normalized with respect to either *Gapdh* or *Rn18s*, depending on the experiment.

### Measurement of LTB_4_ concentration and IL-1α in the skin

LTB_4_ concentration in mouse skin was determined using an enzyme immunoassay kit (Leukotriene B4 Express ELISA Kit 500003, Cayman Chemical), according to the manufacturer’s instructions. ELISA was performed on lipid-enriched samples. In brief, tissues were homogenized in PBS and 2 mM EDTA for 8 min at 4 °C, using a TissueLyser (Qiagen) set at 30 Hz. Following this first step, proteins were precipitated by adding 4 volumes of ice-cold absolute ethanol to the supernatant. The enriched lipid fraction was then dried using a rotary evaporator, and lipids were resuspended in the ELISA buffer included in the kit.

To quantify the amount of IL-1α in naive skin tissue, ear samples were minced in RIPA 1× Buffer (cat. no. 9806, Cell Signaling Technology), supplemented with protease and phosphatase inhibitors (cat. nos. 5871 and 5870, Cell Signaling Technology), and then homogenized using a TissueLyser (Qiagen) set to 30 Hz for 8 min at 4 °C. The samples were centrifuged at 1,000*g* for 5 min at 4 °C to remove debris. The supernatant was passed through a 30-G syringe and centrifuged for 10 min at 16,000*g* at 4 °C, followed by another centrifugation for 10 min at 20,000*g* at 4 °C to eliminate lipids and nucleic acids. The protein supernatants were then used to quantify IL-1α, following the manufacturer’s instructions (DY400, R&D Systems).

### Generation of CD11c.DOG bone-marrow chimeras

Six-week-old male and female CD11c.DOG mice were used as recipients for the generation of bone-marrow chimeras. Recipient mice were treated with busulfan (20 mg kg^−1^ body weight) by intraperitoneal injection on days −148 and −147 before infection, to allow efficient bone-marrow engraftment. On day −146, mice received bone-marrow transplantation using donor bone-marrow cells isolated from either C57BL/6 or *Piezo1/2*^fl/fl^ mice. Reconstituted mice were maintained for 18 weeks to allow hematopoietic chimerism to develop.

Eighteen weeks after bone-marrow transplantation, CD11c^+^ cells of recipient origin were depleted by administration of DTx. DTx (16 ng g^−1^ body weight) was injected i.v. on days −20 and −19, followed by combined intravenous and intradermal administration on day −18. This treatment was used to eliminate residual CD11c.DOG recipient-derived CD11c^+^ cells before subsequent experimental procedures. To validate bone marrow reconstitution, donor chimerism was assessed in both skin and blood CD11c^+^ cells. Cells were analyzed by flow cytometry for ovalbumin expression, which was used to distinguish donor-derived cells (ovalbumin negative) from recipient-derived cells (ovalbumin positive).

Four days before the pathogen challenge, mice received an intradermal injection of a lentiviral vector (5 × 10^7^ particles). On day 0, mice were challenged with the indicated pathogen. LTB_4_ production was measured 2 h after the pathogen challenge, whereas neutrophil recruitment was assessed 6 h after the challenge.

### In vivo knockdown of Piezo1

To silence Piezo1 in vivo, 5 μg of *Piezo1*-targeted siRNA (Ambion In Vivo Pre-Designed siRNA Invitrogen, Thermo Fisher Scientific) or scrambled siRNA (Ambion In Vivo Negative Control #1 siRNA Invitrogen, Thermo Fisher Scientific) was mixed with 0.6 μl in vivo-jetPEI transfection reagent (in vivo-jetPEI, Polyplus) and injected s.c. in the footpads of C57BL/6J mice in a final volume of 30 μl. After 24 h, these mice were injected with the pathogen and further studied. The knockdown efficiency was evaluated by quantitative PCR, assessing the expression of *Piezo1* and the reference gene *Gapdh* in scramble siRNA- or si*Piezo1*-treated tissues.

### Immunohistochemistry

Explanted skin samples were fixed in 4% paraformaldehyde (PFA) and embedded in paraffin. Sections (7 μm thick) were cut using a microtome and mounted on Superfrost Plus slides (Thermo Scientific). After deparaffinization, the sections were blocked and permeabilized with 0.2% BSA (bovine serum albumin) and 0.1% Triton X-100 for 10 min at room temperature. They were then incubated overnight at 4 °C with a primary antibody to IL-1α (15 μg ml^−1^ in PBS with 2% BSA, AF-400 NA R&D Systems). After washing with Tris-buffered saline, the sections were labeled for 30 min at room temperature using the Biocare Medical Goat-on-Rodent HRP-Polymer (GHP516, Biocare Medical), according to the manufacturer’s instructions, and counterstained with Meyer’s hematoxylin solution (Bio-Optica). After dehydration, the stained slides were mounted using Eukitt, and images were acquired using the NanoZoomer (Hamamatsu).

### Tissue preparation and multiplex immunofluorescence imaging

#### Mouse treatment and tissue collection

CD11c.DOG and C57BL/6J mice received three systemic injections of diphtheria toxin (DTx; 16 ng/g body weight, intravenous) on days −3, −2 and −1, or PBS as a control. A local injection of DTx (16 ng) was administered subcutaneously on day −1. Mice were euthanized on day 0 in accordance with national and international animal care regulations. A square section of flank skin was excised from each mouse at the site of the last intradermal injection, or from the anatomically corresponding region in C57BL/6J control mice. Subcutaneous fat was removed mechanically using forceps. Skin samples were processed according to the 2D Manual IBEX protocol, as described previously^54^.

#### Fixation, dehydration, and embedding

Samples were fixed overnight (~16 h) in 2 ml of a solution containing 500 µl BD Cytofix/Cytoperm buffer (BD Biosciences) and 1500 µl PBS (pH 7.4). Tissues were dehydrated for 24 h in 15 ml of PBS containing 30% sucrose (wt/vol), embedded in Optimal Cutting Temperature (OCT) compound (Histo-Line Laboratories), and stored at −80 °C for 24 h.

#### Cryosectioning and blocking

Tissues were cryosectioned at 8 µm thickness using a Leica cryostat (Leica Biosystems) and mounted onto gelatin-coated Superfrost Plus slides (Epredia). Non-specific binding was blocked and nuclei counterstained by incubating each section for 1 h at 37 °C in 100 µl of blocking buffer containing 1% normal goat serum (Vector Laboratories), 20 µg ml^−1^ anti-CD16/32 (clone 93, BioLegend, cat. no. 101302), and 0.1 µg Hoechst 3342 (Thermo Fisher Scientific). Slides were washed three times for 5 min in 35 ml PBS (pH 7.4).

#### Primary and secondary staining

Tissue sections were incubated with 100 µl of staining solution containing 1.8 µg of unconjugated rabbit anti-Piezo1 antibody (Proteintech, cat. no. 15393-1-AP, lot. no. 00152477) for 1 h at 37 °C. A single C57BL/6J skin section served as an isotype control and was incubated with 1.8 µg of rabbit polyclonal isotype control antibody (R&D Systems, cat. no. AB-105-C). After washing, sections were incubated with 100 µl of Alexa Fluor 647-conjugated goat anti-rabbit secondary antibody (0.5 µg; Invitrogen, cat. no. A-21245) for 30 min at 37 °C.

#### IBEX iterative staining and bleaching

Following secondary staining, all sections were incubated with 100 µl of cycle 1 antibody mix for 1 h at 37 °C (see Supplementary Table 1 for antibody panel details). After washing, samples were mounted with Fluoromount G (Thermo Fisher Scientific, cat. no. 00-4958-02) and imaged on a Leica Thunder DM6B microscope equipped with a LED3 white light source, six excitation (390 ± 20 nm, 435 ± 10 nm, 480 ± 20 nm, 555 ± 10 nm, 580 ± 10 nm and 640 ± 15 nm) and six emission (435 ± 15 nm, 480 ± 20 nm, 515 ± 15 nm, 595 ± 18 nm, 630 ± 30 nm and 690 ± 30 nm) bandpass filters, and a ×40 objective (NA 0.8). Real-time denoising was performed using the integrated Thunder algorithm (Leica LasX software, mounting media refractive index set to 1.4000).

After cycle 1 imaging, samples were chemically bleached by incubation in 1 ml of 1 mg ml^−1^ LiBH_4_ in ddH_2_O for 15 min at room temperature, followed by washing. Cycle 2 antibody mix was applied as described above, and slides were imaged again. After cycle 2, bleaching duration was extended to 30 min due to the presence of FITC-conjugated antibodies. The process was repeated for cycle 3.

#### Image alignment and analysis

Raw multichannel image stacks (.ome.tiff) from all cycles were imported into HALO (Indica Labs). Images were aligned using Hoechst 3342 nuclear staining as a fiducial marker and the built-in HALO alignment algorithm. Nuclear and cytoplasmic segmentation was performed using the HALO Highplex FL module. The resulting segmented data were exported as .fcs files for further analysis in FlowJo (BD Biosciences).

#### Cell population identification

Spectral spillover between channels was manually compensated in FlowJo. Dermal cells were defined as pan-cytokeratin (PanCK)-negative cells within the tissue. CD11c^+^ cells were defined as CD11c^+^PDGFRα^−^CD31^−^PanCK^−^CD3^−^B220^−^. Dermal fibroblasts were identified as PDGFRα^+^CD11c^−^CD31^−^PanCK^−^CD3^−^B220^−^. Vascular structures were defined as CD31^+^CD11c^−^PDGFRα^−^PanCK^−^CD3^−^B220^−^.

#### Double-positive cell analysis

Piezo1/ALOX5 double-positive cells were quantified among dermal cells after exclusion of epidermis and PanCK^+^ keratinocytes (CD11c^−^PDGFRα^−^CD31^−^CD3^−^B220^−^). The Piezo1 positivity threshold was determined using the isotype control sample. CD11c^+^ cells, fibroblasts and vessels were further identified in the double-positive population on the basis of the marker criteria described above.

Each double-positive cell was visually validated using HALO to eliminate false positives. Contour plots of Piezo1 and ALOX5 expression were generated by comparing equal numbers of cells from DTx-depleted CD11c.DOG mice and PBS-injected CD11c.DOG control.

### Ca^2+^ measurements

A total of 5 × 10^5^ cells (D1 cells or BMDCs) were loaded with 3 μM Fluo-4 acetoxymethyl ester (Fluo-4 AM; Invitrogen) and incubated for 30 min at 37 °C in complete medium supplemented with probenecid (Invitrogen). Cells were then washed, centrifuged and resuspended in complete medium containing probenecid and equilibrated at 37 °C before acquisition. Ca^2+^ fluxes were subsequently analyzed by flow cytometry. Cells were stimulated with either *P. aeruginosa* or *S. aureus* (1.7 × 10^8^ CFU), *C. albicans* (5 × 10^4^ cells) or Yoda1 (40 μM; Merck Life Science). Where indicated, cells were pretreated with GsMTx-4 (5 μM; Selleckchem) during the loading with Fluo-4 AM, which was also added in the resuspension medium.

### Statistical analysis

Means were compared by either unpaired parametric *t*-tests or two-way ANOVA. Data are expressed and plotted as means ± standard deviations from the mean or means ± s.e.m. The sample size for each experimental condition is provided in the figure legends. All *P* values were calculated using Prism (GraphPad). Differences were considered significant if *P* ≤ 0.05.

### Reporting summary

Further information on research design is available in the Nature Portfolio Reporting Summary linked to this article.

## Online content

Any methods, additional references, Nature Portfolio reporting summaries, source data, extended data, supplementary information, acknowledgements, peer review information; details of author contributions and competing interests; and statements of data and code availability are available at https://doi.org/10.1038/s41590-026-02643-y.

## Supplementary information

Supplementary Table 1 Key resources used in this study.

Reporting Summary

Peer Review File

## Source data

Source Data Fig. 1 Statistical source data.

Source Data Fig. 2 Statistical source data.

Source Data Fig. 3 Statistical source data.

Source Data Fig. 4 Statistical source data.

Source Data Fig. 5 Statistical source data.

Source Data Fig. 6 Statistical source data.

Source Data Fig. 6e Unprocessed full-length western blots.

Source Data Fig. 7 Statistical source data.

Source Data Extended Data Fig. 2 Statistical source data.

Source Data Extended Data Fig. 3 Statistical source data.

Source Data Extended Data Fig. 4 Statistical source data.

Source Data Extended Data Fig. 5 Statistical source data.

Source Data Extended Data Fig. 6 Statistical source data.

Source Data Extended Data Fig. 8 Statistical source data.

Source Data Extended Data Fig. 8f Unprocessed full-length western blots.

Source Data Extended Data Fig. 9 Statistical source data.

## Data Availability

Source and Supplemetary data are provided with this paper. IBEX images are available at https://board.unimib.it/datasets/sytvr3j78g/1. Source data are provided with this paper.
