# Supplementary material for: Microbial adhesion promotes Piezo1 activation to initiate innate immunity
Source: Nat Immunol. 2026 Sep 9;27(11):2225–37. doi: 10.1038/s41590-026-02643-y (PMC13710176; doi:10.1038/s41590-026-02643-y)

Relative to Figure 6e

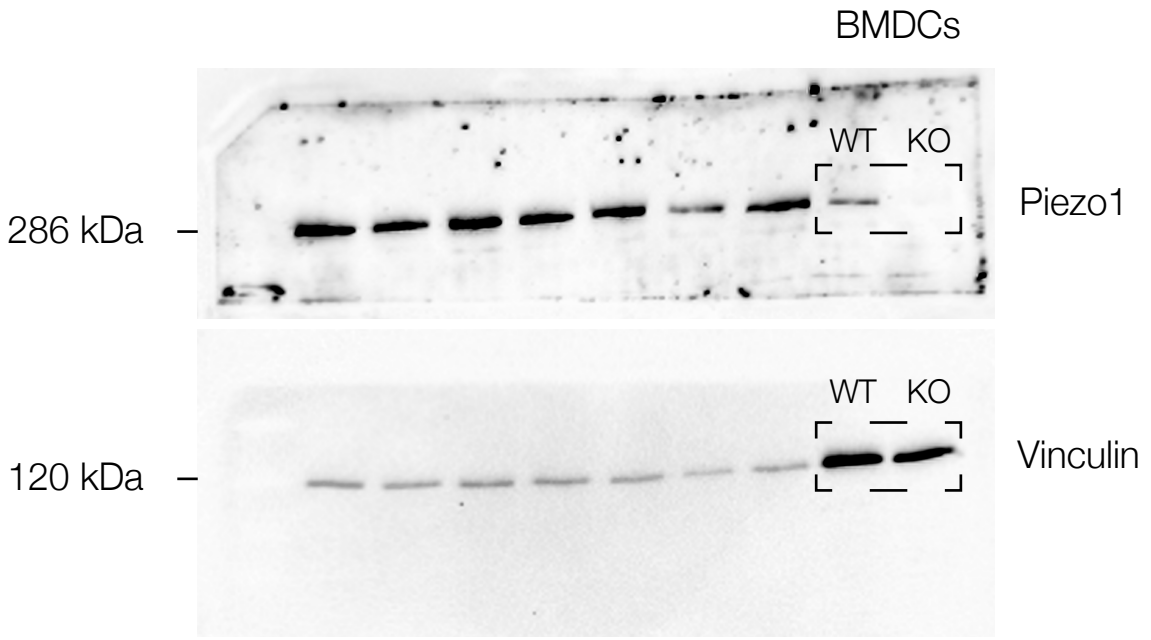

Supplement: Supplementary file 1 [file 41590_2026_2643_MOESM10_ESM.pdf]
