# Supplementary material for: Microbial adhesion promotes Piezo1 activation to initiate innate immunity
Source: Nat Immunol. 2026 Sep 9;27(11):2225–37. doi: 10.1038/s41590-026-02643-y (PMC13710176; doi:10.1038/s41590-026-02643-y)
Supplement: Supplementary file 3 [file 41590_2026_2643_MOESM1_ESM.pdf]

nature immunology

Supplementary information https://doi.org/10.1038/s41590-026-02643-y

Microbial adhesion promotes Piezo1 activation to initiate innate immunity

In the format provided by the authors and unedited

REAGENT or RESOURCE	SOURCE	IDENTIFIER
**Antibodies**		
Normal Goat IgG Control	R & D Systems	Cat AB-108-C
Invitrogen Rat IgG2a kappa Isotype Control (eBR2a), eBioscience™	Thermo Fischer Scientific	Cat 14-4321-85
Invitrogen CXCL1 Monoclonal Antibody (48415)	Thermo Fischer Scientific	Cat MA5-23745
Invitrogen Rat IgG2b kappa Isotype Control (eB149/10H5), eBioscience™	Thermo Fischer Scientific	Cat 14-4031-85
Invitrogen CXCL2 Monoclonal Antibody (40605)	Thermo Fischer Scientific	Cat MA5-23737
Mouse IL-1 alpha /IL-1F1 Antibody	R & D Systems	Cat AF-400-NA
YAP/TAZ (E9M8G) Rabbit mAb #93622	Cell Signaling Technology	Cat 93622S
Ultra-LEAF™ Purified Armenian Hamster IgG Isotype Ctrl Antibody (HTK888)	Biolegend	Cat 400940
Ultra-LEAF™ Purified anti-mouse / rat IL-1β Antibody (B122)	Biolegend	Cat 503514
APC anti-mouse CD80 Antibody (16-10A1)	Biolegend	Cat 104714
PE anti-mouse CD86 Antibody (GL-1)	Biolegend	Cat 105008
Purified anti-mouse CD16/32 Antibody (QA20A66)	Biolegend	Cat 101302
Alexa Fluor® 488 anti-mouse I-Ab Antibody (AF6-120.1)	Biolegend	Cat 116410
Alexa Fluor® 488 anti-mouse CD45 Antibody (30F11)	Biolegend	Cat 103122
PE anti-mouse Ly-6C Antibody (HK1.4)	Biolegend	Cat 128008
InvitrogenGoat anti-Rabbit IgG (H+L) Cross-Adsorbed Secondary Antibody, Alexa Fluor™ 647	Thermo Fisher Scientific	Cat A-21244
PE/Dazzle™ 594 anti-mouse F4/80 Antibody (BM8)	Biolegend	Cat 123146
Purified anti-mouse CD31 Recombinant Antibody (QA20A66)	Biolegend	Cat 109502
PE anti-mouse CD11c Antibody (N418)	Biolegend	Cat 117308
APC anti-mouse CD11c Antibody (N418)	Biolegend	Cat 117310
PerCP/Cyanine5.5 anti-mouse/human CD11b Antibody (M1/70)	Biolegend	Cat 101228
APC/Cyanine7 anti-mouse Ly-6G Antibody (1A8)	Biolegend	Cat 127624
PE/Cyanine7 anti-mouse CD45 Antibody (30-F11)	Biolegend	Cat 103114
Mouse CXCL1/GRO alpha/KC/CINC-1 Alexa Fluor® 647-conjugated Antibody	R & D Systems	Cat IC4532R
BV421 anti-mouse Ly6G (1A8)	BD Horizon	Cat 562737
Pacific Blue anti-mouse CD11b (M1/70)	Biolegend	Cat 101224
BV480 anti-mouse CD3 (17A2)	BD Horizon	Cat 565642
B785 anti-mouse CD45 (30F11)	Biolegend	Cat 103149
FITC anti-mouse I-A/E (2G9)	BD Pharmingen	Cat 553623
PE anti-mouse Ly6C (2G9)	Biolegend	Cat 128008
PE-Fire 810 anti-mouse CD11c (QA18A72)	Biolegend	Cat 161105
Alexa Fluor 488 anti-mouse Pan-Cytokeratin (C-11)	Biolegend	Cat 628608
PE anti-mouse PDGFRa (APA5)	Thermo Fisher Scientific	Cat 12-1401-81
Purified anti-mouse CD31 (MEC 13.3)	Biolegend	Cat 102502
Rabbit IgG Alexa Fluor® 647-conjugated Antibody	R & D Systems	Cat IC1051R
Anti PIEZO1 Polyclonal antibody	Proteintech	Cat 15939-1-AP
Anti PIEZO2 Polyclonal Antibody	Thermo Fisher Scientific	Cat PA5-72975
LIVE/DEAD™ Fixable Scarlet (723) Viability Kit	Thermo Fisher Scientific	Cat L34986
**Bacteria and fungal strains**		
Candida albicans	W. A. Fonzi, GeorgeTown Univeristy (USA)	CAF3-1 strain
Pseudomonas aeruginosa (Schroeter) Migula (ATCC BAA-47-B1)	/	PAO1 strain
Pseudomonas aeruginosa (Schroeter) Migula (ATCC BAA-47-B1)	/	PAO1 strain *PilC-*
Staphylococcus aureus subsp. aureus Rosenbach (ATCC 6538P)	Sigma-Aldrich	ATCC 6538P
**Cell lines**		
GM-CSF-transduced B16 cell line	/	/
GM-CSF-transduced NIH/3T3 cell line (R1 cell line)	https://rupress.org/jem/article/185/2/317/7081/Maturation-Stages-of-Mouse-Dendritic-Cells-in	/
D1 dendritic cell line	https://pubmed.ncbi.nlm.nih.gov/9016880/	/
**Chemicals and peptides**		
LPS E. coli O55:B5 (S-form) TLRpure Sterile Aqueous Solution	Innaxon	Cat IAX-100-013
Recombinant Mouse IL-1α (carrier-free)	Biolegend	Cat 575004
Triton X-100	Sigma-Aldrich	Cat T8787
Diphtheria Toxin	Sigma-Aldrich	Cat D0564
Bovine Serum Albumin	Sigma-Aldrich	Cat B6542
Brefeldin A	Sigma-Aldrich	Cat B6542
Desossiribonuclease I	Sigma-Aldrich	Cat D4263
Fetal Bovine Serum (South America) Sterile Filtered	Euroclone S.p.A.	Cat ECS0180L
Fetal Bovine Serum-United States origin	Euroclone S.p.A.	Cat ECS1104L
L-Glutamine 100X (200 mM)	Euroclone S.p.A.	Cat ECB3000D
Liberase™ TM Research Grade	Roche S.p.A.	Cat 05401127001
Penicillin/Streptomicin 100X	Euroclone S.p.A.	Cat ECB3001D
Iscove's Modified Dulbecco's Medium w/o L-Glutamine w/o Hepes	Euroclone S.p.A.	Cat ECM0192L
RPMI 1640 Medium without L-Glutamine with Phenol Red	Euroclone S.p.A.	Cat ECB9006LX10
TRI reagent®, for DNA, RNA and protein isolation	Sigma-Aldrich	Cat 93289
UltraPure™ 0.5M EDTA, pH 8.0, Invitrogen	Thermo Fisher Scientific	Cat 15575020
Busulfan	Merck	Cat B1170000
Fluo-4, AM cell permeant	Invitrogen	Cat F14201

Probeneacid, water soluble	Invitrogen	Cat P36400
**Critical commercial assays**		
Direct-zol RNA Miniprep Kits	Zymo Research Corp	Cat R2052
in vivo-jetPEI®<br>in vivo DNA & siRNA/miRNA transfection reagent	Polyplus	Cat 101000040
Mouse IL-1 alpha/IL-1F1 DuoSet ELISA	R&D Systems	Cat DY400
Invitrogen Mouse TNF alpha Uncoated ELISA Kit	Thermo Fisher Scientific	Cat 88-7324-88
Leukotriene B4 Express ELISA Kit	Cayman Chemical	Cat 10009292
Goat-on-Rodent HRP-Polymer	BIOCARE Medical	Cat GHP516
EnVision™ FLEXDAB+ Substrate Chromogen System	Dako	Cat GV825
Luna® Universal Probe qPCR Master Mix	New England Biolabs	Cat M3004E
LunaScript® RT SuperMix Kit	New England Biolabs	Cat E3010L
**siRNAs and qPCR probes**		
Ambion™ *In Vivo* Negative Control #1 siRNA, Invitrogen	Thermo Fisher Scientific	Cat 4457287
Ambion™ *In Vivo* Piezo1 siRNA, Invitrogen (s107969)	Thermo Fisher Scientific	Cat 4390771
Mm99999915_g1, *Gapdh* probe	Thermo Fisher Scientific	Cat 4351370
Mm03928990_g1, *Rn18s* probe	Thermo Fisher Scientific	Cat 4351370
Mm00439620_m1, *Il1a* probe	Thermo Fisher Scientific	Cat 4331182
Mm00434228_m1, *Il1b* probe	Thermo Fisher Scientific	Cat 4331182
Mm04207460_m1, *Cxcl1* probe	Thermo Fisher Scientific	Cat 4331182
Mm00436450_m1, *Cxcl2* probe	Thermo Fisher Scientific	Cat 4331182
Mm01241549_m1, *Piezo1* probe	Thermo Fisher Scientific	Cat 4331182
**Software and algorithm**		
FlowJo V10	FlowJo LLC	https://www.flowjo.com
GraphPad Prism 9	GraphPad Software	https://www.graphpad.com/
Halo	Indica Labs	https://indicalab.com/halo/
Fiji ImageJ	/	https://fiji.sc/
BioRender	Biorender Web Tool	https://www.biorender.com/
