# Supplementary material for: Microbial adhesion promotes Piezo1 activation to initiate innate immunity
Source: Nat Immunol. 2026 Sep 9;27(11):2225–37. doi: 10.1038/s41590-026-02643-y (PMC13710176; doi:10.1038/s41590-026-02643-y)
Supplement: Supplementary file 4 [file 41590_2026_2643_MOESM2_ESM.pdf]

nature portfolio

Corresponding author(s): Francesca Granucci, NI-A38954C

Last updated by author(s): Jul 23, 2026

Reporting Summary

Nature Portfolio wishes to improve the reproducibility of the work that we publish. This form provides structure for consistency and transparency in reporting. For further information on Nature Portfolio policies, see our Editorial Policies and the Editorial Policy Checklist.

Statistics

For all statistical analyses, confirm that the following items are present in the figure legend, table legend, main text, or Methods section.

n/a	Confirmed	
☐	☒	The exact sample size (*n*) for each experimental group/condition, given as a discrete number and unit of measurement
☐	☒	A statement on whether measurements were taken from distinct samples or whether the same sample was measured repeatedly
☐	☒	The statistical test(s) used AND whether they are one- or two-sided<br>*Only common tests should be described solely by name; describe more complex techniques in the Methods section.*
☒	☐	A description of all covariates tested
☐	☒	A description of any assumptions or corrections, such as tests of normality and adjustment for multiple comparisons
☐	☒	A full description of the statistical parameters including central tendency (e.g. means) or other basic estimates (e.g. regression coefficient) AND variation (e.g. standard deviation) or associated estimates of uncertainty (e.g. confidence intervals)
☐	☒	For null hypothesis testing, the test statistic (e.g. *F*, *t*, *r*) with confidence intervals, effect sizes, degrees of freedom and *P* value noted<br>*Give P values as exact values whenever suitable.*
☒	☐	For Bayesian analysis, information on the choice of priors and Markov chain Monte Carlo settings
☒	☐	For hierarchical and complex designs, identification of the appropriate level for tests and full reporting of outcomes
☒	☐	Estimates of effect sizes (e.g. Cohen's *d*, Pearson's *r*), indicating how they were calculated

Our web collection on statistics for biologists contains articles on many of the points above.

Software and code

Policy information about availability of computer code

Data collection	Not applicable.
Data analysis	Not applicable.

For manuscripts utilizing custom algorithms or software that are central to the research but not yet described in published literature, software must be made available to editors and reviewers. We strongly encourage code deposition in a community repository (e.g. GitHub). See the Nature Portfolio guidelines for submitting code & software for further information.

Data

Policy information about availability of data

All manuscripts must include a data availability statement. This statement should provide the following information, where applicable:

- Accession codes, unique identifiers, or web links for publicly available datasets
- A description of any restrictions on data availability
- For clinical datasets or third party data, please ensure that the statement adheres to our policy

Source and Supplemetary data are provided with this paper. IBEX images are available at https://board.unimib.it/datasets/sytvr3j78g/1.

Research involving human participants, their data, or biological material

Policy information about studies with human participants or human data. See also policy information about sex, gender (identity/presentation), and sexual orientation and race, ethnicity and racism.

Reporting on sex and gender	Not applicable.
Reporting on race, ethnicity, or other socially relevant groupings	Not applicable.
Population characteristics	Not applicable.
Recruitment	Not applicable.
Ethics oversight	Not applicable.

Note that full information on the approval of the study protocol must also be provided in the manuscript.

Field-specific reporting

Please select the one below that is the best fit for your research. If you are not sure, read the appropriate sections before making your selection.

- [x] Life sciences
- [ ] Behavioural & social sciences
- [ ] Ecological, evolutionary & environmental sciences

For a reference copy of the document with all sections, see nature.com/documents/nr-reporting-summary-flat.pdf

Life sciences study design

All studies must disclose on these points even when the disclosure is negative.

Sample size	No statistical methods were used to predetermine sample sizes. Sample sizes were chosen based on previous experience with these experimental models, while minimizing the number of animals used in accordance with the 3Rs principle of Reduction. The selected sample sizes were considered sufficient because the observed effects were reproducible across independent experiments.
Data exclusions	Data were excluded if tested positive after application of the Robust Regression and Outlier removal Test (ROUT) with GraphPad Prism.
Replication	Experiments with mice were repeated two times to assess robustness and replication. Experiments with cells were replicated three times.
Randomization	Group of mice were matched for sex and age to avoid confounding effect.
Blinding	No blinding.

Reporting for specific materials, systems and methods

We require information from authors about some types of materials, experimental systems and methods used in many studies. Here, indicate whether each material, system or method listed is relevant to your study. If you are not sure if a list item applies to your research, read the appropriate section before selecting a response.

Materials & experimental systems

n/a	Involved in the study	
[ ]	[x]	Antibodies
[ ]	[x]	Eukaryotic cell lines
[x]	[ ]	Palaeontology and archaeology
[ ]	[x]	Animals and other organisms
[x]	[ ]	Clinical data
[x]	[ ]	Dual use research of concern
[x]	[ ]	Plants

Methods

n/a	Involved in the study	
[x]	[ ]	ChIP-seq
[ ]	[x]	Flow cytometry
[x]	[ ]	MRI-based neuroimaging

Antibodies

Antibodies used	Details provided in the manuscript (Table 1). Multiplex imaging with IBEX technique consisted of staining using commercially available antibodies. The following antibodies were used for: antigen(Clone), company (Catalogue number), Conjugation. If not otherwise

stated the antibodies were used at a final concentration of 2 μg/ml.
CD45 (30F11), Biolegend (103122), AF488; CD11c (N418), Biolegend (117310), APC; CD31 (QA20A66), Biolegend, Uncojugated; CD16/32 (93), Biolegend (101302), Uncojugated (1μg/ml); Ly6G (1A8), Biolegend (127624), PE; Pan-Cytokeratin (C-11), Biolegend (628608), AF488 (1:100); PDGFRa (APA5), Thermo Fisher Scientific (12-1401-81), PE (1:100); CD31 (MEC 13.3), Biolegend (102502), Purified (1:50); Piezo1 (polyclonal), Proteintech (15939-1-AP), Purified (1:25); Piezo2 (polyclonal), Thermo Fisher Scientific (PA5-72975), Purified (1:30); Rabbit IgG, R&D Systems (IC1051R), AF647 (1:200). Fluorochrome conjugated polyclonal antibodies (Thermo Fisher Scientific) were used to detect uncojugated primary antibodies.

For flow-cytometry on skin derived cells the following antibodies were used for: antigen (Clone), company (Catalogue number), Conjugation. If not otherwise stated the antibodies were used at a final concentraion of 1 μg/ml.
CD16/32 (93), Biolegend (101302), Uncojugated; CD45 (30F11), Biolegend (103114), PECy7; CD11b (M1/70), Biolegend (101228), PerCP/Cyanine5.5; Ly6G (1A8), Biolegend (127624), APC/Cy7; CD11c (N418), Biolegend (117310), APC; I-Ab (AF6-120.1), Biolegend (116410), AF488; F4/80 (BM8), Biolegend (123146), PE/Dazzle™ 594 (2 μg/ml); Ly6C (HK1.4), Biolegend (128008), PE; CXCL1/GRO alpha/KC/CINC-1 (1174A), R&D Systems (IC4532R), Alexa Fluor® 647 (2.5 μg/ml); Rabbit IgG (60024B), R&D Systems (IC1051R), Alexa Fluor® 647 (2.5 μg/ml); Ly6G (1A8), BD Horizon (562737), BV421 (1:400); CD11b (M1/70), Biolegend (101224), Pac. Blue (1:300); CD3 (17A2), BD Horizon (565642), BV480 (1:200); CD45 (30-F11), Biolegend (103149), BV785 (1:400); I-A/E (2G9), BD Pharmingen (553623), FITC (1:200); Ly6C (HK1.4), Biolegend (128008), PE (1:800); CD11c (QA18A72), Biolegend (161105), PE-Fire 810 (1:300); Pan-Cytokeratin (C-11), Biolegend (628608), AF488 (1:400); PDGFRa (APA5), Thermo Fisher Scientific (12-1401-81), PE (1:400); CD31 (MEC 13.3), Biolegend (102502), Purified (1:200); Piezo1 (polyclonal), Proteintech (15939-1-AP), Purified (1:50); Piezo2 (polyclonal), Thermo Fisher Scientific (PA5-72975), Purified (1:60); Rabbit IgG, R&D Systems (IC1051R), AF647 (1:500); Live/Dead NIR 780, Invitrogen (L34994), 1:6000.

For flow-cytometry of Bone Marrow derived Dendritic Cells (BMDCs) the following antibodies were used for: antigen (Clone), company (Catalogue number), Conjugation. If not otherwise stated the antibodies were used at a final concentraion of 1 μg/ml.
CD16/32 (93), Biolegend (101302), Uncojugated; CD11c (N418), Biolegend (117324), APC/Cy7; I-Ab (AF6-120.1), Biolegend (116410), AF488; CD80 (16-10A1), Biolegend (104714), APC; CD86 (GL-1), Biolegend (105008), PE

For IHC staining of IL-1α in mouse skin, the following antibody was used: Mouse IL-1 alpha /IL-1F1 Antibody (Polyclonal), R&D Systems (AF-400-NA), Uncojugated (15 μg/ml). The signal was detected with DAB staining using Goat-on-Rodent HRP-Polymer (BIOCARE Medical, cat GHP516).

For in vivo neutralization of cytokines/chemokines the following neutralizing antibodies or isotype controls were used: antigen (Clone), company (Catalogue number), Conjugation (Concentration).
Normal Goat IgG Control (Polyclonal), R&D Systems (AB-108-C), Uncojugated (10 μg/ml); Mouse IL-1 alpha /IL-1F1 Antibody (Polyclonal), R&D Systems (AB-108-C), Uncojugated (10 μg/ml); Ultra-LEAF™ Purified Armenian Hamster IgG Isotype Ctrl Antibody (HTK888), Biolegend (400940), Uncojugated (10 μg/ml); Ultra-LEAF™ Purified anti-mouse / rat IL-1β Antibody (B122), Biolegend (503514), Uncojugated (10 μg/ml); Invitrogen Rat IgG2a kappa Isotype Control (eBR2a), eBioscience™ Thermo Fischer Scientific (14-4321-85), Uncojugated (30 μg/mouse); InvitrogenCXCL1 Monoclonal Antibody (48415), Thermo Fischer Scientific (MA5-23745), Uncojugated (30 μg/mouse); Invitrogen Rat IgG2b kappa Isotype Control (eB149/10H5), eBioscience™ Thermo Fischer Scientific (14-4031-859), Uncojugated (30 μg/mouse); Invitrogen CXCL2 Monoclonal Antibody (40605), Thermo Fischer Scientific (MA5-23737), Uncojugated (30 μg/mouse)

Validation

For multiplexed imaging with IBEX technique the fluorochrome directly conjugated antibodies were validated in the pubblication regarding the method, which is already cited in the manuscript. Regarding the uncojugated primary antibodies, their validation was already provided by the manufacturer for immunofluorescence.
For flow-cytometry, the antibodies validation for this technique was already given by the manufacturer.
For IHC of IL-1α we used the Normal Goat IgG Control (Polyclonal), R&D Systems (AB-108-C), Uncojugated (15 μg/ml) to validate the specific staining.
For in vivo neutralization of cytokines/chemokines, the isotyper controls listed above were used as control to verify the effect given by the antibody specificity on neutralization of target cytokine/chemokine.

Eukaryotic cell lines

Policy information about cell lines and Sex and Gender in Research

Cell line source(s)

D1 dendritic cell line (female; 10.1084/jem.185.2.317); primary bone marrow-derived dendritic cells from MyD88-deficient, C57BL/6 and Piezo1/2 fl/fl mice (male and female).

Authentication

no authentication

Mycoplasma contamination

All cell tested were regularly tested for mycoplasma presence and were mycoplasma negative.

Commonly misidentified lines
(See ICLAC register)

Not applicable.

Animals and other research organisms

Policy information about studies involving animals; ARRIVE guidelines recommended for reporting animal research, and Sex and Gender in Research

Laboratory animals	Mice were maintained and bred in a pathogen-free environment at the animal facilities of the University of Milano Bicocca, Milan, Italy. Mice were fed a normal chow diet and housed at 20 ± 2 °C, with 55±10% relative humidity under a 12h light/dark cycle, with lights on at 7:00 a.m. and off at 19:00 p.m.. Males and females aged 6-9 weeks and matched for age and sex were used during the procedures. Littermates of the same genotype and age were randomly assigned to experimental groups. The animal studies were conducted after approval by the Italian Ministry of Health (Approval n. 592/2020-PR) and in accordance with institutional guidelines. WT C57BL/6 mice were supplied by Envigo, Italy. Card9-/- ,Tlr2-/- , and CD11c-CRE mice were purchased from the Jackson Laboratory. Il1r1-/-52 were provided by C. Garlanda (Humanitas Research Institute, Rozzano, Italy), Myd88-/- and Tlr4 -/- mice by S. Akira (IFReC, Japan), CD11c.DOG mice37, where a specific CD11c+ cell ablation can be induced by diphteria toxin (DT) injection by N. Garbi (Institute of Molecular Medicine and Experimental Immunology, Bonn, Germany), Piezo1flox/floxPiezo2flox/flox were provided by L. Catherine (INSERM UMR_S1255, Strasbourg, France). All mutant mouse lines were maintained on C57BL/6 background.
Wild animals	The study did not involved wild animals.
Reporting on sex	Females and males.
Field-collected samples	No field collect samples.
Ethics oversight	Italian Ministry of Health (Approval n. 592/2020-PR).

Note that full information on the approval of the study protocol must also be provided in the manuscript.

Plants

Seed stocks	Not applicable.
Novel plant genotypes	Not applicable.
Authentication	Not applicable.

Flow Cytometry

Plots

Confirm that:

☒ The axis labels state the marker and fluorochrome used (e.g. CD4-FITC).

☒ The axis scales are clearly visible. Include numbers along axes only for bottom left plot of group (a 'group' is an analysis of identical markers).

☒ All plots are contour plots with outliers or pseudocolor plots.

☒ A numerical value for number of cells or percentage (with statistics) is provided.

Methodology

Sample preparation	Briefly, mouse ears were amputated, split into dorsal and ventral halves, and cut into small pieces in digestive medium (RPMI 1640, 5% FBS, L-Glutamine, Penicillin and Streptomycin, Liberase 300 µg/ml, DNase I 50 U/ml). After 90 min. at 37°C, digestion was stopped with pre-chilled complete RPMI medium (RPMI 1640, 10% FBS, L-Glutamine, Penicillin and Streptomycin). The cells were then passed through a 5-ml syringe and filtered through a 70 µM cell strainer (Greiner) to obtain a homogeneous cell suspension. The skin of the footpad was digested with the same protocol, but it was detached from the amputated footpad before digestion.<br>For purity check of differentiated Bone marrow derived Dendritic Cells (BMDCs), DCs were detached by gentle pipetting. For cell-surface marker staining, single-cell skin suspensions or BMDCs were first stained with a viability dye (LIVE/DEAD™ Fixable Scarlet (723) Viability Kit, Thermo Fisher Scientific, USA) for 30 min. at RT to label dead cells. Afterwards, the cells were blocked with blocking solution (PBS, 0,5% BSA, 2mM EDTA, α-CD16/32) for 20 min. at RT. Antibody mix was directly added to the blocking solution, and samples were incubated for 20 min. at 4°C. Finally, the cells were fixed with paraformaldehyde (BD Cytofix Fixation Buffer, cat 554655, BD Biosciences, USA) for 15 min. at 4°C, washed three times in PBS and transferred into flow cytometry tubes.<br>For the CXCL-1 staining, the skin tissue and the cell suspension were handled adding to all employed solutions s Brefeldin A (5µg/ml) to block the secretion of CXCL-1. After surface staining, as described above, the samples were fixed with

eBioscience™ Foxp3 / Transcription Factor Fixation/Permeabilization Concentrate and Diluent (Thermo Fisher Scientific, USA) overnight at 4°C. The following day, the cells were stained with α-CXCL-1 AF647 antibody or control isotype, and samples were acquired on the same day.
For Piezo1 and Piezo2 detection, single-cell skin suspensions were first incubated with LIVE/DEAD Fixable Near-IR 780 viability dye (Thermo Fisher Scientific) for 15 min at room temperature. After washing with FACS buffer, the cells were blocked for 20 min at 4 °C with Fc receptor blocking solution containing anti-CD16/32 antibody. Unconjugated rabbit anti-mouse primary antibodies against Piezo1 or Piezo2 were directly added to the blocking solution, and the cells were incubated for 30 min at 37 °C. The cells were subsequently washed with FACS buffer, centrifuged (500g, 5 min, 4 °C) and stained with Alexa Fluor 647-conjugated goat anti-rabbit secondary antibody for 20 min at 4 °C. After washing with FACS buffer, the cells were centrifuged again (500g, 5 min, 4 °C) and stained with the following fluorochrome-conjugated surface antibodies: Ly6G, CD45, F4/80, PDGFRα, CD31, CD11c, CD3. Following surface staining, the cells were fixed and permeabilized using the Foxp3/ Transcription Factor Fixation/Permeabilization kit (Thermo Fisher Scientific) according to the manufacturer's instructions and subsequently stained with anti-pan-cytokeratin antibody to identify keratinocytes.
For Ca2+ measurements atotal of 5×105 cells (D1 cells or BMDCs) were loaded with 3 µM Fluo-4 acetoxymethyl ester (Fluo-4 AM; Invitrogen) and incubated for 30 min. at 37°C in complete medium supplemented with probenecid (Invitrogen). Cells were then washed, centrifugated and resuspended in complete medium containing probenecid and equilibrated at 37°C before acquisition. Ca2+ fluxes were subsequently analyzed by flow cytometry. Cells were stimulated with either P. aeruginosa or S. aureus (1.7×10^8 CFU), C. albicans (5x10^4 cells), or Yoda1 (40 µM; Merck Life Science). Where indicated, cells were pretreated with GsMTx-4 (5 µM; Selleckchem) during the loading with Fluo-4 AM, which was also added in the resuspension medium.

Instrument

Cytoflex S (Beckman Coulter), Cytek Aurora 3L (V/B/R) (Cytek Biosciences).

Software

FlowJo Software v10 (Becton Dickinson, USA).

Cell population abundance

Out of live cells CD45+, the subsets analysed had a frequency of at least 2%.

Gating strategy

Enclosed in Supplementary Figure 1., Supplementary Figure 4., Supplementary Figure 7. Total cells (CD45+ and CD45-) were gated on FSC and SSC to exclude debris. Then single cells were gated using FSC-A and FSC-W. After excluding doublets and dead cells (L/D Scarlet negative cells), neutrophils were identified as CD45+CD11bhighLy6Ghigh cells, dendritic cells as CD45 +CD11c+I-Abhigh.
For Piezo1/2 expression analysis in the skin singlets were first selected based on FSC-A and FSC-H, followed by exclusion of dead cells using live/dead staining. CD45+ immune cells, CD45- stromal cells, CD31+ vessels and PDGFRα+ fibroblasts were identified. Within the CD45+ compartment, neutrophils were gated as Ly6G+ cells, T cells as CD3+ cells, and CD11c+ cells were distinguished from other immune cells.

☒ Tick this box to confirm that a figure exemplifying the gating strategy is provided in the Supplementary Information.
