# Supplementary material for: Microbial adhesion promotes Piezo1 activation to initiate innate immunity
Source: Nat Immunol. 2026 Sep 9;27(11):2225–37. doi: 10.1038/s41590-026-02643-y (PMC13710176; doi:10.1038/s41590-026-02643-y)
Supplement: Supplementary file 5 [file 41590_2026_2643_MOESM3_ESM.pdf]

nature portfolio

# Peer Review File

# Microbial adhesion promotes Piezo1 activation to initiate innate immunity

Corresponding Author: Professor Francesca Granucci

**This file contains all editorial decision letters in order by version, followed by all author rebuttals in order by version.**

Version 0:

Reviewer comments:

Reviewer #1

(Remarks to the Author)
In this manuscript the authors address how innate immune cell migration to sites of infection is initially facilitated and if divergent mechanism exist after the infection becomes more established. They describe a 2-tier system in which they believe mechanosensation plays a key and undocumented role in initiating neutrophil recruitment to infected tissue which is eventually replaced by the production DAMP associated signals and chemokines.
While dissecting how cells initially migrate to tissues and how that differs as infection time courses progress is of great interest to the field, the main novelty in this study revolves around that mechanosensation may be a central mechanism driving initial recruitment of neutrophils. Unfortunately, however, the work on mechanosensation is rather weak and only limited to figure 5 of the paper. Several unaddressed caveats relate to this work limiting overall robustness in the findings and enthusiasm. These are summarized into 3 key concerns outlined below:
1) What mechanical pressure is sensed?
Throughout the paper the authors do not define what mechanical pressure is sensed in their infection model. The authors would need to define what exact pressure is sensed in the tissue. Is this pressure form tissue stretch, form edema associated with infection etc. Without some explanation it is hard to conclude pressure or force and Piezo1modualtion is what triggers this, as pharmacological activation with Yoda or inhibition with the spider venom GsTmx4 can have varying and dramatic off target effects when used in vivo.
2) What cells actually sense this pressure?
The authors do not define which cells are expressing Piezo1 in this model, and also do not definitively show which cell type is required to initiate this process. The use of Yap nuclear translocation is not definitive evidence of Piezo1 activity in endothelial cells or fibroblast and many factors can cause this process independent of mechanosensation. Use the imperfect model of Yoda1 and GsTmx4, is this lost during infection? More compelling data is required to validate this claim, which is central to the authors conclusions.
3) Why are Piezo1-flox animals not used to validate the pharmacological work?
A simple solution for several of my concerns would be to identify the cell type(s) expressing Piezo1 in this model, and cross the publicly available Piezo1 flox animals. If the authors are correct in that Piezo1 and mechanical force are required to initiate Neutrohil migration, these animals will be needed to fully validate the authors claims. While the use of siRNA against Piezo1 is informative, this will also absolutely target myeloid cells, and this could affect myeloid depend migration of neutrophils to the tissue in mechanisms already discovered. Finding the stromal cell that senses force to cause PMN migration via Piezo1 would greatly increase this papers interest and constitute a novel and interesting finding.

Minor Point
I found the introduction interesting and thought provoking, but it did not introduce the topic of mechanosensation in a meaningful way. As this is in the paper's title, I think it could benefit from some more airtime in the introduction.

Reviewer #2

(Remarks to the Author)
The authors investigated neutrophil recruitment at microbial infection sites, focusing on the immune response in the skin following infections with fungi, Gram-positive bacteria, and Gram-negative bacteria. The authors further examined the role of key pathways, particularly MyD88 and IL1R signaling, in regulating early and late neutrophil responses to infection. They

also investigated the regulation of neutrophil recruitment via CXCL1/2.Finally, they proposed that PIEZO1 mediates early neutrophil recruitment through LTB4-IL1 axis. This manuscript addresses an important topic concerning mechanoreceptors and neutrophil function. However, the current study appears to be preliminary. A major concern is the reliance on 'non-specific' reagents, such as inhibitors and antibodies, without additional supporting data. This limitation weakens the authors' conclusions, particularly given recent controversies regarding the role of PIEZO1 in neutrophil functon(see Mukhopadhyay et al., 2024; Knaus et al., 2024; Wu et al., 2024). To strengthen their findings, the authors should provide further supporting evidence, such as data from genetic mouse models. Finally, the title of the manuscript could be a little misleading, as the mechanoreceptor-related data were not the major focus.

Decision Letter:

5th Dec 2024

Dear Dr. Granucci,

We have now finished reviewing your manuscript entitled "Mechanoreceptors initiate innate immunity in response to microbial infections", reference number NI-A38954.

Although the editors thought that the manuscript was interesting enough to send out for in-depth review, the reviewers were not in favor of publishing the paper in Nature Immunology because there is not enough evidence to support the conclusions. In particular, both reviewers highlight the need of genetic mouse models to show evidence to support your findings, and reviewer #1 thinks it is important that you show the stromal cell that senses force to cause PMN migration via Piezo1. We are therefore returning the reviews to you with the hope that you find them useful when you prepare the paper for another journal.

Should you find yourself able to thoroughly address those concerns, please let me know. If the novelty of the paper has not been compromised in the interim, it is possible that you could resubmit a new paper. At the time we would reevaluate the revised manuscript at the editorial level and decide whether the revised manuscript would be sent back for peer review.

If you decide to resubmit, please include a “Response to referees” detailing, point-by-point, how you addressed each referee comment. This response will be sent back to the referees along with the revised manuscript. Please include a revised version of any required reporting checklist. It will be available to referees (and, potentially, statisticians) to aid in their evaluation if the manuscript goes back for peer review. A revised checklist is essential for re-review of the paper. The Reporting summary can be found here: https://www.nature.com/documents/nr-reporting-summary.pdf

Although we cannot offer to publish your manuscript, I suggest that you consider Nature Communications as a suitable venue for this work. To transfer your manuscript, please use our <a href="Link Redacted">manuscript transfer portal</a>. You will not have to re-supply manuscript metadata and files, unless you wish to make modifications. For more information, please see our <a href="http://www.nature.com/authors/author_resources/transfer_manuscripts.html?WT.mc_id=EMI_NPG_1511_AUTHORTRANSF&WT.ec_id=AUTHOR">manuscript transfer FAQ</a> page.

We realize that this is disappointing. I hope that you continue to consider Nature Immunology for your results most significant for the immunology community and wish you well in your future investigations.

Sincerely,
Paula Jauregui

---
Paula Jauregui, PhD
Senior Editor
Nature Immunology

Reviewers' comments:

Reviewer #1 (Remarks to the Author):

In this manuscript the authors address how innate immune cell migration to sites of infection is initially facilitated and if divergent mechanism exist after the infection becomes more established. They describe a 2-tier system in which they believe mechanosensation plays a key and undocumented role in initiating neutrophil recruitment to infected tissue which is eventually replaced by the production DAMP associated signals and chemokines.
While dissecting how cells initially migrate to tissues and how that differs as infection time courses progress is of great interest to the field, the main novelty in this study revolves around that mechanosensation may be a central mechanism driving initial recruitment of neutrophils. Unfortunately, however, the work on mechanosensation is rather weak and only limited to figure 5 of the paper. Several unaddressed caveats relate to this work limiting overall robustness in the findings and enthusiasm. These are summarized into 3 key concerns outlined below:
1) What mechanical pressure is sensed?

Throughout the paper the authors do not define what mechanical pressure is sensed in their infection model. The authors would need to define what exact pressure is sensed in the tissue. Is this pressure form tissue stretch, form edema associated with infection etc. Without some explanation it is hard to conclude pressure or force and Piezo1modualtion is what triggers this, as pharmacological activation with Yoda or inhibition with the spider venom GsTmx4 can have varying and dramatic off target effects when used in vivo.
2) What cells actually sense this pressure?
The authors do not define which cells are expressing Piezo1 in this model, and also do not definitively show which cell type is required to initiate this process. The use of Yap nuclear translocation is not definitive evidence of Piezo1 activity in endothelial cells or fibroblast and many factors can cause this process independent of mechanosensation. Use the imperfect model of Yoda1 and GsTmx4, is this lost during infection? More compelling data is required to validate this claim, which is central to the authors conclusions.
3) Why are Piezo1-flox animals not used to validate the pharmacological work?
A simple solution for several of my concerns would be to identify the cell type(s) expressing Piezo1 in this model, and cross the publicly available Piezo1 flox animals. If the authors are correct in that Piezo1 and mechanical force are required to initiate Neutrohil migration, these animals will be needed to fully validate the authors claims. While the use of siRNA against Piezo1 is informative, this will also absolutely target myeloid cells, and this could affect myeloid depend migration of neutrophils to the tissue in mechanisms already discovered. Finding the stromal cell that senses force to cause PMN migration via Piezo1 would greatly increase this papers interest and constitute a novel and interesting finding.

Minor Point
I found the introduction interesting and thought provoking, but it did not introduce the topic of mechanosensation in a meaningful way. As this is in the paper's title, I think it could benefit from some more airtime in the introduction.

Reviewer #2 (Remarks to the Author):

The authors investigated neutrophil recruitment at microbial infection sites, focusing on the immune response in the skin following infections with fungi, Gram-positive bacteria, and Gram-negative bacteria. The authors further examined the role of key pathways, particularly MyD88 and IL1R signaling, in regulating early and late neutrophil responses to infection. They also investigated the regulation of neutrophil recruitment via CXCL1/2.Finally, they proposed that PIEZO1 mediates early neutrophil recruitment through LTB4-IL1 axis. This manuscript addresses an important topic concerning mechanoreceptors and neutrophil function. However, the current study appears to be preliminary. A major concern is the reliance on 'non-specific' reagents, such as inhibitors and antibodies, without additional supporting data. This limitation weakens the authors' conclusions, particularly given recent controversies regarding the role of PIEZO1 in neutrophil functon(see Mukhopadhyay et al., 2024; Knaus et al., 2024; Wu et al., 2024). To strengthen their findings, the authors should provide further supporting evidence, such as data from genetic mouse models. Finally, the title of the manuscript could be a little misleading, as the mechanoreceptor-related data were not the major focus.

Version 1:

Reviewer comments:

Reviewer #1

(Remarks to the Author)
The authors have substantially revised the manuscript and added experiments addressing (i) the physical trigger for early neutrophil recruitment and (ii) the relevant Piezo1-expressing cell type. The revised model, microbial adhesion providing mechanical input sensed by Piezo1 in CD11c⁺ myeloid cells to drive LTB4-dependent neutrophil recruitment prior to PAMP/MyD88-driven chemokines, is intriguing and the new data move the work forward. It is also a notable departure from the earlier submission that emphasized a stromal source of mechanotransduction; this shift is not explicitly discussed in the rebuttal.
However, several issues still limit interpretability, particularly the genetic strategy, the directness of the mechanosensation claim, and cell-type resolution.
1. Genetic deletion strategy (Piezo1 vs Piezo2; delivery method).
The manuscript uses local, lentiviral "kiss and run" Cre in Piezo1/2 double-floxed mice rather than conditional Piezo1-only ablation. This choice is not justified and complicates interpretation because Piezo2 is a distinct mechanosensor. Were experiments first attempted in Piezo1^fl/fl and no phenotype observed, prompting the double knockout? Please (a) provide a clear rationale for the double flox, and (b) include Piezo1-only conditional deletion using standard drivers (for example, CD11c-Cre, Zbtb46-Cre, and/or Cx3cr1-CreER) despite their imperfections. Multiple orthogonal genetic approaches are preferable to a single bespoke system with its own caveats. Notably, the manuscript relies on CD11c.DTR depletion to support conclusions, yet this model can affect neutrophils in unexpected ways (PMID: 22367054). If Cre specificity concerns preclude conventional lines, it is inconsistent to accept CD11c.DTR caveats but reject conditional Cre caveats. As written, the title's claim that Piezo1 is the link between mechanosensation and early PMN migration is not yet warranted in vivo.
2. Mechanosensitive cell type identification.
Figures 5e and 5f are suggestive but not definitive that dermal CD11c⁺ cells are the highest Piezo1 expressers. Please sort major dermal populations and perform qPCR (and, if possible, protein) to compare Piezo1 and Piezo2 expression directly. Use the same imaging or antibody pipeline to verify knockdown or knockout within CD11c⁺ cells after siRNA and local lenti-Cre. Demonstrating on-target loss in CD11c⁺ cells would alleviate concerns about delivery specificity. The data in

Supplementary Figure 7 are not comprehensive: Piezo1 is reported in macrophages, neurons, epithelia or keratinocytes, and, controversially, neutrophils; quantifying only DCs, endothelium, and fibroblasts is insufficient to claim DC-specific targeting by a broadly infective lentivector. Please replace histograms with quantitative summaries and statistical tests of expression and knockout across relevant populations.
3. Do adhesion mutants abrogate mechanosensation?
The pilC- and fnbA/B- experiments are compelling but do not, by themselves, show failure to activate Piezo1. Please add Ca2+ flux readouts (genetically encoded or dye based) during microbe to cell contact, showing: wild type microbes trigger Piezo1-dependent Ca2+ in wild type cells (lost with Piezo1 knockout), whereas adhesion mutants do not. This distinguishes mechanosensory failure from pleiotropic effects of the mutation on virulence, growth, or secretome. For example, does bacteria-free supernatant or heat killed WT bacteria activate the pathway? If so, this may not necessarily be via adhesion of live bacteria inducing membrane stretch. While, unlikely, perhaps another factor may activate the Piezo1 channel as has been controversy suggested by others (see Piezo in epithelial cells as a RNA sensor from the microbiome). Before concluding that, Piezo1 is responding to microbial adhesion, direct downstream Piezo1 Ca2+ signaling will be required and is straightforward and tractable to test.
Also, in Figure 6f the differential TNFa requirement (Piezo1 dependent for Staphylococcus aureus but not Pseudomonas aeruginosa) needs clarification. Why would Piezo1 be required for what is a Myd88 dependent TNFa production in this context?
Overall. The study is potentially important, but the dual Piezo1/2 knockout, lack of Piezo1-only conditional genetics, and absence of direct mechanotransduction readouts keep key claims from being fully supported.
Minor Points:
1. The authors state:
"Upon adhesion, these microbes stretch the host cell membrane, exerting nanonewton mechanical forces that can activate Piezo channels45."
In the cited work, it is reasonable to conclude that microbes can stretch the membrane, but it is not established that Piezo channels are sensitive to this specific interaction. Please refine the citation logic and language. Since it is the authors' novel hypothesis that this interaction activates Piezo channels, direct Ca2+ imaging should be added to test it.
2. Use of ns and non significant p values.
In some cases the authors report a numeric p value for a non significant result (for example, Figure 6b) and in others only "ns." Please report the actual p value consistently in all cases. In Figure 6b, although non significant, the trend appears underpowered; additional replicates would likely yield significance while retaining the qualitative defect. Similarly, in Figure 6d, only three replicates were used; more biological replicates would likely achieve significance. Consistent reporting will help readers interpret these effects.

Reviewer #2

(Remarks to the Author)
The authors have revised their manuscript, but several key concerns from the initial review remain unaddressed. First, the authors use mutant bacteria to suggest that adhesion generates mechanical force. However, this constitutes circumstantial rather than direct evidence. Secondly, they use imaging data to suggest that dendritic cells are the primary expressers of Piezo1; however, the presented images are unconvincing. The data lack sufficient detail and do not clearly explain how expression was quantified. Third, it is surprising that the authors chose not to use the CD11c-Cre mouse model based on a single prior report suggesting a potential effect of CD11c on neutrophil maturation. This rationale seems inconsistent, especially considering that the authors used CD11c-DOG mice to deplete dendritic cells. If CD11c expression truly affects neutrophils, as they suggest, this concern would equally apply to their current depletion strategy, potentially undermining their own data.

Decision Letter:

7th Nov 2025

Dear Professor Granucci,

Your Article, "Microbial adhesion promotes Piezo1 activation to initiate innate immunity" has now been seen by 2 referees. You will see from their comments copied below that while they find your work of considerable potential interest, they have raised quite substantial concerns that must be addressed. In light of these comments, we cannot accept the manuscript for publication, but would be very interested in considering a revised version that addresses these serious concerns.

We hope you will find the referees' comments useful as you decide how to proceed. If you wish to submit a substantially revised manuscript, please bear in mind that we will be reluctant to approach the referees again in the absence of major revisions.

We think that hte revision plan that you sent us is reasonable, although we think that your work will be strengthen using multiple orthogonal genetic approaches since the evidence will be more robust that with a single bespoke system with its own caveats.

If you choose to revise your manuscript taking into account all reviewer and editor comments, please highlight all changes in the manuscript text file.

We are committed to providing a fair and constructive peer-review process. Do not hesitate to contact us if there are specific requests from the reviewers that you believe are technically impossible or unlikely to yield a meaningful outcome.

If revising your manuscript:

* Include a “Response to referees” document detailing, point-by-point, how you addressed each referee comment. If no action was taken to address a point, you must provide a compelling argument. This response will be sent back to the referees along with the revised manuscript.

* If you have not done so already please begin to revise your manuscript so that it conforms to our Article format instructions at http://www.nature.com/ni/authors/index.html. Refer also to any guidelines provided in this letter.

* Include a revised version of any required reporting checklist. It will be available to referees (and, potentially, statisticians) to aid in their evaluation if the manuscript goes back for peer review. A revised checklist is essential for re-review of the paper.

The Reporting Summary can be found here:
https://www.nature.com/documents/nr-reporting-summary.pdf

When submitting the revised version of your manuscript, please pay close attention to our
href="https://www.nature.com/nature-portfolio/editorial-policies/image-integrity">Digital Image Integrity Guidelines.</a> and to the following points below:

-- that unprocessed scans are clearly labelled and match the gels and western blots presented in figures.
-- that control panels for gels and western blots are appropriately described as loading on sample processing controls
-- all images in the paper are checked for duplication of panels and for splicing of gel lanes.

Extended Data figures and tables are online-only (appearing in the online PDF and full-text HTML version of the paper), peer-reviewed display items that provide essential background to the Article but are not included in the printed version of the paper due to space constraints or being of interest only to a few specialists. A maximum of ten Extended Data display items (figures and tables) is typically permitted. When re-submitting your manuscript, please ensure that any supplementary figures and tables that are more critical to the manuscript’s conclusions are converted to Extended data to increase these data’s visibility.

Finally, please ensure that you retain unprocessed data and metadata files after publication, ideally archiving data in perpetuity, as these may be requested during the peer review and production process or after publication if any issues arise.

You may use the link below to submit your revised manuscript and related files:

Link Redacted

**Note:** This URL links to your confidential home page and associated information about manuscripts you may have submitted, or that you are reviewing for us. If you wish to forward this email to co-authors, please delete the link to your homepage.

If you wish to submit a suitably revised manuscript we would hope to receive it within 6 months. If you cannot send it within this time, please let us know. We will be happy to consider your revision so long as nothing similar has been accepted for publication at Nature Immunology or published elsewhere.

Nature Immunology is committed to improving transparency in authorship. As part of our efforts in this direction, we are now requesting that all authors identified as ‘corresponding author’ on published papers create and link their Open Researcher and Contributor Identifier (ORCID) with their account on the Manuscript Tracking System (MTS), prior to acceptance. ORCID helps the scientific community achieve unambiguous attribution of all scholarly contributions. You can create and link your ORCID from the home page of the MTS by clicking on ‘Modify my Springer Nature account’. For more information please visit please visit <a href="http://www.springernature.com/orcid">www.springernature.com/orcid</a>.

Please do not hesitate to contact me if you have any questions or would like to discuss the required revisions further.

Thank you for the opportunity to review your work.

Sincerely,
Paula

---
Paula Jauregui, PhD
Senior Editor
Nature Immunology

Referee expertise:

Referee #1: PIEZO and sensors of inflammation

Referee #2: Neutrophils

Reviewers' Comments:

Reviewer #1 (Remarks to the Author):

The authors have substantially revised the manuscript and added experiments addressing (i) the physical trigger for early neutrophil recruitment and (ii) the relevant Piezo1-expressing cell type. The revised model, microbial adhesion providing mechanical input sensed by Piezo1 in CD11c⁺ myeloid cells to drive LTB4-dependent neutrophil recruitment prior to PAMP/MyD88-driven chemokines, is intriguing and the new data move the work forward. It is also a notable departure from the earlier submission that emphasized a stromal source of mechanotransduction; this shift is not explicitly discussed in the rebuttal.

However, several issues still limit interpretability, particularly the genetic strategy, the directness of the mechanosensation claim, and cell-type resolution.

1. Genetic deletion strategy (Piezo1 vs Piezo2; delivery method).

The manuscript uses local, lentiviral "kiss and run" Cre in Piezo1/2 double-floxed mice rather than conditional Piezo1-only ablation. This choice is not justified and complicates interpretation because Piezo2 is a distinct mechanosensor. Were experiments first attempted in Piezo1^fl/fl and no phenotype observed, prompting the double knockout? Please (a) provide a clear rationale for the double flox, and (b) include Piezo1-only conditional deletion using standard drivers (for example, CD11c-Cre, Zbtb46-Cre, and/or Cx3cr1-CreER) despite their imperfections. Multiple orthogonal genetic approaches are preferable to a single bespoke system with its own caveats. Notably, the manuscript relies on CD11c.DTR depletion to support conclusions, yet this model can affect neutrophils in unexpected ways (PMID: 22367054). If Cre specificity concerns preclude conventional lines, it is inconsistent to accept CD11c.DTR caveats but reject conditional Cre caveats. As written, the title's claim that Piezo1 is the link between mechanosensation and early PMN migration is not yet warranted in vivo.

2. Mechanosensitive cell type identification.

Figures 5e and 5f are suggestive but not definitive that dermal CD11c⁺ cells are the highest Piezo1 expressers. Please sort major dermal populations and perform qPCR (and, if possible, protein) to compare Piezo1 and Piezo2 expression directly. Use the same imaging or antibody pipeline to verify knockdown or knockout within CD11c⁺ cells after siRNA and local lenti-Cre. Demonstrating on-target loss in CD11c⁺ cells would alleviate concerns about delivery specificity. The data in Supplementary Figure 7 are not comprehensive: Piezo1 is reported in macrophages, neurons, epithelia or keratinocytes, and, controversially, neutrophils; quantifying only DCs, endothelium, and fibroblasts is insufficient to claim DC-specific targeting by a broadly infective lentivector. Please replace histograms with quantitative summaries and statistical tests of expression and knockout across relevant populations.

3. Do adhesion mutants abrogate mechanosensation?

The pilC- and fnbA/B- experiments are compelling but do not, by themselves, show failure to activate Piezo1. Please add Ca2+ flux readouts (genetically encoded or dye based) during microbe to cell contact, showing: wild type microbes trigger Piezo1-dependent Ca2+ in wild type cells (lost with Piezo1 knockout), whereas adhesion mutants do not. This distinguishes mechanosensory failure from pleiotropic effects of the mutation on virulence, growth, or secretome. For example, does bacteria-free supernatant or heat killed WT bacteria activate the pathway? If so, this may not necessarily be via adhesion of live bacteria inducing membrane stretch. While, unlikely, perhaps another factor may activate the Piezo1 channel as has been controversy suggested by others (see Piezo in epithelial cells as a RNA sensor from the microbiome). Before concluding that, Piezo1 is responding to microbial adhesion, direct downstream Piezo1 Ca2+ signaling will be required and is straightforward and tractable to test.

Also, in Figure 6f the differential TNFa requirement (Piezo1 dependent for Staphylococcus aureus but not Pseudomonas aeruginosa) needs clarification. Why would Piezo1 be required for what is a Myd88 dependent TNFa production in this context?

Overall. The study is potentially important, but the dual Piezo1/2 knockout, lack of Piezo1-only conditional genetics, and absence of direct mechanotransduction readouts keep key claims from being fully supported.

Minor Points:

1. The authors state:

"Upon adhesion, these microbes stretch the host cell membrane, exerting nanonewton mechanical forces that can activate Piezo channels45."

In the cited work, it is reasonable to conclude that microbes can stretch the membrane, but it is not established that Piezo channels are sensitive to this specific interaction. Please refine the citation logic and language. Since it is the authors' novel hypothesis that this interaction activates Piezo channels, direct Ca2+ imaging should be added to test it.

2. Use of ns and non significant p values.

In some cases the authors report a numeric p value for a non significant result (for example, Figure 6b) and in others only "ns." Please report the actual p value consistently in all cases. In Figure 6b, although non significant, the trend appears underpowered; additional replicates would likely yield significance while retaining the qualitative defect. Similarly, in Figure 6d, only three replicates were used; more biological replicates would likely achieve significance. Consistent reporting will help readers interpret these effects.

Reviewer #2 (Remarks to the Author):

The authors have revised their manuscript, but several key concerns from the initial review remain unaddressed. First, the authors use mutant bacteria to suggest that adhesion generates mechanical force. However, this constitutes circumstantial rather than direct evidence. Secondly, they use imaging data to suggest that dendritic cells are the primary expressers of Piezo1; however, the presented images are unconvincing. The data lack sufficient detail and do not clearly explain how expression was quantified. Third, it is surprising that the authors chose not to use the CD11c-Cre mouse model based on a single prior report suggesting a potential effect of CD11c on neutrophil maturation. This rationale seems inconsistent, especially considering that the authors used CD11c-DOG mice to deplete dendritic cells. If CD11c expression truly affects neutrophils, as they suggest, this concern would equally apply to their current depletion strategy, potentially undermining their own data.

Version 2:

Reviewer comments:

Reviewer #1

(Remarks to the Author)
The authors have addressed the major technical concerns I raised, and I now feel their conclusions are justified by the extensive new experiment presented.

Reviewer #2

(Remarks to the Author)
The authors have addressed majority of the original comments, but it is important to note that the central claim of the manuscript has shifted substantially: the cellular source of LTB4 has moved from stromal cells to CD11c+ myeloid cells.

The cell-specific in vivo evidence for Piezo1 remains incomplete (Fig. 5j,k). The cleanest genetic experiment, CD11c-Cre × Piezo1fl, is shown only for C. albicans and only for neutrophil counts. It should be extended to S. aureus and P. aeruginosa, and should include the proximal readouts (LTB4, and ideally IL-1α and CXCL1), so that the genotype is tied to the proposed mechanism rather than to the terminal phenotype alone (increased neutrophil counts).

A more fundamental concern is the attribution of LTB4 to CD11c+ cells, given that LTB4 is itself a principal driver of neutrophil swarming and is produced by neutrophils in an autocrine and paracrine relay (Lämmermann et al., 2013). Once the first neutrophils arrive, they amplify the LTB4 pool, so the tissue LTB4 measured in Figs 4 and 5 reflects a mixture of CD11c+-derived and neutrophil-derived lipid. Any intervention that lowers neutrophil numbers, including CD11c depletion, Piezo1 loss, or Bestatin treatment, will also reduce neutrophil-derived LTB4 as a secondary effect.

The observed reductions therefore cannot be purely attributed to loss of an initiating CD11c+ source, and with the current data it is difficult to establish the two-step recruitment model definitively. Doing so would require cell-restricted manipulation of LTB4 synthesis to CD11c+ cells versus neutrophils, together with LTB4 measurements at early timepoints before substantial neutrophil influx.

Decision Letter:

24th Jun 2026

Dear Professor Granucci,

Your Article, "Microbial adhesion promotes Piezo1 activation to initiate innate immunity" has now been seen by 2 referees. You will see from their comments below that while they find your work of interest, some important points are raised. We are interested in the possibility of publishing your study in Nature Immunology, but would like to consider your response to these concerns in the form of a revised manuscript before we make a final decision on publication.

We think that your plan for revision is reasonable and that the additional CD11c-Cre × Piezo1^fl/fl experiments with P. aeruginosa and S. aureus are not needed.

We therefore invite you to revise your manuscript taking into account all reviewer and editor comments. Please highlight all changes in the manuscript text file in Microsoft Word format.

We are committed to providing a fair and constructive peer-review process. Do not hesitate to contact us if there are specific requests from the reviewers that you believe are technically impossible or unlikely to yield a meaningful outcome.

When revising your manuscript:

* Include a “Response to referees” document detailing, point-by-point, how you addressed each referee comment. If no action was taken to address a point, you must provide a compelling argument. This response will be sent back to the referees along with the revised manuscript.

* If you have not done so already please begin to revise your manuscript so that it conforms to our Article format instructions at http://www.nature.com/ni/authors/index.html. Refer also to any guidelines provided in this letter.

* Please include a revised version of any required reporting checklist. It will be available to referees to aid in their evaluation of the manuscript goes back for peer review. They are available here:

Reporting summary:
https://www.nature.com/documents/nr-reporting-summary.pdf

When submitting the revised version of your manuscript, please pay close attention to our href="https://www.nature.com/nature-portfolio/editorial-policies/image-integrity">Digital Image Integrity Guidelines.</a> and to the following points below:

-- that unprocessed scans are clearly labelled and match the gels and western blots presented in figures.
-- that control panels for gels and western blots are appropriately described as loading on sample processing controls
-- all images in the paper are checked for duplication of panels and for splicing of gel lanes.

Please note, Extended Data figures and tables are online-only (appearing in the online PDF and full-text HTML version of the paper), peer-reviewed display items that provide essential background to the Article but are not included in the printed version of the paper due to space constraints or being of interest only to a few specialists. A maximum of ten Extended Data display items (figures and tables) is typically permitted. When re-submitting your manuscript, please ensure that any supplementary figures and tables that are more critical to the manuscript's conclusions are converted to Extended data to increase these data's visibility.

Finally, please ensure that you retain unprocessed data and metadata files after publication, ideally archiving data in perpetuity, as these may be requested during the peer review and production process or after publication if any issues arise.

Please use the link below to submit your revised manuscript and related files:

Link Redacted

**Note:** This URL links to your confidential home page and associated information about manuscripts you may have submitted, or that you are reviewing for us. If you wish to forward this email to co-authors, please delete the link to your homepage.

We hope to receive your revised manuscript within two weeks. If you cannot send it within this time, please let us know. We will be happy to consider your revision so long as nothing similar has been accepted for publication at Nature Immunology or published elsewhere.

Please do not hesitate to contact me if you have any questions or would like to discuss these revisions further.

Nature Immunology is committed to improving transparency in authorship. As part of our efforts in this direction, we are now requesting that all authors identified as 'corresponding author' on published papers create and link their Open Researcher and Contributor Identifier (ORCID) with their account on the Manuscript Tracking System (MTS), prior to acceptance. ORCID helps the scientific community achieve unambiguous attribution of all scholarly contributions. You can create and link your ORCID from the home page of the MTS by clicking on 'Modify my Springer Nature account'. For more information please visit please visit <a href="http://www.springernature.com/orcid">www.springernature.com/orcid</a>.

We look forward to seeing the revised manuscript and thank you for the opportunity to review your work.

Sincerely,
Paula

---
Paula Jauregui, PhD
Senior Editor
Nature Immunology

Reviewers' Comments:

Reviewer #1 (Remarks to the Author):

The authors have addressed the major technical concerns I raised, and I now feel their conclusions are justified by the extensive new experiment presented.

Reviewer #2 (Remarks to the Author):

The authors have addressed majority of the original comments, but it is important to note that the central claim of the manuscript has shifted substantially: the cellular source of LTB4 has moved from stromal cells to CD11c+ myeloid cells.

The cell-specific in vivo evidence for Piezo1 remains incomplete (Fig. 5j,k). The cleanest genetic experiment, CD11c-Cre × Piezo1fl, is shown only for C. albicans and only for neutrophil counts. It should be extended to S. aureus and P. aeruginosa, and should include the proximal readouts (LTB4, and ideally IL-1α and CXCL1), so that the genotype is tied to the proposed mechanism rather than to the terminal phenotype alone (increased neutrophil counts).

A more fundamental concern is the attribution of LTB4 to CD11c+ cells, given that LTB4 is itself a principal driver of neutrophil swarming and is produced by neutrophils in an autocrine and paracrine relay (Lämmermann et al., 2013). Once the first neutrophils arrive, they amplify the LTB4 pool, so the tissue LTB4 measured in Figs 4 and 5 reflects a mixture of CD11c+-derived and neutrophil-derived lipid. Any intervention that lowers neutrophil numbers, including CD11c depletion, Piezo1 loss, or Bestatin treatment, will also reduce neutrophil-derived LTB4 as a secondary effect.

The observed reductions therefore cannot be purely attributed to loss of an initiating CD11c+ source, and with the current data it is difficult to establish the two-step recruitment model definitively. Doing so would require cell-restricted manipulation of LTB4 synthesis to CD11c+ cells versus neutrophils, together with LTB4 measurements at early timepoints before substantial neutrophil influx.

Version 3:

Reviewer comments:

Reviewer #2

(Remarks to the Author)
The authors should be commended for their considerable efforts during the revision process. I appreciate their thoughtful and thorough responses to my comments, as well as the improvements made to the manuscript. All my comments have now been adequately addressed.

Decision Letter:

Our ref: NI-A38954C

8th Jul 2026

Dear Dr. Granucci,

Thank you for submitting your revised manuscript "Microbial adhesion promotes Piezo1 activation to initiate innate immunity" (NI-A38954C). It has now been seen by the original referees and their comments are below. The reviewers find that the paper has improved in revision, and therefore we'll be happy in principle to publish it in Nature Immunology, pending minor revisions to satisfy the referees' final requests and to comply with our editorial and formatting guidelines.

We will now perform detailed checks on your paper and will send you a checklist detailing our editorial and formatting requirements in a couple of weeks. Please do not upload the final materials and make any revisions until you receive this additional information from us.

If you had not uploaded a Word file for the current version of the manuscript, we will need one before beginning the editing process; please email that to immunology@us.nature.com at your earliest convenience.

Thank you again for your interest in Nature Immunology Please do not hesitate to contact me if you have any questions.

Sincerely,
Paula

---
Paula Jauregui, PhD
Senior Editor
Nature Immunology

Reviewer #2 (Remarks to the Author):

The authors should be commended for their considerable efforts during the revision process. I appreciate their thoughtful and thorough responses to my comments, as well as the improvements made to the manuscript. All my comments have now been adequately addressed.

*INTERNAL USE ONLY* AIP modules required: ["Life Science"]

**Open Access** This Peer Review File is licensed under a Creative Commons Attribution 4.0 International License, which permits use, sharing, adaptation, distribution and reproduction in any medium or format, as long as you give appropriate credit to the original author(s) and the source, provide a link to the Creative Commons license, and indicate if changes were made.
In cases where reviewers are anonymous, credit should be given to 'Anonymous Referee' and the source.
The images or other third party material in this Peer Review File are included in the article's Creative Commons license, unless indicated otherwise in a credit line to the material. If material is not included in the article's Creative Commons license and your intended use is not permitted by statutory regulation or exceeds the permitted use, you will need to obtain permission directly from the copyright holder.
To view a copy of this license, visit https://creativecommons.org/licenses/by/4.0/

**Response to Reviewers**

We would like to thank the reviewers for the thorough and constructive evaluation of our manuscript, which has allowed us to strengthen the conclusions of our study.

Indeed, all their major and minor concerns and comments have been carefully tackled. We performed a series of additional experiments to solve the two main issues raised by the reviewers, as to the identity of the cells triggering the recruitment of neutrophils to the infection site and the involvement of mechanosensation and Piezo in this process. Furthermore, we addressed all comments either experimentally or textually.

As a result, the manuscript has been largely revised, and all changes and additions are clearly marked in the main text file are indicated in the with tracked edits.

We hope that the extensive revisions and new data satisfy the reviewers and clearly underscore the robustness and novelty of our findings. Below, we provide a detailed point-by-point response to each of the reviewers' comments.

## Reviewer #1

*In this manuscript the authors address how innate immune cell migration to sites of infection is initially facilitated and if divergent mechanism exist after the infection becomes more established. They describe a 2-tier system in which they believe mechanosensation plays a key and undocumented role in initiating neutrophil recruitment to infected tissue which is eventually replaced by the production DAMP associated signals and chemokines.*
*While dissecting how cells initially migrate to tissues and how that differs as infection time courses progress is of great interest to the field, the main novelty in this study revolves around that mechanosensation may be a central mechanism driving initial recruitment of neutrophils.*

**Authors:** We thank the reviewer for appreciating both the impact and the novelty of the two-tiered mechanism of neutrophil recruitment.

*Unfortunately, however, the work on mechanosensation is rather weak and only limited to figure 5 of the paper. Several unaddressed caveats relate to this work limiting overall robustness in the findings and enthusiasm.*

**Authors:** We appreciate the remark that providing more robust evidence for the mechanism regulating the first wave of neutrophil recruitment would overall strengthen the findings. Thus, we have included several new experiments that specifically address this point. The new Figure 5 includes ten panels with pharmacological and genetic *in vivo* and *in vitro* data, employing orthogonal approaches (imaging, siRNA, and mouse knockout models) to firmly establish the role of Piezo1 and $LTB_4$. However, the main objective of the manuscript is unravelling the molecular mechanisms underlying the activation of innate immunity in response to microbial stimuli. We show that Piezo1 and $LTB_4$, which we identified in this work as a Piezo1 downstream effector molecule, are crucial for initiating neutrophil recruitment. This provides important evidence that early danger-driven signals, rather than PAMPs, trigger this response.

*1) What mechanical pressure is sensed?*
*Throughout the paper the authors do not define what mechanical pressure is sensed in their infection model. The authors would need to define what exact pressure is sensed in the tissue. Is this pressure form tissue stretch, form edema associated with infection etc. Without some explanation it is hard to conclude pressure or force and Piezo1modualtion is what triggers this, as pharmacological activation with Yoda or inhibition with the spider venom GsTmx4 can have varying and dramatic off target effects when used in vivo.*

**Authors:** As noted by the reviewer, there was no clear mechanistic explanation of how mechanical stress arises during infection in the manuscript. The revised version directly addresses this loose end through new experimentation

It is well accepted that pathogen adhesion to host cells and tissues is one of the early events during infections. This process requires specific molecular machineries, type IV pili in *Pseudomonas aeruginosa* (Hospenthal MK, et al. A comprehensive guide to pilus biogenesis in Gram-negative bacteria. Nat Rev Microbiol. 2017;15:365–379. doi: 10.1038/nrmicro.2017.40), fibronectin-binding proteins in *Staphylococcus aureus (*Ulrich Schwarz-Linek et al. The molecular basis of fibronectin-mediated bacterial adherence to host cells. 2004 Mol Microbiol, DOI: 10.1111/j.1365-2958.2004.04027.x), and a variety of adhesins in *Candida albicans*.

On adhesion, these microbes can exert mechanical force on the host's cell membrane, leading to stretching forces that have been previously characterised (N. Biais, et al. Cooperative retraction of bundled type IV pili enables nanonewton force generation, PLoS Biol., vol. 6, p. e87, avr. 2008, doi: 10.1371/journal.pbio.0060087.).
On these grounds, we hypothesised that microbial adhesion to host tissues could generate the mechanical stimulus promoting Piezo1 activation. To test this possibility, we performed the following experiments:

1) We infected the skin with *P. aeruginosa* mutants knocked out for *pilC* () or *S. aureus* mutants knocked out for *fnb-A/B*. The former cannot form pili and are thus unable to adhere to the host's cell membrane, whereas the latter lack fibronectin-binding adhesins mediating binding and internalization. We found that both mutant strains failed to induce early $LTB_4$ production and neutrophil recruitment (new Figure 6), regardless of their PAMPs. The requirement for adhesion in $LTB_4$ production was also shown *in vitro* using BMDCs (Figure 6 c-g).

2) We confirmed that Piezo1 is required for $LTB_4$ production and neutrophil recruitment by using *in vivo* and *in vitro* a conditional lentiviral-based depletion model to delete Piezo1 in myeloid cells (new Figure 5j, new Figure 6d and g)).

3) We found that $LTB_4$ can be produced in an adhesion- and Piezo1-dependent manner, but independently of MyD88, even in the presence of PAMPs (new Figure 6c). This indicates that microbial adhesion provides a mechanosensory input sufficient to trigger the production of key mediators of innate immune responses.

Together, these results show that pathogen adhesion-mediated mechanical stress is the primary activator of Piezo1.

*2) What cells actually sense this pressure?*
*The authors do not define which cells are expressing Piezo1 in this model, and also do not definitively show which cell type is required to initiate this process. The use of Yap nuclear translocation is not definitive evidence of Piezo1 activity in endothelial cells or fibroblast and many factors can cause this process independent of mechanosensation. Use the imperfect model of Yoda1 and GsTmx4, is this lost during infection? More compelling data is required to validate this claim, which is central to the authors conclusions.*

**Authors:** We thank the reviewer for highlighting this issue. We agree that Yap translocation is not a specific readout of Piezo activation. Thus, we did not further utilize it. To address the reviewer's question, we instead capitalized on our novel finding that $LTB_4$ is a downstream effector of Piezo1 (see answer above).

We performed multiplex imaging to identify the cells that express Piezo1 in the skin and found that while most of the cells in the skin express Piezo1 (new Figure 5e), only $CD11c^+$ cells express high levels of both Piezo1 and Alox5, one of the enzymes required for the production of $LTB_4$ (new Figure 5f). Accordingly, eliminating $CD11c^+$ cells abrogated not only neutrophil recruitment but also the production of $LTB_4$ upon pathogen infection (new Figure 5 g,h). DT administration eliminated most $Piezo^+Alox5^+CD11c^+$ cells (Fig.5f, middle panel) and the remaining $CD11c^+$ population did not contain $Piezo1^{high}Alox5^{high}$ cells.

As suggested, we also used Yoda1 *in vivo* to see whether it was sufficient to recruit neutrophils. In line with our previous *in vitro* data showing $LTB_4$ production by DCs treated with Yoda1 (old Figure

5D, new Supplementary Figure S6c), we observed an $LTB_4$-dependent recruitment of neutrophils (new Figure 5d).

Collectively, these data indicate that $CD11c^+$ cells, mainly DCs, are the cell type that initiates the process.

3)*Why are Piezo1-flox animals not used to validate the pharmacological work? A simple solution for several of my concerns would be to identify the cell type(s) expressing Piezo1 in this model, and cross the publicly available Piezo1 flox animals. If the authors are correct in that Piezo1 and mechanical force are required to initiate Neutrohil migration, these animals will be needed to fully validate the authors claims. While the use of siRNA against Piezo1 is informative, this will also absolutely target myeloid cells, and this could affect myeloid depend migration of neutrophils to the tissue in mechanisms already discovered. Finding the stromal cell that senses force to cause PMN migration via Piezo1 would greatly increase this papers interest and constitute a novel and interesting finding.*

**Authors:** We thank the reviewer for this valuable suggestion, which we have followed to corroborate that Piezo plays a key role in the early response to microbial infections.
We have identified $CD11c^+$ cells as the Piezo1-expressing cell type in mouse skin that produces $LTB_4$, which in turn initiates innate immunity, through the experiments described above. We could not rely on widely used Cre mouse lines, such as LysM-Cre mice or CD11c-Cre mice because they both affect Piezo1 expression in neutrophils. Indeed, the LysM-Cre mice are known to drive Cre expression not only in DCs and macrophages, but also in monocytes and granulocytes. Similarly, CD11c-Cre mice are not suitable either, as CD11c is expressed not only by DCs and macrophages but also in neutrophils (Lifei Hou, Koichi Yuki et al. CD11c regulates neutrophil maturation. Blood Advances 28 October 2022. https://doi.org/10.1182/ bloodadvances.2022007719). Reliance on these conventional Cre/lox-based deletion systems to obtain cell type-specific Piezo1 knockouts would confound the interpretation of these experiments, as optimal neutrophil migration could require Piezo1 (Mukhopadhyay et al., trans-Endothelial neutrophil migration activates bactericidal function via Piezo1 mechanosensing. 2024 Jan 9;57(1):52-67.e10. doi: 10.1016/j.immuni.2023.11.007).
Hence, we had to devise an alternative way to achieve the genetic ablation of *Piezo1* in macrophages and DCs *in vivo*. Leveraging the prominent endocytic and patrolling activities of these cells, we decided to intradermally inject Piezo1,2 floxed mice with lentiviral particles enabling kiss-and-run expression of Cre.
This treatment reduced Piezo1 levels in dermal $CD11c^+$ cells, but not endothelial cells or fibroblasts, at the site of injection, as shown in the figure below and in the new Supplementary Figure 7g. Of note, this lentiviral Cre system also allows local genetic manipulation of $CD11c^+$ cells while sparing global populations.
By generating mice with local Piezo1 knockout in $CD11c^+$ cells via lentiviral Cre delivery at the site of microbial infection, it has been possible to prove that Piezo1-driven $LTB_4$ production by myeloid cells initiates neutrophil recruitment. The new Figure 5j shows that in these mice both the production of $LTB_4$ and neutrophil recruitment are markedly impaired.
These data confirm that resident $CD11c^+$ cells are responsible for the initiation of innate responses through the activation of Piezo1 and $LTB_4$ production.

Importantly, we also shed light on the underlying mechanism by showing that microbial adhesion acts as a previously unrecognized signal enabling myeloid cells to sense microbes and initiate innate immunity via Piezo1 activation.

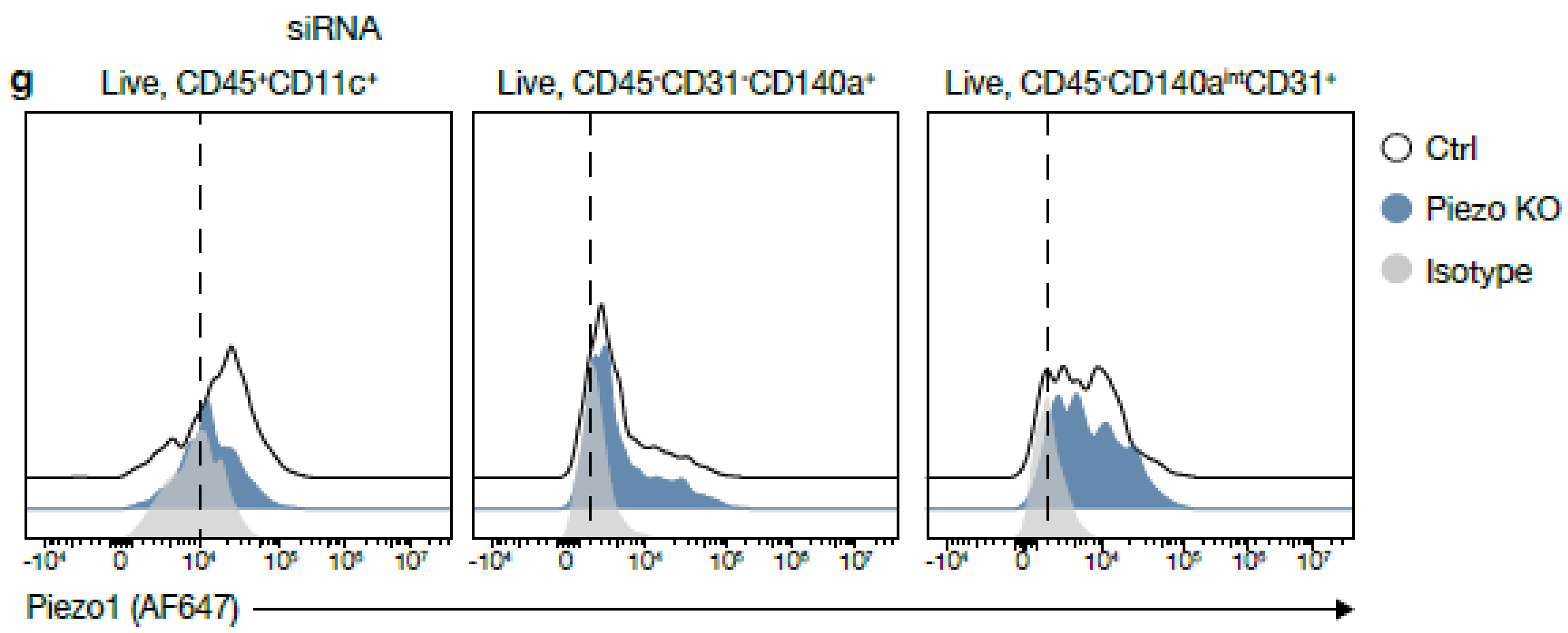


<u>*Piezo1 is deleted in CD11c*$^{+}$ *cells, but not in fibroblasts or endothelial cells*</u>

*Minor Point*
*I found the introduction interesting and thought provoking, but it did not introduce the topic of mechanosensation in a meaningful way. As this is in the paper's title, I think it could benefit from some more airtime in the introduction.*

**Authors:** As discussed above, the purpose of the manuscript was to identify the mechanism initiating innate immunity. Accordingly, we have changed the title to highlight the take-home message more clearly.
The involvement of mechanosensation in the activation of innate immunity, independent PAMPs, emerged as a discovery rather than something anticipated based from the literature. For this reason, we believe that discussing mechanosensation in immunity in the introduction would constitute a logical detour and reduce the readability of that section. Nevertheless, we have ensured that the key studies on mechanotransduction and bacterial adhesion are cited in the Results section, where they are directly relevant to our findings.

## Reviewer #2

*The authors investigated neutrophil recruitment at microbial infection sites, focusing on the immune response in the skin following infections with fungi, Gram-positive bacteria, and Gram-negative bacteria. The authors further examined the role of key pathways, particularly MyD88 and IL1R signaling, in regulating early and late neutrophil responses to infection. They also investigated the regulation of neutrophil recruitment via CXCL1/2.Finally, they proposed that PIEZO1 mediates early neutrophil recruitment through LTB4-IL1 axis. This manuscript addresses an important topic concerning mechanoreceptors and neutrophil function.*

**Authors:** We thank the reviewer for their thoughtful evaluation and recognition of the importance of our study.

*However, the current study appears to be preliminary. A major concern is the reliance on 'non-specific' reagents, such as inhibitors and antibodies, without additional supporting data. This limitation weakens the authors' conclusions, particularly given recent controversies regarding the role of PIEZO1 in neutrophil functon(see Mukhopadhyay et al., 2024; Knaus et al., 2024; Wu et al., 2024). To strengthen their findings, the authors should provide further supporting evidence, such as data from genetic mouse models.*

**Authors:** We thank the reviewer for pointing out the need for more robust evidence supporting the role of Piezo1 in neutrophil recruitment. In the revised version of the manuscript, we have confirmed the role of Piezo1-$LTB_4$-IL-1α axis using multiple complementary approaches.
First, we found that microbial adhesion acts as the mechanism promoting Piezo1 activation for the subsequent production of $LTB_4$, which we identified as a hitherto unknown major Piezo1 effector molecule.
It is well accepted that pathogen adhesion to host cells and tissues is one of the early events during infections.
This process requires specific molecular machineries, type IV pili in *Pseudomonas aeruginosa* (Hospenthal MK, et al. A comprehensive guide to pilus biogenesis in Gram-negative bacteria. Nat Rev Microbiol. 2017;15:365–379. doi: 10.1038/nrmicro.2017.40), fibronectin-binding proteins in *Staphylococcus aureus (*Ulrich Schwarz-Linek et al. The molecular basis of fibronectin-mediated bacterial adherence to host cells. 2004 Mol Microbiol, DOI: 10.1111/j.1365-2958.2004.04027.x), and a variety of adhesins in *Candida albicans*.
On adhesion, these microbes can exert mechanical force on the host's cell membrane, leading to stretching forces that have been previously characterised (N. Biais, et al. Cooperative retraction of bundled type IV pili enables nanonewton force generation, PLoS Biol., vol. 6, p. e87, avr. 2008, doi: 10.1371/journal.pbio.0060087.).
By infecting mice with microorganisms defective in pili (*P. aeruginosa pilC*$^{-}$) or fibronectin-binding proteins *(S. aureus fnb-A/B*$^{-}$), which are essential for adhesion, we found that these strains failed to induce early $LTB_4$ release and neutrophil recruitment, despite the presence of PAMPs. Consistently, BMDCs infected with the adhesion-deficient mutants did not produce $LTB_4$, whereas wild-type microbes triggered a Piezo1-dependent, MyD88-independent release. These results jointly demonstrate that microbial adhesion is required for Piezo1 activation, $LTB_4$ production, and neutrophil recruitment (new Fig. 6).

Second, we used multiplexed imaging to identify the cells that express Piezo1 in the skin. We found that most of the cells of the skin express Piezo1, but only the CD11c$^{+}$ cells express Piezo1 at high levels and are double positive for Piezo1 and Alox5, one of the enzymes required for the biosynthesis of $LTB_4$ (new Figure 5e). Accordingly, the elimination of CD11c$^{+}$ cells abrogated not only neutrophil

recruitment but also the production of $LTB_4$, the effector molecule downstream of Piezo1, upon pathogen infection (new Figure 5g,h).

We also injected Yoda1 *in vivo* to see whether this was sufficient to induce the recruitment of neutrophils. According to our previous data showing that Yoda1 stimulates $LTB_4$ production by DCs *in vitro* (old Figure 5D, new Supplementary Figure 6c), neutrophil recruitment to the site of Yoda1 injection occurred in a $LTB_4$-dependent manner (new Figure 5d).

Collectively, these data indicate that $CD11c^+$ cells, mainly DCs, are the cell type that initiates the innate immune response.

Third, we used a genetic model to further demonstrate the role of Piezo1.
We have identified $CD11c^+$ cells as the Piezo1-expressing cell type in mouse skin that produces $LTB_4$, which in turn initiates innate immunity, through the experiments described above. We could not rely on widely used Cre mouse lines, such as LysM-Cre mice or CD11c-Cre mice because they both affect Piezo1 expression in neutrophils. Indeed, the LysM-Cre mice are known to drive Cre expression not only in DCs and macrophages, but also in monocytes and granulocytes. Similarly, CD11c-Cre mice are not suitable either, as CD11c is expressed not only by DCs and macrophages but also in neutrophils (Lifei Hou, Koichi Yuki et al. CD11c regulates neutrophil maturation. Blood Advances 28 October 2022. https://doi.org/10.1182/ bloodadvances.2022007719). Reliance on these conventional Cre/lox-based deletion systems to obtain cell type-specific Piezo1 knockouts would confound the interpretation of these experiments, as optimal neutrophil migration could require Piezo1 (Mukhopadhyay et al., trans-Endothelial neutrophil migration activates bactericidal function via Piezo1 mechanosensing. 2024 Jan 9;57(1):52-67.e10. doi: 10.1016/j.immuni.2023.11.007).
Hence, we had to devise an alternative way to achieve the genetic ablation of *Piezo1* in macrophages and DCs *in vivo*. Leveraging the prominent endocytic and patrolling activities of these cells, we decided to intradermally inject Piezo1,2 floxed mice with lentiviral particles enabling kiss-and-run expression of Cre.
This treatment reduced Piezo1 levels in dermal $CD11c^+$ cells, but not endothelial cells or fibroblasts, at the site of injection, as shown in the figure below and in the new Supplementary Figure 7g. Of note, this lentiviral Cre system also allows local genetic manipulation of $CD11c^+$ cells while sparing global populations.
By generating mice with local Piezo1 knockout in $CD11c^+$ cells via lentiviral Cre delivery at the site of microbial infection, it has been possible to prove that Piezo1-driven $LTB_4$ production by myeloid cells initiates neutrophil recruitment. The new Figure 5j shows that in these mice both the production of $LTB_4$ and neutrophil recruitment are impaired.
These data confirm that resident $CD11c^+$ cells are responsible for the initiation of innate responses through the activation of Piezo1 and $LTB_4$ production.

Importantly, we also shed light on the underlying mechanism by showing that microbial adhesion acts as a previously unrecognized signal enabling myeloid cells to sense microbes and initiate innate immunity via Piezo1 activation.

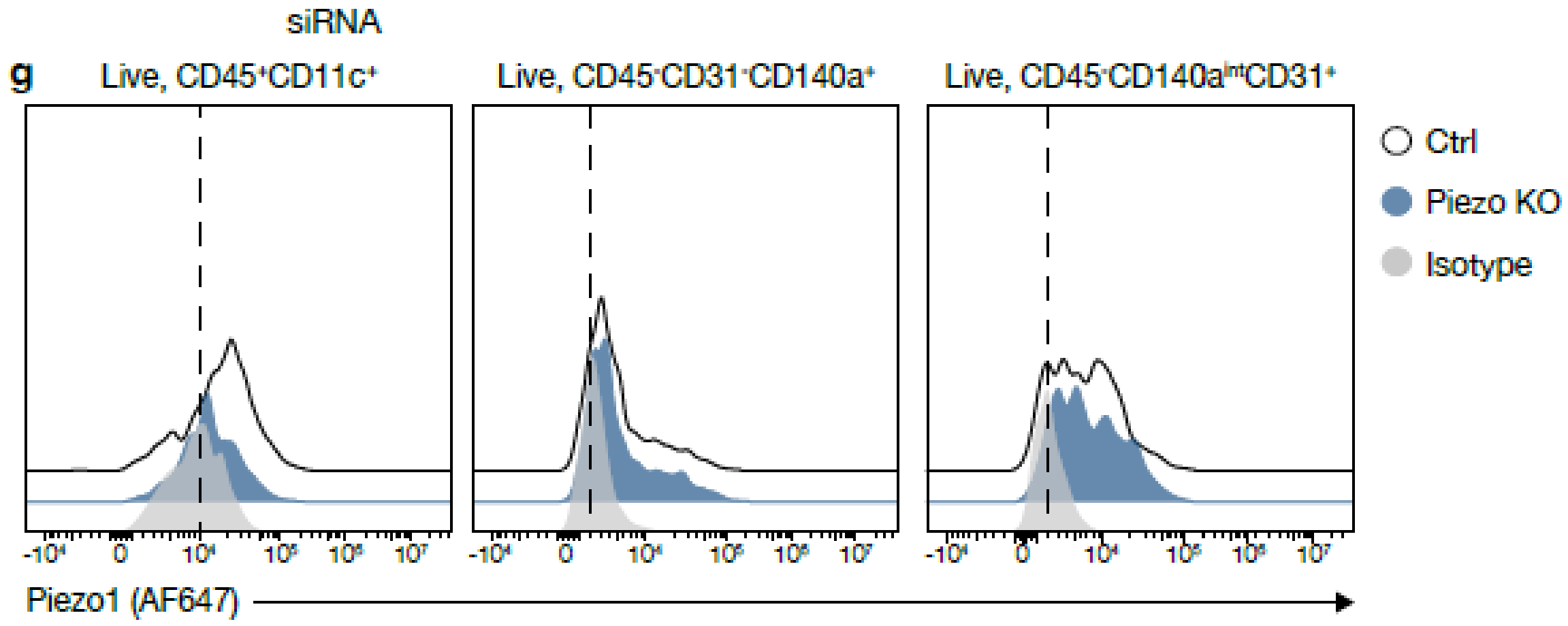


*Piezo1 is deleted in CD11c$^+$ cells, but not in fibroblasts or endothelial cells*

*Finally, the title of the manuscript could be a little misleading, as the mechanoreceptor-related data were not the major focus.*

**Authors:** We are grateful for this suggestion and have modified the title in the revised manuscript to more clearly highlight the major focus of our study.

We are indebted to the reviewers for their insightful comments, which helped us further improve our manuscript.
Our point-by-point response to their criticisms is below.

**Reviewer 1**

***The authors have substantially revised the manuscript and added experiments addressing (i) the physical trigger for early neutrophil recruitment and (ii) the relevant Piezo1-expressing cell type. The revised model, microbial adhesion providing mechanical input sensed by Piezo1 in CD11c⁺ myeloid cells to drive LTB4-dependent neutrophil recruitment prior to PAMP/MyD88-driven chemokines, is intriguing and the new data move the work forward. It is also a notable departure from the earlier submission that emphasized a stromal source of mechanotransduction; this shift is not explicitly discussed in the rebuttal.***

We thank the reviewer for this positive evaluation of the revised manuscript. Thanks to their previous comment on the use of YAP/TAZ as readout of Piezo1 activation, we performed additional experiments, which were added to the revised manuscript, showing that $CD11c^+$ myeloid cells are the main population expressing Piezo1 and Alox5 and mediating neutrophil recruitment through $LTB_4$ production.

1. ***Genetic deletion strategy (Piezo1 vs Piezo2; delivery method).***

***The manuscript uses local, lentiviral "kiss and run" Cre in Piezo1/2 double-floxed mice rather than conditional Piezo1-only ablation. This choice is not justified and complicates interpretation because Piezo2 is a distinct mechanosensor. Were experiments first attempted in Piezo1^fl/fl and no phenotype observed, prompting the double knockout? Please (a) provide a clear rationale for the double flox, and ....***

We used Piezo1/2 double-floxed mice because these animals were readily available and provided a reliable model to specifically interrogate the role of Piezo1 in our system. According to the literature, Piezo2 expression is restricted to Merkel cells in the epidermis and to dorsal root ganglion neurons (PMID: 24717433; PMID: 25471886), neither of which is relevant to our study. We have now performed cytofluorimetric analyses of skin cells to verify the expression of Piezo1 and Piezo2. The results are included in the present version of the manuscript (new Fig. 5e and Supplementary Fig. S7). We found that Piezo1 is expressed at high levels in $CD11c^+$ cells and keratinocytes, and moderately in the endothelium. By contrast, Piezo2 expression is restricted to keratinocytes (new Fig. 5e and

Supplementary Fig. S7). Therefore, in the dermis - the tissue layer that constitutes the site of infection - only Piezo1 is expressed, primarily by CD11c$^+$ dendritic cells and macrophages.

To exclude the possible involvement of non-immune cells expressing Piezo1 in the recruitment process, we generated bone marrow chimeras in which recipient DOG mice were transplanted with bone marrow cells from Piezo1/2$^{fl/fl}$ mice. Eighteen weeks after transplantation, mice were treated with DT, both i.v. and s.c., to eliminate any possible residual DOG DCs (see new Supplementary Fig. 9a). Analysis of chimerism showed that more than 90% of hematopoietic cells were of donor origin (new Supplementary Fig. S9b). The mice were then treated s.c. with lenti-Cre, and a clear ablation of Piezo1 in CD11c$^+$ cells was observed in the skin (see new Supplementary Fig. S9c). In this model, we found that $LTB_4$ production and neutrophil recruitment were significantly reduced following microbial infection (new Fig. 5j).

***(b) include Piezo1-only conditional deletion using standard drivers (for example, CD11c-Cre, Zbtb46-Cre, and/or Cx3cr1-CreER) despite their imperfections. Multiple orthogonal genetic approaches are preferable to a single bespoke system with its own caveats.***

We generated a genetic model by backcrossing Piezo1/2$^{fl/fl}$ mice with CD11c-Cre mice. We selected Piezo1$^{fl/fl}$ x CD11c-Cre F2 mice and infected them with *C. albicans*. In this setting too, deletion of Piezo1 in CD11c$^+$ cells strongly reduced neutrophil recruitment (new Fig. 5k and Supplementary Fig. S9d).
The convergence of the results obtained with these two models strengthens our conclusions.

***Notably, the manuscript relies on CD11c.DTR depletion to support conclusions, yet this model can affect neutrophils in unexpected ways (PMID: 22367054). If Cre specificity concerns preclude conventional lines, it is inconsistent to accept CD11c.DTR caveats but reject conditional Cre caveats. As written, the title's claim that Piezo1 is the link between mechanosensation and early PMN migration is not yet warranted in vivo.***

We did not use CD11c.DTR mice but CD11c.DOG mice, which are less prone to DT-induced neutrophilia. We followed a well-established protocol in our laboratory (Zanoni et al. JCI, 2012; Zanoni et al. Cell Reports 2013; Colombo et al. iScience, 2022): 16 ng of DT per gram body weight is administered, and neutrophil counts are routinely assessed 48 hours later as a control. The cited study reported neutrophilia at 24 hours after DT administration in CD11c.DOG mice treated with 40 ng/g bw. In contrast, we do not observe neutrophilia, as shown in the figure below. This setup allows us to selectively deplete CD11c$^+$ DCs and macrophages without affecting neutrophil population.

Even if neutrophilia were present, the observed differences would still hold and, in fact, be even more

significant, given the increased number of circulating neutrophils prone to extravasate, as described in the study cited by the reviewer.

In any case, we have also generated Piezo1$^{fl/fl}$ x CD11c-Cre F2 mice and observed impaired neutrophil recruitment upon infection (new Fig. 5k and new Supplementary Fig. S9d).

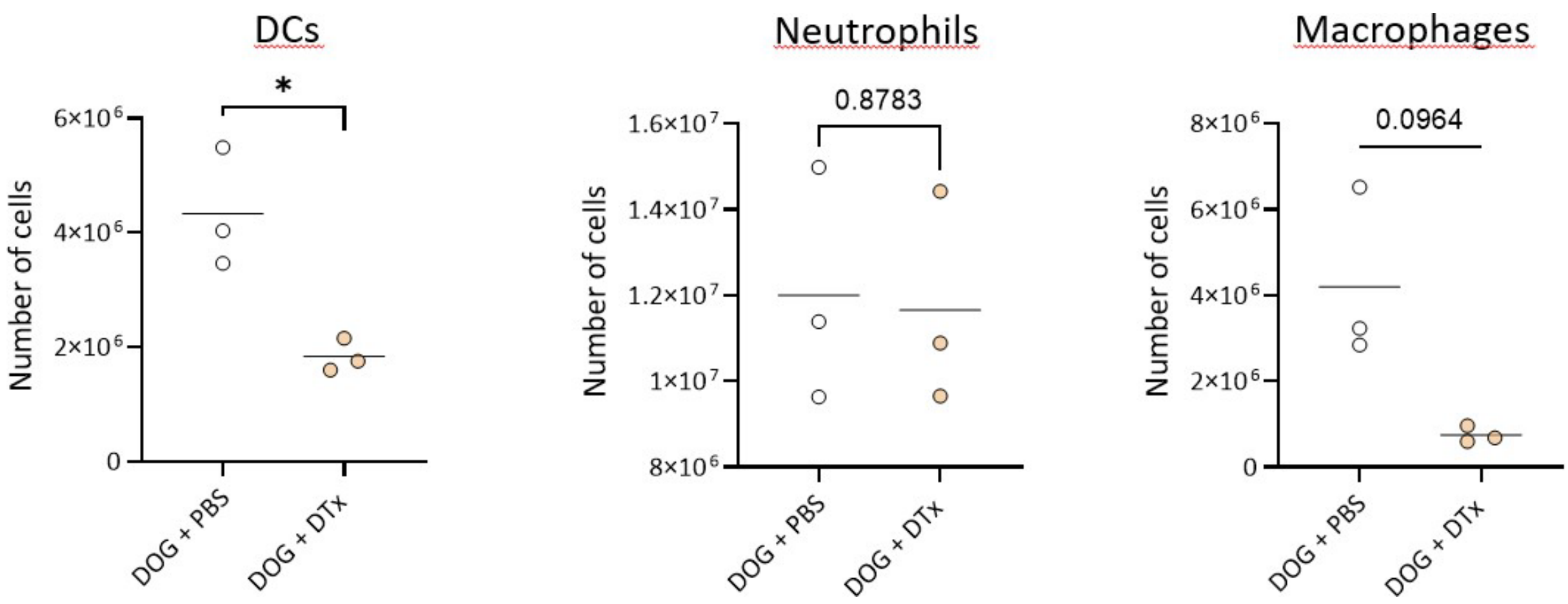


2. ***Mechanosensitive cell type identification.***

***Figures 5e and 5f are suggestive but not definitive that dermal CD11c⁺ cells are the highest Piezo1 expressers. Please sort major dermal populations and perform qPCR (and, if possible, protein) to compare Piezo1 and Piezo2 expression directly.***

As indicated above, we have analysed Piezo1 expression by FACS, and the results are included in the new Figure 5e and in the new Supplementary Fig. S7b).

The new data that we have generated are in agreement with the data present in the previous version of the manuscript showing the level of Piezo1 expression in the major dermal cell populations (endothelial, fibroblasts and myeloid cells) using IBEX multiplexed imaging. We report below a quantitative analysis of Piezo1 and Alox5 expression in CD11c$^+$, CD11c$^+$Zbtb46$^+$ (DCs), CD11c$^+$Zbtb46$^-$ (macrophages), PDGF$^+$ (fibroblasts), CD31$^+$ (endothelial) cells, and all dermal cells that do not belong to any of these lineages. These data show that, indeed, CD11c$^+$ cells are the dermal subpopulation with the higher Piezo1 and Alox5 levels. In comparison, keratinocytes express higher amounts of Piezo1 but lower amounts of Alox5. Yet, they are irrelevant in our model as explained above.

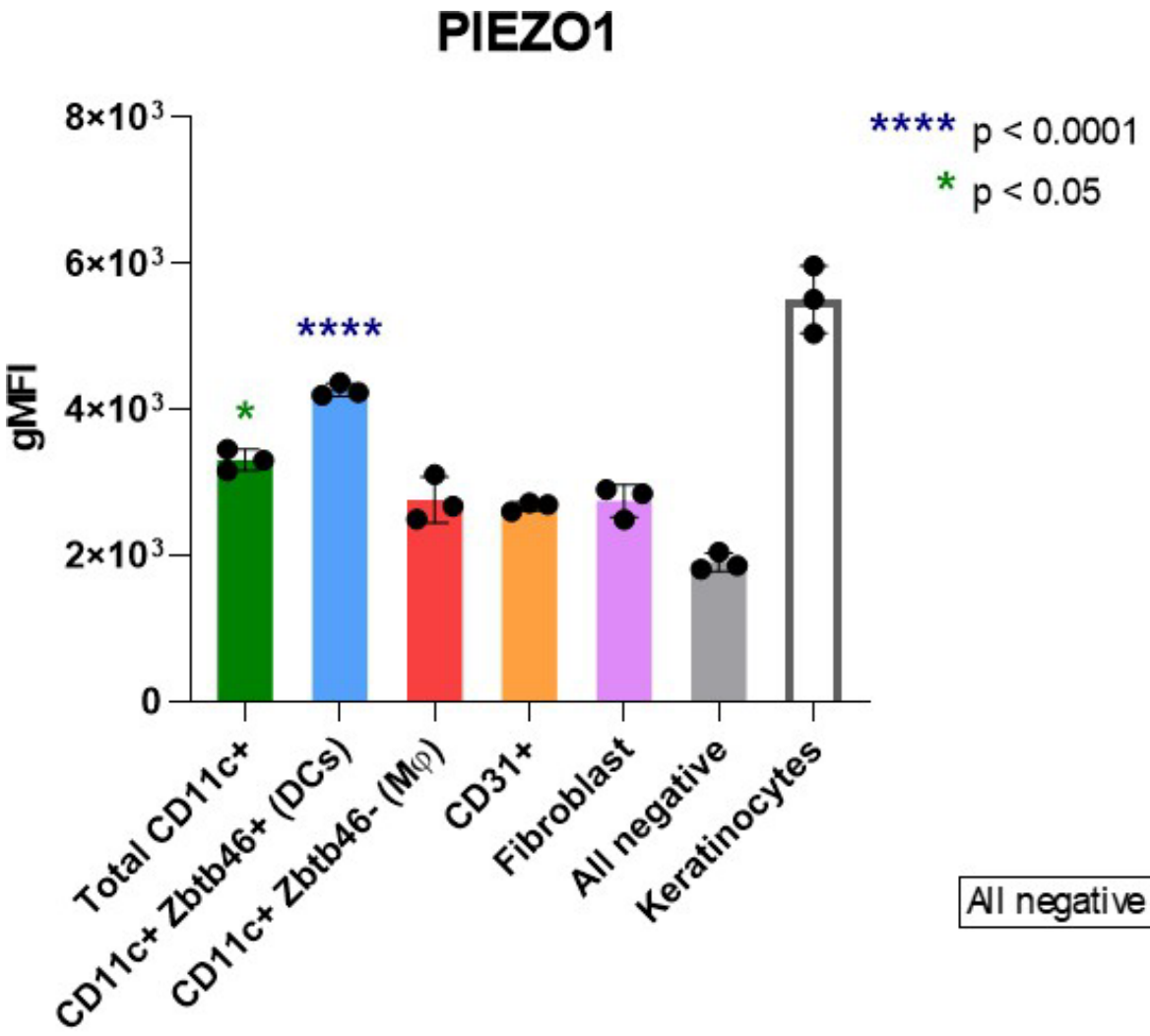


gMFI =geometric Mean Fluorescence Intensity

Number of cells

| | |
|---|---|
| CD11c+ | 279 |
| CD11c+Zbtb46+(DCs) | 115 |
| CD11c+Zbtb46- (Mφ) | 164 |
| CD31+ | 441 |
| Fibroblast | 518 |
| All negative | 683 |
| Keratinocytes | 754 |

| | |
|---|---|
| Number of mice analyzed | 3 |

| | |
|---|---|
| All negative cells: | CD45 - CD31 - PDGFRa - CD11c - Zbtb46 - CD3 - B220 - PanCK- |

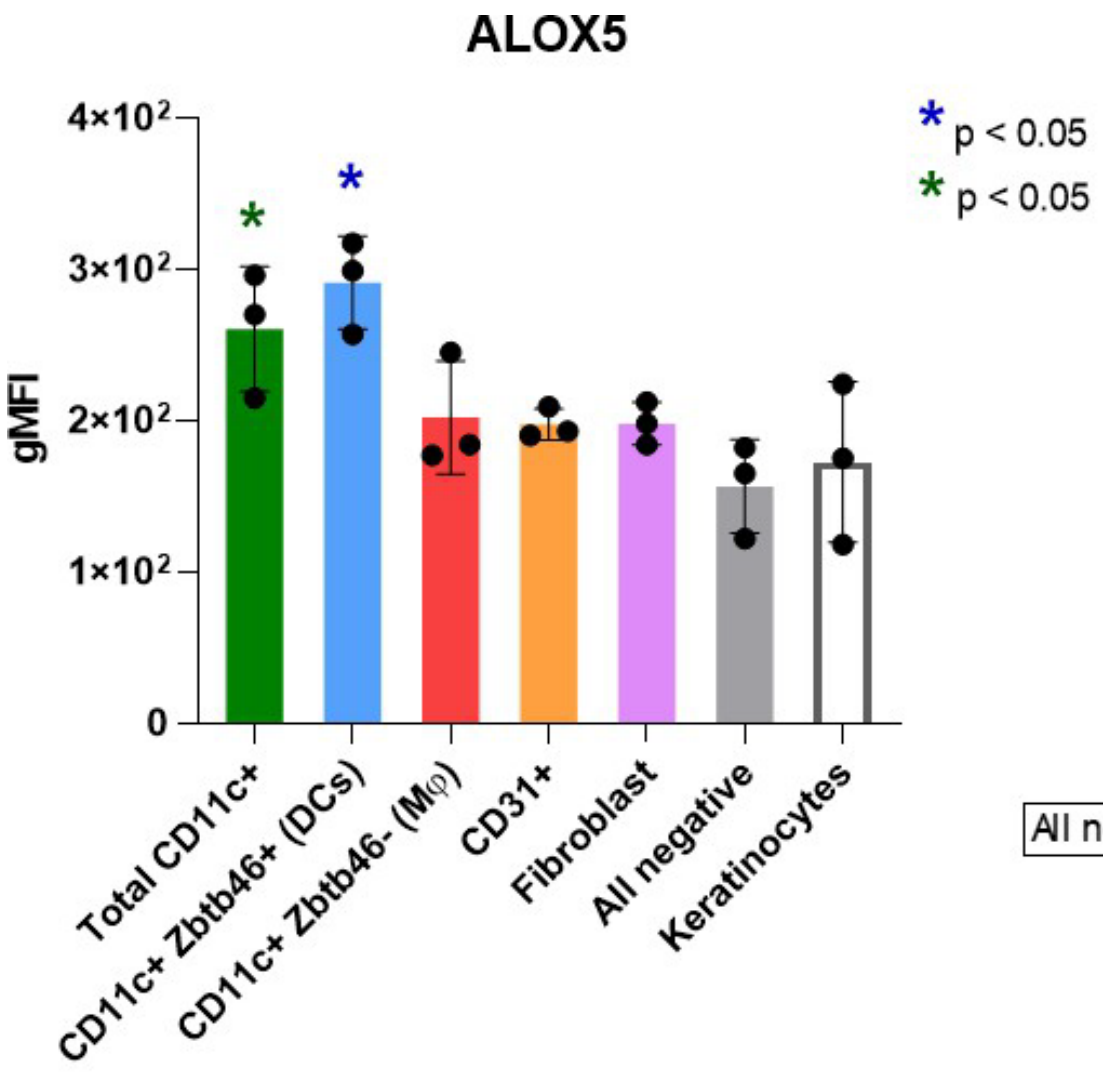


gMFI =geometric Mean Fluorescence Intensity

Number of cells

| | |
|---|---|
| CD11c+ | 279 |
| CD11c+Zbtb46+(DCs) | 115 |
| CD11c+Zbtb46- (Mφ) | 164 |
| CD31+ | 441 |
| Fibroblast | 518 |
| All negative | 683 |
| Keratinocytes | 754 |

| | |
|---|---|
| Number of mice analyzed | 3 |

| | |
|---|---|
| All negative cells: | CD45 - CD31 - PDGFRa - CD11c - Zbtb46 - CD3 - B220 - PanCK- |

***Use the same imaging or antibody pipeline to verify knockdown or knockout within CD11c⁺ cells after siRNA and local lenti-Cre. Demonstrating on-target loss in CD11c⁺ cells would alleviate concerns about delivery specificity.***

As requested by the reviewer we have analysed Piezo1 depletion in chimeric mice (in which only cells of hematopoietic origin are floxed) after administration of lenti-CRE, and we have observed deletion in CD11c+ cells (see new Fig. 5j and new Supplementary Fig. S9c)**.**

***The data in Supplementary Figure 7 are not comprehensive: Piezo1 is reported in macrophages, neurons, epithelia or keratinocytes, and, controversially, neutrophils; quantifying only DCs, endothelium, and fibroblasts is insufficient to claim DC-specific targeting by a broadly infective lentivector.***

We have replaced the previous experiments with the experiments performed in chimeric and CD11c-CRE x Piezo1$^{fl/fl}$ mice in which we have observed similar results. Supplementary Fig 7 has been replaced with Supplementary Fig. S9, which shows Piezo1 deletion in CD11c$^{+}$ cells.

***Please replace histograms with quantitative summaries and statistical tests of expression and knockout across relevant populations.***

We thank the reviewer for this observation. FACS analyses have been quantified, and the quantitative summaries have been included in all relevant figure panels in the new version of the manuscript (see new Fig. 5e and Supplementary Fig. S9).

3. ***Do adhesion mutants abrogate mechanosensation?***

***The pilC- and fnbA/B- experiments are compelling but do not, by themselves, show failure to activate Piezo1. Please add Ca2+ flux readouts (genetically encoded or dye based) during microbe to cell contact, showing: wild type microbes trigger Piezo1-dependent Ca2+ in wild type cells (lost with Piezo1 knockout), whereas adhesion mutants do not. This distinguishes mechanosensory failure from pleiotropic effects of the mutation on virulence, growth, or secretome. For example, does bacteria-free supernatant or heat killed WT bacteria activate the pathway? If so, this may not necessarily be via adhesion of live bacteria inducing membrane stretch. While, unlikely, perhaps another factor may activate the Piezo1 channel as has been controversy suggested by others (see Piezo in epithelial cells as a RNA sensor from the microbiome). Before concluding that, Piezo1 is responding to microbial***

***adhesion, direct downstream Piezo1 Ca2+ signaling will be required and is straightforward and tractable to test.***

We thank the reviewer for this important observation. We have performed calcium mobilization studies and included them in the revised version of the manuscript (see new Fig. 6h,i). In particular, we performed this experiment in D1 cells which express Piezo1 homogeneously (new Supplementary Fig. S10) and display a sufficiently synchronous response upon microbial stimulation to allow measurement of $Ca^{2+}$ fluxes by FACS analysis. $Ca^{2+}$ mobilization was significantly reduced in the presence of mutant compared to WT bacteria. Similarly, the Piezo inhibitor GsMTx-4 reduced $Ca^{2+}$ mobilization in cells exposed to bacteria or *C. albicans*, mainly affecting the sustained phase of the response. This pattern was consistent with that observed upon Yoda1 stimulation, where Piezo inhibition selectively impaired the sustained phase of $Ca^{2+}$ mobilization (new Fig.6h). Given the robust response induced by *P. aeruginosa,* we could measure $Ca^{2+}$ mobilization also in BMDCs, which are more asynchronous and heterogenous than D1 cells. In this case, we found an almost complete inhibition in Piezo1-deficient cells (new Fig. 6i).

***Also, in Figure 6f the differential TNFa requirement (Piezo1 dependent for Staphylococcus aureus but not Pseudomonas aeruginosa) needs clarification. Why would Piezo1 be required for what is a Myd88 dependent TNFa production in this context?***

This was surprising to us as well. The data are reproducible, but we do not currently have a mechanistic explanation for why Gram-positive, but not Gram-negative, bacteria induce TNFα secretion in a Piezo1- and MyD88-dependent manner. One possibility is that Piezo1 signaling integrates with PRR signaling, leading to sufficiently sustained NF-κB activation to promote TNFα production. However, this does not affect the validity of our model.

**Minor points**

1. ***"Upon adhesion, these microbes stretch the host cell membrane, exerting nanonewton mechanical forces that can activate Piezo channels45." In the cited work, it is reasonable to conclude that microbes can stretch the membrane, but it is not established that Piezo channels are sensitive to this specific interaction. Please refine the citation logic and language. Since it is the authors' novel hypothesis that this interaction activates Piezo channels, direct Ca2+ imaging should be added to test it.***

We have rephrased the text accordingly.

***2. Use of ns and non significant p values. In some cases the authors report a numeric p value for a non significant result (for example, Figure 6b) and in others only "ns." Please report the actual p value consistently in all cases. In Figure 6b, although non significant, the trend appears underpowered; additional replicates would likely yield significance while retaining the qualitative defect. Similarly, in Figure 6d, only three replicates were used; more biological replicates would likely achieve significance. Consistent reporting will help readers interpret these effects.***

We have inserted the actual p values and also added more experimental point to strength the statics in 6b. Concerning Figure 6d, the results were already clear. Hence, we could not request the use of additional mice to the Italian Ministry of Health.

**Reviewer #2**

*(Remarks to the Author)*

***The authors have revised their manuscript, but several key concerns from the initial review remain unaddressed. First, the authors use mutant bacteria to suggest that adhesion generates mechanical force. However, this constitutes circumstantial rather than direct evidence.***

We thank the reviewer for this important observation. We have performed calcium mobilization studies and included them in the revised version of the manuscript (see new Fig. 6h,i). In particular, we performed this experiment in D1 cells which express Piezo1 homogeneously (new Supplementary Fig. S10) and display a sufficiently synchronous response upon microbial stimulation to allow measurement of $Ca^{2+}$ fluxes by FACS analysis. $Ca^{2+}$ mobilization was significantly reduced in the presence of mutant compared to WT bacteria. Similarly, the Piezo inhibitor GsMTx-4 reduced $Ca^{2+}$ mobilization in cells exposed to bacteria or *C. albicans*, mainly affecting the sustained phase of the response. This pattern was consistent with that observed upon Yoda1 stimulation, where Piezo inhibition selectively impaired the sustained phase of $Ca^{2+}$ mobilization (new Fig.6h). Given the robust response induced by *P. aeruginosa,* we could measure $Ca^{2+}$ mobilization also in BMDCs, which are more asynchronous and heterogenous than D1 cells. In this case, we found an almost complete inhibition in Piezo1-deficient cells (new Fig. 6i).

***Secondly, they use imaging data to suggest that dendritic cells are the primary expressers of Piezo1; however, the presented images are unconvincing. The data lack sufficient detail and do not clearly explain how expression was quantified.***

We have now performed cytofluorimetric analyses of skin cells to verify the expression of Piezo1 and Piezo2. The results are included in the present version of the manuscript (new Fig. 5e and Supplementary Fig. S7). We found that Piezo1 is expressed at high levels in $CD11c^+$ cells and keratinocytes, and moderately in the endothelium. By contrast, Piezo2 expression is restricted to keratinocytes (new Fig. 5e and Supplementary Fig. S7b). Therefore, in the dermis - the tissue layer that constitutes the site of infection - only Piezo1 is expressed, primarily by $CD11c^+$ dendritic cells and macrophages.

***Third, it is surprising that the authors chose not to use the CD11c-Cre mouse model based on a single prior report suggesting a potential effect of CD11c on neutrophil maturation. This rationale seems inconsistent, especially considering that the authors used CD11c-DOG mice to deplete dendritic cells. If CD11c expression truly affects neutrophils, as they suggest, this concern would equally apply***

***to their current depletion strategy, potentially undermining their own data.***

We thank the reviewer for this observation. We generated a genetic model by backcrossing Piezo1/2$^{fl/fl}$ mice with CD11c-Cre mice. We selected Piezo1$^{fl/fl}$ x CD11c-Cre F2 mice and infected them with *C. albicans*. In this setting too, deletion of Piezo1 in CD11c$^{+}$ cells strongly reduced neutrophil recruitment (new Fig. 5k and Supplementary Fig. S9d).

To complement these experiments, and to exclude the possible involvement of non-immune cells expressing Piezo1 in the recruitment process, we generated bone marrow chimeras in which recipient DOG mice were transplanted with bone marrow cells from Piezo1/2$^{fl/fl}$ mice. Eighteen weeks after transplantation, mice were treated with DT, both i.v. and s.c., to eliminate any possible residual DOG DCs (see new Supplementary Fig. 9a). Analysis of chimerism showed that more than 90% of hematopoietic cells were of donor origin (new Supplementary Fig. S9b). The mice were then treated s.c. with lenti-Cre, and a clear ablation of Piezo1 in CD11c$^{+}$ cells was observed in the skin (see new Supplementary Fig. S9c). In this model, we found that $LTB_4$ production and neutrophil recruitment were significantly reduced following microbial infection (new Fig. 5j).

We are pleased that reviewer #1 wrote that the conclusions of he manuscript are now justified by the extensive new experiments presented.
We also thank reviewer #2 for their additional comments to which we provide a point-by-point reply below.

Reviewer #2

*……the central claim of the manuscript has shifted substantially: the cellular source of LTB4 has moved from stromal cells to CD11c+ myeloid cells.*
R: The previous revision of the manuscript already showed that $CD11c^+$ cells are the source of $LTB_4$ initiating the response to infection. The rationale for this change and the supporting data were detailed in the accompanying rebuttal letter.

*The cell-specific in vivo evidence for Piezo1 remains incomplete (Fig. 5j,k). The cleanest genetic experiment, CD11c-Cre × Piezo1fl, is shown only for C. albicans and only for neutrophil counts. It should be extended to S. aureus and P. aeruginosa, and should include the proximal readouts (LTB4, and ideally IL-1α and CXCL1), so that the genotype is tied to the proposed mechanism rather than to the terminal phenotype alone (increased neutrophil counts).*
R: We do not agree with the notion that CD11c-Cre approach is cleaner than the bone marrow transplantation followed by local lentiviral injection. Indeed, the latter strategy has the important advantage of restricting Piezo1 ablation to the infected tissue, thereby preserving the systemic functionality of immune cells, including neutrophils, which may themselves require Piezo1 to extravasate (see the rebuttal letter accompanying the first revision). Therefore, local Piezo1 ablation reduces the possibility that decreased neutrophil accumulation is secondary to a neutrophil-intrinsic defect in extravasation, rather than to loss of the initiating Piezo1-dependent $LTB_4$ signal from tissue-resident $CD11c^+$ myeloid cells.
In our intention, panels 5j and 5k were to confirm that our observations are independent of the specific Piezo1-ablation approach. For this reason, this proof of concept was performed using only *C. albicans*. However, we agree with this reviewer that $LTB_4$ is a more proximal readout than neutrophil accumulation. We have added the $LTB_4$ measurement in the new Fig. 5k (see also the bar graph below).

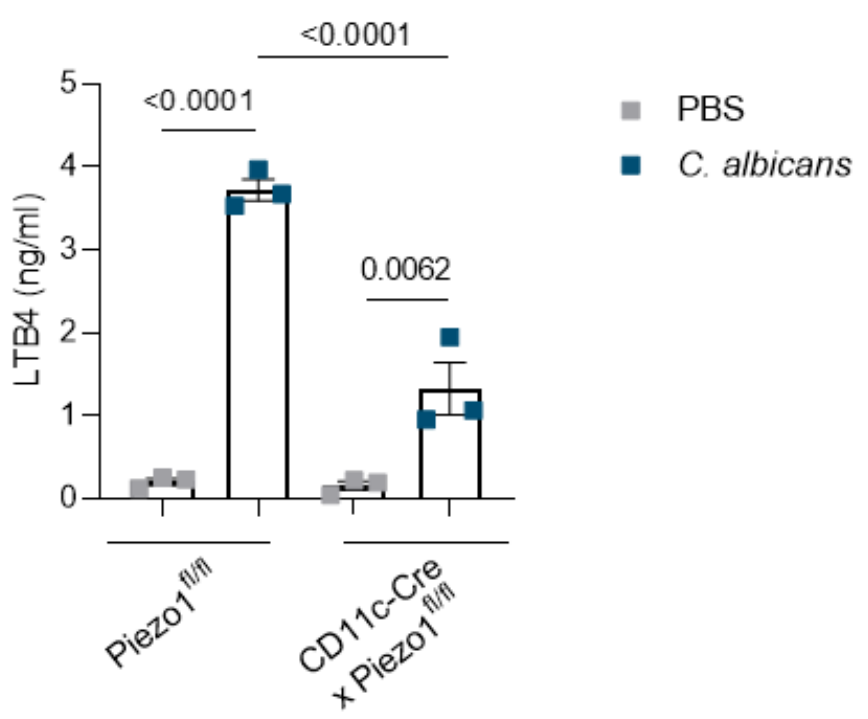


Concerning the other requests, we agree that IL-1α and CXCL1 are important components of the early recruitment module, as already shown in the manuscript. However, the key question here is whether the early burst of $LTB_4$ production, which initiates the immune response to infection, is functionally linked to Piezo1 expression in $CD11c^+$ myeloid cells. For this reason, tissue $LTB_4$ levels represent the most direct and informative proximal readout to include in the CD11c-Cre x $Piezo1^{fl/fl}$ setting.

*A more fundamental concern is the attribution of LTB4 to CD11c+ cells… Any intervention that lowers neutrophil numbers, given that LTB4 is itself a principal driver of neutrophil swarming and is produced by neutrophils in an autocrine and paracrine relay. …. will also reduce neutrophil-derived LTB4 as a secondary effect. The observed reductions therefore cannot be purely attributed to loss of an initiating CD11c+ source.*

R: We fully acknowledge that neutrophils produce $LTB_4$ and that, once recruited into the tissue, they can amplify $LTB_4$ production through the autocrine and paracrine relay described by Lämmermann et al., a study that we cited. We are therefore aware that tissue $LTB_4$ levels measured after neutrophil accumulation would reflect both the initiating source and neutrophil-dependent amplification.

Precisely for this reason, at the outset of the project we performed a series of time-course experiments to determine when neutrophils arrive at the site of infection and define the first wave of recruitment. This allowed us to identify the appropriate time point at which tissue $LTB_4$ should be measured. As shown in the figure below, at 2 hours post infection, neutrophil accumulation has not yet occurred. Therefore, the early $LTB_4$ signal measured at this time point cannot be explained by neutrophil-dependent amplification (see Figure below and new Supplementary Fig. S6a).

These data directly address the reviewer's concern and support the conclusion that the early $LTB_4$ signal precedes neutrophil recruitment. Thus, the reduction in tissue $LTB_4$ levels observed upon $CD11c^+$ cell depletion or $CD11c^+$ cell-restricted Piezo1 deletion reflects impairment of an upstream initiating pathway, rather than a secondary consequence of reduced neutrophil accumulation.

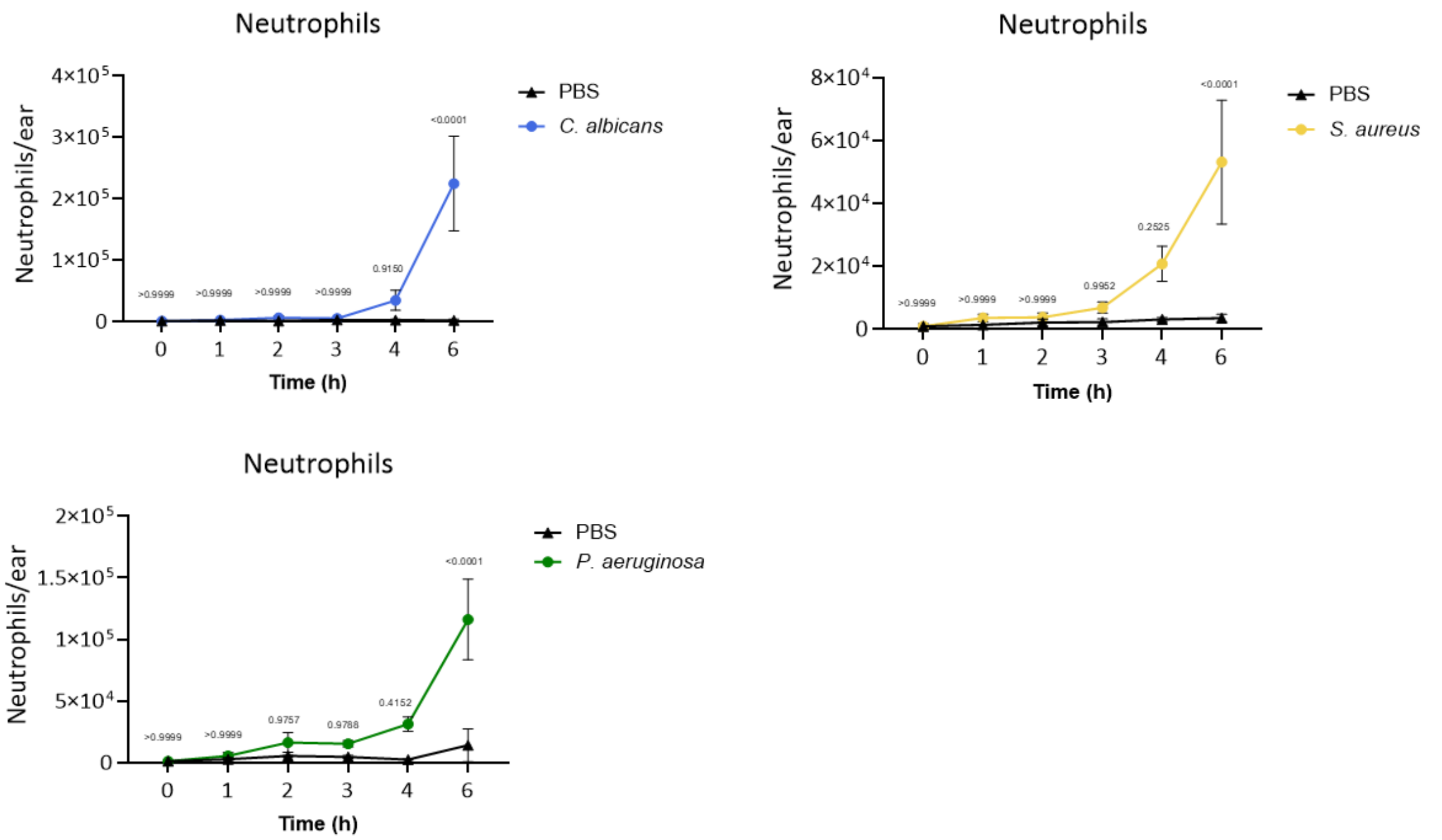


*…with the current data it is difficult to establish the two-step recruitment model definitively. Doing so would require cell-restricted manipulation of LTB4 synthesis to CD11c+ cells versus neutrophils, together with LTB4 measurements at early timepoints before substantial neutrophil influx.*

R: We respectfully disagree with the conclusion that the current data are insufficient to establish the two-step recruitment model, and with the view that cell-restricted manipulation of $LTB_4$ synthesis in $CD11c^+$ cells versus neutrophils is required to support this model.

Our model does not require CD11c$^+$ cells to be the exclusive source of $LTB_4$ throughout the inflammatory response. Rather, we propose that CD11c$^+$ myeloid cells provide the initiating Piezo1-dependent $LTB_4$ signal that triggers the first wave of neutrophil recruitment, after which recruited neutrophils may further increase $LTB_4$ levels locally.

Several lines of evidence support this interpretation. First, CD11c$^+$ dermal myeloid cells contain a Piezo1$^{high}$ Alox5$^{high}$ population, consistent with their ability to sense mechanical input and synthesize leukotrienes. Second, depletion of CD11c$^+$ cells removes this Piezo1$^{high}$ Alox5$^{high}$ population and markedly reduces early $LTB_4$ production and neutrophil recruitment. Third, *in vitro* BMDC experiments show that myeloid cells can produce $LTB_4$ directly in response to microbial adhesion, independently of MyD88 and in a Piezo1-dependent manner. Thus, the ability of CD11c$^+$ myeloid cells to generate $LTB_4$ is not inferred only *in vivo*, but is supported by cell-intrinsic mechanistic data.

Importantly, the results showing that neutrophils are not yet recruited at the time point $LTB_4$ is measured (see above) strengthen the conclusion that Piezo1 expression in CD11c$^+$ myeloid cells is required for neutrophil accumulation and provides the initiating $LTB_4$ signal, rather than being a secondary consequence of reduced neutrophil recruitment and ensuing loss of neutrophil-derived $LTB_4$.
